# Tumor Endothelial Inflammation Predicts Clinical Outcome in Diverse Human Cancers

**DOI:** 10.1371/journal.pone.0046104

**Published:** 2012-10-04

**Authors:** Sean P. Pitroda, Tong Zhou, Randy F. Sweis, Matthew Filippo, Edwardine Labay, Michael A. Beckett, Helena J. Mauceri, Hua Liang, Thomas E. Darga, Samantha Perakis, Sajid A. Khan, Harold G. Sutton, Wei Zhang, Nikolai N. Khodarev, Joe G. N. Garcia, Ralph R. Weichselbaum

**Affiliations:** 1 Department of Radiation and Cellular Oncology, The University of Chicago, Chicago, Illinois, United States of America; 2 Department of Medicine, The University of Illinois at Chicago, Chicago, Illinois, United States of America; 3 Department of Surgery, The University of Chicago, Chicago, Illinois, United States of America; National University of Ireland Galway, Ireland

## Abstract

**Background:**

Vascular endothelial cells contribute to the pathogenesis of numerous human diseases by actively regulating the stromal inflammatory response; however, little is known regarding the role of endothelial inflammation in the growth of human tumors and its influence on the prognosis of human cancers.

**Methods:**

Using an experimental model of tumor necrosis factor-alpha (TNF-α)-mediated inflammation, we characterized inflammatory gene expression in immunopurified tumor-associated endothelial cells. These genes formed the basis of a multivariate molecular predictor of overall survival that was trained and validated in four types of human cancer.

**Results:**

We report that expression of experimentally derived tumor endothelial genes distinguished pathologic tissue specimens from normal controls in several human diseases associated with chronic inflammation. We trained these genes in human cancer datasets and defined a six-gene inflammatory signature that predicted significantly reduced overall survival in breast cancer, colon cancer, lung cancer, and glioma. This endothelial-derived signature predicted outcome independently of, but cooperatively with, standard clinical and pathological prognostic factors. Consistent with these findings, conditioned culture media from human endothelial cells stimulated by pro-inflammatory cytokines accelerated the growth of human colon and breast tumors in immunodeficient mice as compared with conditioned media from untreated endothelial cells.

**Conclusions:**

This study provides the first prognostic cancer gene signature derived from an experimental model of tumor-associated endothelial inflammation. These findings support the notion that activation of inflammatory pathways in non-malignant tumor-infiltrating endothelial cells contributes to tumor growth and progression in multiple human cancers. Importantly, these results identify endothelial-derived factors that could serve as potential targets for therapy in diverse human cancers.

## Introduction

Over the last decade, our understanding of tumor biology has expanded to include host stromal elements as important determinants in malignant transformation and progression [1]. Therapies targeting stromal cells have, in part, resulted from the molecular characterization of tumor-stromal interactions. There is considerable evidence that stromal inflammation contributes to the proliferation and survival of malignant cells, facilitates genomic instability, stimulates angiogenesis and metastasis, and alters the response to anti-cancer therapies [2], [3]. When chronically produced in the tumor microenvironment, TNF-α is a major mediator of stromal inflammation [3]. TNF-α is important in early events in tumorigenesis, controlling a cascade of cytokines, chemokines, adhesion molecules, and pro-angiogenic activities [2], [3]. The most well-characterized actions of malignant cell-derived TNF-α are on vascular endothelial cells. Vascular endothelial cells actively participate in and regulate the inflammatory response in both normal and diseased tissues [4], and emerging data suggests that endothelial cells directly influence tumor behavior [5]–[7]. Nevertheless, little is known regarding the role of endothelial inflammation in promoting tumor growth and its influence on the prognosis of human cancers.

Gene expression profiling of clinical tumors has led to the discovery of numerous molecular signatures. One limitation of current gene expression profiling studies is a lack of validation in independent clinical datasets [8], [9]. Importantly, many empirically derived clinical signatures are specific to a single cancer type and often do not provide insight into relevant biological pathways affecting cancer prognosis. We utilized an experimental model of TNF-α-mediated inflammation to characterize inflammatory gene expression in tumor-associated endothelial cells. In this study, we demonstrate that the induction of inflammatory gene expression in tumor-associated endothelial cells significantly accelerates the growth of human tumors. Notably, we derive the first cancer gene signature associated with endothelial inflammation that predicts clinical outcome in four types of human cancers independently of standard clinical and pathological prognostic factors. Our findings provide a new biologically derived method of cancer prognostication and suggest potential pathways for the development of anti-cancer therapies targeting the tumor stroma.

## Methods

The methods used to culture cell lines and establish tumors in mice are described in detail in the Appendix S1, which is available with the full text of this article at www.plosone.org. Tumor-associated endothelial cells were isolated using a stepwise immunopurification of tumor tissue (Appendix S1 for full details). In brief, this was performed by clearance of hematopoietic cells from homogenized tumor tissue by incubation with biotin-labeled anti-CD19, anti-CD45 and anti-F4/80, which had been separately pre-bound to streptavidin-linked dynabeads (Dynal; Lake Success, NY), followed by removal of the bead-bound cells with a Dynal-50 magnet. Fc-Block (anti-CD16/32 antibodies) was added to the cell suspension to prevent non-specific binding of the Fc-receptor containing cells in the positive selection. Subsequently, anti-VE cadherin and anti-CD105 antibodies were added to bind endothelial cells, which was followed by the addition of streptavidin-linked dynabeads and capture with Dynal-50 magnet until only bead-bound cells remained in solution. The methods used for RNA extraction, microarray data collection and analysis, and quantitative real-time polymerase chain reaction are also described in the Appendix S1. Microarray data are available at Gene Expression Omnibus (GEO) [accession number: GSE33253] (http://www.ncbi.nlm.nih.gov/geo/query/acc.cgi?token=dfyldgammaawwjw&acc=GSE33253).

### Patient Sample Data

Patient information, including both clinical data and gene expression data, was obtained from multiple independent sources. Gene expression datasets representing inflammatory bowel disease (GSE13367), rheumatoid arthritis (GSE12021), and cirrhosis (GSE14323) were downloaded from GEO. These datasets were chosen based on the availability of both diseased and normal tissue specimens, as well as having a large number of samples available at the time of analysis. Gene expression datasets representing human breast cancer, colon cancer, lung cancer, and glioma were downloaded from publicly available repositories. These datasets were chosen based on the large number of samples, the availability of clinical outcome data, and the diversity of tumor types. For each tumor type, training and validation cohorts were constructed. In breast cancer [10] (n = 295; Netherlands Cancer Institute; http://bioinformatics.nki.nl/data.php) and colon cancer [11] (n = 232; Vanderbilt Medical Center (VMC; Nashville, TN) and H. Lee Moffitt Cancer Center (MCC; Tampa, FL); GSE17538 [177 from MCC; 55 from VMC]), the tumor samples were randomly separated into two parts (2/3 for training and 1/3 for validation) using computer-generated random numbers to assign specimens to training or validation cohorts. For glioma, distinct datasets [12], [13] were used for training (n = 77; University of California at San Francisco and MD Anderson Cancer Center; GSE4271) and validation (n = 50; Canadian Brain Tumor Tissue Bank (London, Ontario, Canada), Massachusetts General Hospital (Boston, MA), Brigham and Women's Hospital (Boston, MA), and Charite' Hospital (Berlin, Germany)); http://www.broadinstitute.org/cgi-bin/cancer/datasets.cgi). Lastly, for lung cancer [14], four datasets (n = 441) were available from a single study and separated into training (n = 257) and validation cohorts (n = 184) as was described in the original publication. These datasets were obtained from the University of Michigan Cancer Center, Moffitt Cancer Center, Memorial Sloan-Kettering Cancer Center and the Dana-Farber Cancer Institute (available at https://caarraydb.nci.nih.gov/caarray/publicExperimentDetailAction.do?expId1/41015945236141280. Clinical characteristics of the patients used for training and validation are listed in Table S1.

### Statistical Analysis

Affymetrix NetAffx Analysis Center was used to identify probe sets corresponding to human orthologs of the experimentally derived murine genes. No specific data preprocessing was performed before Significance Analysis of Microarrays (SAM) [15] was used to compare expression of the human orthologs in diseased and normal tissue samples. Differential expression was defined as a false discovery rate (FDR)-adjusted p value less than 0.05 and fold-change greater than 1.5. Genes differentially expressed in at least two of the three datasets tested were defined as mutually dysregulated. Hierarchical clustering via Ward's linkage rule with Euclidean distance metric using JMP 7.1 (SAS Institute Inc.; Cary, NC) was used to visualize gene expression differences.

In each cancer training dataset, univariate Cox proportional hazard regression was used to evaluate the association between survival and the level of expression of genes derived in the experimental microarray analysis that were differentially expressed in datasets of human diseases associated with chronic inflammation. Six of these forty-nine genes had concordant regression coefficients across all four tumor types. Expression of each individual gene was associated with an increased risk for death. For these six genes, a linear combination of the gene expression values weighted by the regression coefficients was used to calculate a risk score for each patient. The models and their associated scaling coefficients were fixed, based on the training groups, and then evaluated in the validation groups (Table S2). Letting e_ki_ denote the expression level of gene i for patient k, and letting w_i_ denote the weight for gene i, the gene score for patient k is s_k_ = Σw_i_(e_ki_-μ_i_)/τ_i_, where μ_i_ and τ_i_ are the mean and standard deviation of the gene expression values for gene i across all samples in the validation dataset. Patients were classified as having a high-risk gene signature or a low-risk gene signature, with the median of the risk score as the threshold value. A high score indicated a poor outcome. For each univariate Cox analysis, clinical and pathological factors associated with poor overall survival were identified among the available factors provided in the dataset. Factors significant on univariate analysis were entered into multivariate Cox analysis. Overall survival was analyzed and compared by the Kaplan-Meier method. Differences in survival were tested for statistical significance by the log-rank test. P values of less than 0.05 were considered to indicate statistical significance. Two-tailed P values were calculated for training datasets, while one-tailed P values were calculated for testing datasets. All statistical analyses were performed using JMP 7.1.

## Results

### Characterization of inflammatory gene expression in tumor-associated endothelium

**Figure 1:**
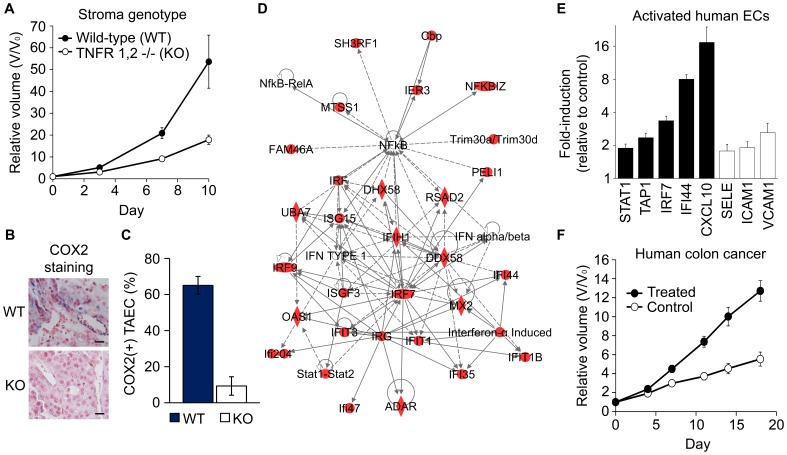
Inflammatory gene expression in tumor-associated endothelium is associated with increased tumor growth. (A) B16-F1 melanoma tumor growth was significantly suppressed in TNFR 1, 2−/− mice (KO) with disrupted stromal TNF-α signaling as compared to that in wild-type mice (WT). Tumor volume was measured relative to Day 0 volume, which was equal in WT and KO mice (p = 0.19; 2-tailed Student's t-test). Day 7, p = 0.002. Data are mean ± SEM. (B) Tumor-associated endothelial cells (TAECs) in KO mice have significantly reduced expression of the pro-inflammatory enzyme COX2. Representative images of immunohistochemistry for COX2 carried out on B16-F1 tumors (Day 7) and (C) quantification of COX2-positive TAECs. Scale bar, 20 µm. Data are mean ± SEM. P = 0.0014 (2-tailed Student's t-test). (D) WT TAECs overexpress a highly significant “inflammatory response” gene network (p = 10^−38^; Fisher's exact test). Solid lines represent direct relationships, while dashed lines represent indirect relationships. Red color indicates overexpression in WT TAECs. (E) Stimulation of human umbilical vein endothelial cells (HUVECs) with a combination of the pro-inflammatory cytokines TNF-α, IFNβ, and IFNγ induced the expression of both experimentally derived endothelial inflammatory genes (black bars), as well as, known markers of endothelial inflammation (white bars). Total RNA was analyzed by quantitative RT-PCR. Data are mean fold-change ± SEM relative to control-treated HUVECs. P<0.01 across genes (2-tailed Student's t-test). (F) Conditioned culture media from treated HUVECs accelerated the growth of human colon tumors xenografted in athymic mice. Pre-treated indicates prior incubation of tumor cells with conditioned culture media from stimulated HUVECs, while control indicates incubation with conditioned media from mock-treated HUVECs. Tumor volume was measured relative to Day 0 volume (8 days post-injection). Data are mean ± SEM. Day 18, p = 0.0009 (2-tailed Student's t-test).

To examine the role of TNF-α-mediated stromal inflammation in tumor growth, we used a syngeneic tumor model of B16-F1 murine melanoma established in wild-type (WT) mice and mice with immune dysfunction as a result of germline deletions of both TNF-α receptors (TNFR 1, 2−/−, herein referred to as knockout [KO]). Disruption of stromal TNF-α signaling significantly impaired the growth of tumors in KO mice as compared to that in WT mice (relative volume (V/V_0_) [mean ± SEM]; KO: 9.0±0.9; WT: 21±2.5; p = 0.0033; Fig. 1A), thus supporting a role for stromal TNF-α signaling in tumor growth. In association with these data, we found an increased expression of the pro-inflammatory enzyme, COX2, in tumor-infiltrating vessels of WT mice compared to KO mice, as measured by the percentage of COX2-positive vascular endothelial cells (WT: 65±4.9%; KO: 9.3±5.1%; p = 0.0014; Fig. 1B and C). In a replicate experiment, we purified endothelial cells from excised WT and KO tumors of equal volume and performed gene expression profiling of isolated tumor-associated endothelial cells (TAECs). We identified differential expression of 993 probe sets corresponding to 808 genes. WT TAECs exhibited increased expression of 686 probe sets and decreased expression of 307 probe sets relative to KO TAECs (Table S3). These genes encode enzymes (104), transcriptional regulators (66), kinases (35), transporters (20), and chemokines/cytokines (7). We utilized Ingenuity Pathway Analysis (IPA) to classify the differentially expressed genes and identified “Inflammatory Response” as the most significantly enriched set of functions overexpressed in WT TAECs. As measured by Fisher's exact test, the incidence of genes involved in inflammatory response functions was highly significant with p-values ranging from 2.4×10^−11^ to 7.4×10^−3^. IPA analysis demonstrated a significant linkage of this gene set to known TNF-α-related inflammatory pathways mediated by NF-κB and interferons (p = 10^−38^; Fig. 1D). These findings indicated that stromal TNF-α signaling contributes to accelerated tumor growth and inflammatory gene expression in tumor-associated endothelium.

We directly assessed endothelial gene expression in response to pro-inflammatory signals by stimulating cultured human umbilical vein endothelial cells (HUVECs) with a combination of TNF-α and interferons (IFN) β and γ. As determined by quantitative RT-PCR, expression of *VCAM1* (vascular cell adhesion molecule 1), *ICAM1* (inter-cellular adhesion molecule 1), and *SELE* (E-selectin), which are markers of activated endothelium [4], were induced 1.8- to 2.6-fold, respectively (Fig. 1E). In concert with these results, inflammatory marker genes overexpressed in WT TAECs, including *STAT1* (signal transducer and activator of transcription 1), *TAP1* (transporter, ATP-binding cassette, major histocompatibility complex 1), *IRF7* (interferon regulatory factor 7), *IFI44* (interferon-induced protein 44), and *CXCL10* (chemokine, CXC motif, ligand 10), were overexpressed 1.9- to 17.4-fold after treatment (Fig. 1E). We further tested the hypothesis that activated endothelial cells increase the growth of human tumors by incubating cultured tumor cells with conditioned culture media from either TNF-α/IFN-treated or control-treated HUVECs. Conditioned media from TNF-α/IFN-treated HUVECs significantly increased the growth of human colon tumor xenografts in athymic mice 2.3-fold as compared to conditioned media from control-treated HUVECs (p = 0.0009; Fig. 1F). In human breast tumors, we observed a similar 3.0-fold increase in tumor growth (p = 0.02) (data not shown). These findings demonstrated that endothelial gene expression in response to pro-inflammatory signals is associated with enhanced human tumor growth.

### Expression of tumor endothelial-derived inflammatory genes in human diseases associated with chronic inflammation

**Figure 2:**
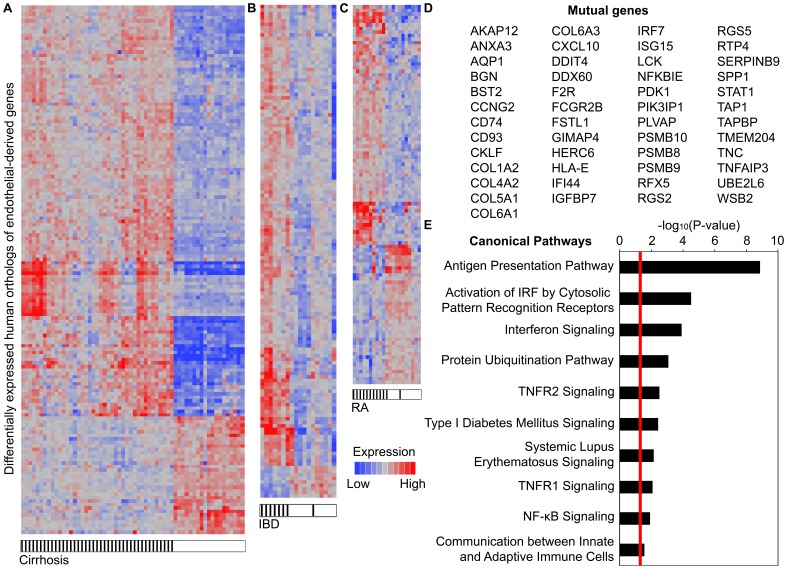
Tumor endothelium-derived genes are expressed in multiple human diseases of chronic inflammation. Expressional clustering of human orthologs of the tumor endothelium-derived genes in patient tissue samples of (A) cirrhosis (153 genes), (B) inflammatory bowel disease (IBD) (140 genes), and (C) rheumatoid arthritis (RA) (106 genes) compared to normal tissue controls. Within the cluster diagram, each column represents a patient sample and each row represents a differentially expressed gene. Diseased samples are denoted by black hatches. Expression is depicted as mean-normalized, log2-transformed values. (D) Forty-nine genes were mutually dysregulated in the datasets tested and concordant in expression with the experimental model. (E) Pathway analysis of the 49-gene set demonstrating significant over-representation of several inflammation-related pathways. P-values were calculated using Fisher's exact test. Red line indicates p = 0.05.

Endothelial inflammation is involved in the pathogenesis of many human diseases [16]–[18]. To determine whether experimentally derived inflammatory genes were relevant to human diseases, we measured the expression of 554 distinct human orthologs of the tumor endothelium-derived genes in datasets of human diseases associated with chronic inflammation. These genes significantly distinguished pathologic tissue specimens of cirrhosis (153 genes), inflammatory bowel disease (140 genes), and rheumatoid arthritis (106 genes) from normal tissue controls (Fig. 2A–C; Table S4). In particular, the sensitivities and specificities for these gene sets ranged from 88–100% and 90–100%, respectively (Table S5). A forty-nine-gene subset was mutually dysregulated in the datasets tested and concordant in expression with the experimental model (Fig. 2D). As expected, these genes were significantly enriched by pathways involved in the “Inflammatory Response” (p-value: 1.4×10^−12^–1.7×10^−2^) including antigen presentation, interferon signaling, TNFR 1 and 2 signaling, and NF-κB signaling (Fig. 2E). These results demonstrated that an inflammatory gene expression profile overexpressed in WT TAECs is differentially expressed in several human inflammatory diseases.

### Development of a prognostic molecular classifier

**Figure 3:**
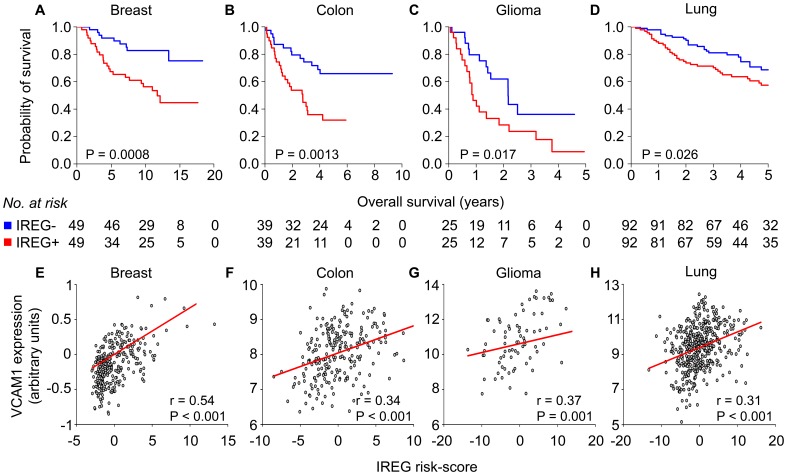
Expression of a tumor endothelium-derived gene signature predicts poor clinical outcome in multiple human cancers. IREG expression is associated with poor prognosis in (A) breast cancer (n = 98), (B) colon cancer (n = 78), (C) glioma (n = 50), and (D) lung cancer (n = 184). Kaplan-Meier survival curves for patient groups identified by IREG score. P-values indicate significant differences in overall survival as measured by log-rank tests. Red = IREG+, blue = IREG−. (E–H) Expression of the six-gene IREG score was positively correlated with VCAM1 gene expression in each tumor type. Shown is the Pearson correlation coefficient.

We next hypothesized that endothelial-derived inflammatory gene expression is predictive of tumor outcome in cancer patients. We used Cox proportional hazard regression across the forty-nine-gene set to identify six genes associated with reduced overall survival in each of four training datasets representing lung cancer (n = 257), breast cancer (n = 197), colon cancer (n = 154), and glioma (n = 77). We designated this six-gene set as the Inflammation-Related Endothelial-derived Gene (IREG) signature, which includes the genes *IFI44*, *TAP1*, *SPP1* (secreted phosphoprotein 1; also known as osteopontin), *ANXA3* (annexin A3), *RGS2* (regulator of G protein signaling 2), and *PDK1* (pyruvate dehydrogenase kinase, isoenzyme 1). We constructed a six-gene IREG score that combined gene expression with risk for death in the training datasets (Fig. S1). IREG+ patients were defined as those having a six-gene score greater than or equal to the group median score. In independent patient cohorts, we tested the ability of the six-gene score to classify patients into prognostic groups based on gene expression. Kaplan-Meier survival analysis comparing patient groups demonstrated a significantly reduced overall survival for IREG+ patients in independent cohorts of breast cancer (n = 98; p = 0.0008), colon cancer (n = 78; p = 0.0013), glioma (n = 50; p = 0.017), and lung cancer (n = 184; p = 0.026) (Fig. 3A–D). This association between IREG status and survival was confirmed by univariate Cox proportional hazard analysis of overall survival. IREG+ patients had an increased risk for death of 2.72-fold in colon cancer (p = 0.0027), 3.21-fold in breast cancer (p = 0.0015), 1.66-fold in lung cancer (p = 0.052), and 2.12-fold in glioma (p = 0.034) (Table 1). Notably, across cancer types IREG+ status was significantly associated with larger primary tumors of higher histological grade (Table 2). Furthermore, in each tumor dataset tested, we found a significant positive correlation between *VCAM1* gene expression and IREG score (Fig. 3E–H). These findings collectively indicated that the IREG signature is predictive of overall survival in multiple human cancers. Moreover, *VCAM1*, a marker of cytokine-activated endothelium, is co-expressed with the IREG signature.

**Table 1:** Univariate Cox proportional hazards regression of overall survival by IREG (+) status in training and testing cohorts.

		Training Set			Testing Set	
Cancer	*HR*	*95% CI*	*P-value*	*HR*	*95% CI*	*P-value*
Breast	1.90	1.06–3.54	0.032	3.21	1.54–7.31	0.0015
Colon	1.82	1.06–3.18	0.030	2.72	1.41–5.51	0.0027
Glioma	2.23	1.32–3.83	0.0025	2.12	1.06–4.38	0.034
Lung	1.44	1.06–1.97	0.021	1.66	1.00–2.81	0.052

Hazard ratio = HR. Confidence interval = CI.

**Table 2:** Comparison of patient characteristics by IREG signature expression.

	IREG(+)	IREG(−)	P-value
**Breast cancer**			
Age (years)			0.57
<40	34 (23)	29 (20)	
≥40	114 (77)	118 (80)	
Tumor size			0.0016
<T2	64 (43)	91 (62)	
≥T2	84 (57)	56 (38)	
Lymph nodes			0.82
Uninvolved	77 (52)	74 (50)	
Involved	71 (48)	73 (50)	
ER expression			<0.001
Negative	58 (39)	11 (7)	
Positive	90 (61)	136 (93)	
Tumor grade			<0.001
1, 2	63 (43)	113 (77)	
3	85 (57)	34 (23)	
**Colon cancer**			
Age (years)			0.78
<60	41 (35)	38 (33)	
≥60	75 (65)	78 (67)	
Stage			0.35
I, II	46 (40)	54 (47)	
III, IV	70 (60)	62 (53)	
Tumor grade			0.080
1, 2	90 (82)	93 (90)	
3	20 (18)	10 (10)	
**Glioma**			
Age (years)			0.0050
<55	25 (64)	35 (92)	
≥55	14 (36)	3 (8)	
**Lung cancer**			
Age (years)			0.63
<65	110 (50)	104 (47)	
≥65	111 (50)	116 (53)	
Lymph nodes			0.0076
Uninvolved	137 (62)	162 (74)	
Involved	83 (38)	56 (26)	
Tumor size			0.012
<T3	193 (87)	206 (95)	
≥T3	28 (13)	12 (5)	
Tumor grade			<0.001
1, 2	102 (47)	63 (29)	
3	117 (53)	152 (71)	

Shown are the number and percentages of patients in each category. For each clinical or pathological variable, p-values were calculated by Fisher's exact test comparing IREG (+) and IREG (−) patients.

### Multivariate analysis with standard clinical and pathological prognostic factors

In multivariate analyses using covariates available for each dataset, IREG+ status remained a significant covariate in breast cancer, lung cancer, and colon cancer in relation to standard clinical and pathological factors associated with poor prognosis (Tables S6, S7, S8, and S9). In these cancers, there were no significant multivariate interactions between IREG+ status and the other covariates. These results indicated that the prognostic effect of the IREG score was not dependent on specific values of the respective covariates. In breast cancer with poor prognosis (age <40 or tumor size ≥T2), we expanded this analysis by further stratifying patients by using the IREG score (Fig. S2). We determined that for each respective factor associated with poor prognosis, IREG+ patients had a 2.4- to 2.8-fold significantly elevated risk for death when compared to IREG− patients. A similar analysis of lung cancer patients with poor prognosis (age ≥65 or lymph node involvement) demonstrated a significant 1.4- to 2.0-fold greater risk for death in IREG+ patients (Fig. S3). For patients with stage 3 or 4 colon cancer, IREG+ patients had a 1.9-fold increased risk for death when compared to IREG− patients (Fig. S4). Lastly, for glioma patients at higher risk (age <55), IREG+ patients had a significantly increased risk for death of 2.4-fold, respectively (Fig. S5, Table S10). Taken together with the previous data, these results confirmed that IREG+ status enhances the identification of cancer patients at greater risk for death.

## Discussion

These findings suggest that endothelial inflammation is a mediator of tumor growth and progression. In support of this hypothesis, we demonstrate that the disruption of stromal TNF-α signaling suppresses inflammatory gene expression in tumor-associated endothelial cells and significantly impairs tumor growth. We further show that conditioned culture media from human endothelial cells activated by pro-inflammatory cytokines accelerates the growth of human tumors in immunodeficient mice. Finally, we derive a molecular signature reflective of tumor endothelial inflammatory gene expression that is highly predictive of poor clinical outcome in four types of human cancer. Concordant with our experimental model, patients with tumors that expressed these inflammatory genes had significantly larger primary tumors of higher histological grade.

Molecular signatures discovered through gene expression profiling have been shown to add prognostic value to clinical and pathological findings in several human cancers. Identifying prognostic variables that work cooperatively with known factors may improve the identification of patients at higher risk for relapse and death. Recently, several studies have identified host stromal signatures, either in purified stromal cells or from whole tumor samples, as significant prognostic factors in multiple types of human cancer including breast cancer, lung cancer, gastric cancer, prostate cancer, and lymphomas [19]–[26]. Finak et al [19] used laser capture microdissection (LCM) of primary breast tumors to construct a stroma-derived prognostic signature that predicted poor outcome in whole tumor-derived expression datasets. The authors found that poor outcome was strongly linked to the expression of numerous endothelial-derived genes and that patient samples within the poor outcome group had a significantly greater endothelial content than those in the good outcome group. Furthermore, Lenz et al [25] profiled gene expression in biopsy specimens from patients with diffuse large B-cell lymphoma and identified a highly prognostic stromal signature in patients with adverse outcome that was largely comprised of well-known endothelial markers. As well, Saadi et al [21] demonstrated that the progression from pre-malignant disease to esophageal adenocarcinoma was associated with a marked expression of inflammatory mediators in LCM stromal cells compromised, in part, by endothelial cells. These studies highlight the role of non-malignant tumor-infiltrating stromal cells in the prognosis of human cancers. In this regard, most tumor biopsies contain a significant fraction of stromal cells (up to 50% [10]). Therefore, signatures derived from whole tumor specimens reflect both tumor and stromal expression patterns. Nevertheless, few studies to date have identified prognostic molecular signatures relevant to multiple human cancers, and none of which were derived from tumor-associated endothelial cells.

Endothelial cells play an active role in a number of inflammatory functions that lead to increased blood flow, vascular leakage of plasma proteins, and leukocyte recruitment. Many successful therapies targeting chronic inflammation directly alter endothelial gene expression [4]. Specific examples include TNF-α inhibitors in rheumatoid arthritis and inflammatory bowel disease and statins in cardiovascular disease [4]. There is an increasing body of evidence that many malignancies are linked to diseases of chronic inflammation. One mechanism by which this occurs is through the induction and accumulation of DNA damage in proliferating cells by infiltrating inflammatory cells at sites of persistent inflammation. These changes lead to permanent genomic alterations that ultimately promote malignant transformation [2]. The strongest link between chronic inflammation and malignant disease is in colon carcinogenesis arising in individuals with inflammatory bowel diseases. While it is known that inflammatory pathways in other stromal cells also contribute to tumor growth [2], our results suggest that tumor-associated endothelial inflammation is an important determinant in tumor progression. In support of our findings, emerging evidence demonstrates that endothelial cell-derived signals, including inflammatory mediators, directly regulate tumor progression through “angiocrine” mechanisms independent of angiogenesis [27]. Nevertheless, further studies are needed to characterize the mechanisms by which inflamed tumor endothelial cells promote tumor growth.

Our findings differ from many empirically derived gene signatures in that we identified a molecular predictor of survival in patients with diverse human cancers based on an experimental model of tumor endothelial inflammation, which may prove useful biologically and clinically. Further prospective evaluation of the six-gene signature using RT-PCR may result in an accurate and reproducible prediction tool that may aid in clinical decision making across numerous human cancers. From a therapeutic perspective, the selective inhibition of endothelial-derived inflammatory factors, without disturbing the integrity of the blood vessels, might still block tumor growth and thereby avoid potential toxic side effects to the normal vasculature [6], [7]. Even more, it is known that angiogenic activity does not necessarily correlate with tumor prognosis [7]. Further investigation into the effect of endothelial inflammation on tumor growth could provide new targets for therapy in multiple human cancers.

## Supporting Information

Appendix S1 **Supplementary** **Methods** **and References.** Supplementary Methods include detailed descriptions of cell culture, tumor growth, tumor endothelial cell isolation, murine gene expression profiling, and quantitative RT-PCR of endothelial inflammatory genes with associated references listed in the Supplementary References.
(DOC)

Figure S1 **Application of the six-gene IREG signature to training datasets representing four human cancers.** Kaplan-Meier survival curves of patient groups defined by IREG score in breast cancer (n = 197), colon cancer (n = 154), lung cancer (n = 257), and glioma (n = 77). IREG+ was defined as a score greater than or equal to the group median score. P-values represent significance of log-rank tests for differences in overall survival comparing IREG+ and IREG− groups. Red = IREG+, blue = IREG−.
(TIF)

Figure S2 **IREG expression adds prognostic value to clinicopathologic factors associated with survival in human breast cancer.** Kaplan-Meier survival curves of patient cohorts grouped by (A) age (<40: n = 63 or ≥40: n = 232) or (B) tumor extent greater than or equal to T2 (n = 140) and further stratified by IREG score. P-values represent significance of log-rank tests for differences in overall survival comparing IREG+ and IREG− groups.
(TIF)

Figure S3 **IREG expression adds prognostic value to clinicopathologic factors associated with survival in human lung cancer.** Kaplan-Meier survival curves of patient cohorts grouped by (A) age (<65: n = 214 or ≥65: n = 227) or (B) lymph node involvement (n = 142) and further stratified by IREG score. P-values represent significance of log-rank tests for differences in overall survival comparing IREG+ and IREG− groups.
(TIF)

Figure S4 **IREG expression adds prognostic value to clinicopathologic factors associated with survival in human colon cancer.** Kaplan-Meier survival curves of patient cohorts grouped by clinical stage 3 or 4 (n = 132) and further stratified by IREG score. P-values represent significance of log-rank tests for differences in overall survival comparing IREG+ and IREG− groups.
(TIF)

Figure S5 **IREG expression adds prognostic value to clinicopathologic factors associated with survival in human glioma.** Kaplan-Meier survival curves of patient cohorts grouped by age (<55: n = 60) and further stratified by IREG score. P-value represents significance of log-rank test for differences in overall survival comparing IREG+ and IREG− groups.
(TIF)

Table S1 **Cancer patient characteristics for training and testing cohorts.** Shown are the percentages of patients in each category. For each clinical or pathological variable, p-values were calculated by Fisher's exact test comparing training and testing datasets.
(DOC)

Table S2 **Regression coefficients for the 49-gene set across cancer types.** Univariate Cox proportional hazard regression was used to evaluate the association between overall survival and gene expression for each of the 49 genes. Shown are the regression coefficients calculated for each training dataset. A positive value indicates an association with an increased risk for death.
(DOC)

Table S3 **Probe sets differentially expressed in tumor-associated endothelial cells (TAECs) derived from WT mice as compared to those in KO mice.** Probe Set ID corresponds to Affymetrix GeneChip® Mouse Genome 430 2.0 arrays. Expression values are presented as the ratio between WT and KO TAECs. Significance is indicated as a false-discovery rate-adjusted p-value.
(DOC)

Table S4 **Differential expression of human orthologs of tumor endothelium-derived genes in datasets of chronic inflammatory diseases.** Expression indicates the direction of gene expression in the experimental model (WT/KO TAECs) with UP signifying up-regulation and DOWN signifying down-regulation. Inflammatory bowel disease, IBD. Rheumatoid arthritis, RA. Cirrhosis, CIR. Genes with differential expression in diseased samples compared to normal tissue controls are indicated by a “1”. The 49 genes designated as mutually dysregulated and concordant in expression with the experimental model are indicated in the final column.
(DOC)

Table S5 **Classification values obtained by hierarchical clustering of tumor endothelial-derived genes in human inflammatory disease datasets.** PPV denotes positive predictive value, while NPV indicates negative predictive value.
(DOC)

Table S6 **Cox proportional hazard analysis of overall survival for 295 breast cancer patients.** The indicated model effects were used in the analysis. Age was considered a continuous variable. All other factors were considered as binary variables. Factors significant on univariate analysis were entered into multivariate and interaction (with IREG+) analyses. Hazard ratio = HR. Confidence interval = CI. Lymph node, LN.
(DOC)

Table S7 **Cox proportional hazard analysis of overall survival for 232 colon cancer patients.** The indicated model effects were used in the analysis. Age was considered a continuous variable. Stage (1–4) was considered an ordinal variable. IREG status was considered a binary variable. Factors significant on univariate analysis were entered into multivariate and interaction (with IREG+) analyses. Hazard ratio = HR. Confidence interval = CI.
(DOC)

Table S8 **Cox proportional hazard analysis of overall survival for 441 lung cancer patients.** The indicated model effects were used in the analysis. Age was considered a continuous variable. All other factors were considered as binary variables. Factors significant on univariate analysis were entered into multivariate and interaction (with IREG+) analyses. Hazard ratio = HR. Confidence interval = CI. Lymph node, LN.
(DOC)

Table S9 **Cox proportional hazard analysis of overall survival for 77 glioma patients.** The indicated model effects were used in the analysis. Age was considered a continuous variable. IREG status was considered a binary variable. Factors significant on univariate analysis were entered into multivariate and interaction (with IREG+) analyses. Hazard ratio = HR. Confidence interval = CI.
(DOC)

Table S10 **Univariate Cox proportional hazards model of overall survival using the IREG gene signature in patient subgroups.** Shown are the hazard ratios (HR), 95% confidence intervals (CI), and p-values.
(DOC)

Table S11 **Primers for quantitative RT-PCR analysis of human endothelial inflammatory gene expression.**
(DOC)
