# Supplementary material for: Tumor Endothelial Inflammation Predicts Clinical Outcome in Diverse Human Cancers
Source: PLoS One. 2012 Oct 4;7(10):e46104. doi: 10.1371/journal.pone.0046104 (PMC3464251; doi:10.1371/journal.pone.0046104)
Supplement: Appendix S1 — Supplementary Methods and References. Supplementary Methods include detailed descriptions of cell culture, tumor growth, tumor endothelial cell isolation, murine gene expression profiling, and quantitative RT-PCR of endothelial inflammatory genes with associated references listed in the Supplementary References. (DOC) [file pone.0046104.s001.doc]

**Supplementary Appendix**

**Tumor endothelial inflammation predicts clinical outcome in diverse human cancers**

Sean P. Pitroda^1^*, Tong Zhou^2^*, Randy F. Sweis^1^*, Matthew Filippo^1^, Edwardine Labay^1^, Michael A. Beckett^1^, Helena J. Mauceri^1^, Hua Liang^1^, Thomas E. Darga^1^, Samantha Perakis^1^, Sajid A. Khan^3^, Harold G. Sutton^1^, Wei Zhang^2^, Nikolai N. Khodarev^1†^, Joe G. N. Garcia^2†^, and Ralph R. Weichselbaum^1†^

^1^Department of Radiation and Cellular Oncology, The University of Chicago, Chicago, IL 60637; ^2^Department of Medicine, The University of Illinois at Chicago, Chicago, IL 60607; ^3^Department of Surgery, The University of Chicago, Chicago, IL 60637

*These authors made an equal contribution.

^†^These authors made an equal contribution.

**Supplementary Methods**

*Cell culture:* Tumor cell lines were obtained from the American Type Culture Collection (Manassas, VA). B16-F1 murine melanoma cells were cultured in RPMI 1640 medium. WiDr human colon cancer cells were cultured in Minimum Essential Medium. MDA-MB-231 human breast cancer cells were cultured Dulbecco’s Modified Eagle Medium with high glucose. Tumor culture media was purchased from Invitrogen (Carlsbad, CA) and supplemented with 10% fetal calf serum (Atlanta Biologicals; Lawrenceville, GA) and 1% penicillin/ streptomycin (Invitrogen). Human umbilical vein endothelial cells (HUVECs) were obtained from Lonza (Walkersville, MD) and cultured in EBM-2 supplemented with growth factors and fetal bovine serum (FBS) as provided in the EGM-2 BulletKit (Lonza). HUVECs were passaged according to Lonza protocol with Reagent Pack Subculture Reagents. Cell cultures were maintained at 37 °C in a humidified environment containing 5% CO_2_. For *in vitro* experiments, HUVECs were plated in EBM-2 medium with 5% FBS but without growth factors. HUVECs were left untreated or treated with a combination of human TNF-α (2 ng/ml) (Sigma-Aldrich; St. Louis, MO), human interferon β (1 ng/ml) (R&D Systems; Minneapolis, MN), and human interferon γ (30 pg/ml) (R&D Systems) for 3 hours and then washed 3 times with EBM-2 medium. Subsequently, HUVECs were incubated with fresh EBM-2 with 5% FBS for 48 hours. Conditioned media from treated and mock-treated HUVECs was centrifuged to remove cellular debris, and supernatants were collected and stored at -80 °C. For *in vivo* experiments, human tumor cells were cultured in conditioned HUVEC supernatant in a 1:1 mixture with complete tumor cell-specific medium for 48 hours prior to inoculation in athymic mice.

*Tumor growth:* C57BL/6 (C57BL/6-NCr) wild-type mice were obtained from FCRI-Taconic (Germantown, NY). TNFR 1, 2 -/- (B6;129S-*Tnfrsf1a^tm1Imx^Tnfrsf1b^tm1Imx^*/J) breeding pairs were obtained from Jackson Laboratories (Bar Harbor, ME). Mice were 8-12 weeks of age when experimentation began. Athymic mice were obtained from Harlan Laboratories (Madison, WI) and 6-8 weeks of age when experimentation began. The care and treatment of mice used in experiments was in accordance with institutional guidelines at the University of Chicago and specifically approved under the Institutional Animal Care and Use Committee [IACUC] Animal Care and Use Protocol [ACUP] number 70931. B16-F1 tumors were established following subcutaneous injection of 1-2x10^6^ cells in 100 μl of phosphate-buffered saline (PBS) into the right hind limbs of WT and KO mice. At the start of each tumor experiment (10 days post-injection) the mean tumor volume of B16-F1 tumors was equal in WT and KO mice (mean initial tumor volume (V_0_) ± S.E.M.; WT: 144 ± 44 mm^3^; KO: 209 ± 11 mm^3^; p = 0.19, 2-tailed Student’s t- test). This point was denoted as day 0 in the tumor growth curves. WiDr and MDA-MB-231 human tumor cells that were incubated with conditioned media from either treated or mock-treated HUVECs were subsequently injected at 10^6^ cells in 100 μl of PBS into the right hind limbs of athymic mice. Tumor volume was determined by direct measurement with calipers and estimated by using the formula (length x width x depth/2). Mice were euthanized using CO_2_ followed by cervical dislocation in accordance with institutional guidelines. B16-F1 tumors were collected and fixed in 10% formalin for 48 hours before embedded in paraffin. Four-micron paraffin sections for immunohistochemical staining were treated overnight at 4 °C with a 1:200 dilution of rabbit anti-mouse COX2 antibody (Novus; Littleton, CO). The percentage of COX2-positive vascular endothelial cells per high-powered field (400X) was averaged across 3-7 randomly selected visible vessels for each of 3 mice per group.

*Tumor endothelial cell isolation:* Tumor endothelial cells were isolated from day 0 WT (n = 15) and KO (n = 13) tumors when tumor volumes were equal. This process was based on a stepwise immunopurification of tumor tissue that had been pooled, minced and dissociated at 37 °C with 2 mg/ml of collagenase A in PBS (Roche; Indianapolis, IN) for 1 hour [28]. Cells were filtered through 100 µm and 25 µm mesh nylon filter fabric and pelleted in PBS with 0.5% bovine serum albumin (BSA) at 1000 x g for 5 minutes at 4 °C. Centrifugation was repeated until the supernatant was transparent (8-12 times). All subsequent steps were carried out on ice or at 4 °C. Samples were cleared of hematopoietic cells by incubation for 30 minutes at 4 °C with a 1:1:1 mixture of biotin anti-CD19, biotin anti-CD45 and biotin anti-F4/80, which had been separately pre-bound to streptavidin-linked dynabeads (Dynal; Lake Success, NY), followed by removal of the bead-bound cells with a Dynal-50 magnet. Fc-Block (anti-CD16/32 antibodies) (BD-Pharmingen; San Diego, CA) was added to the cell suspension to prevent non-specific binding of Fc-receptor containing cells in the positive selection. After an additional 30 minutes of 4 °C incubation, anti-VE cadherin and anti-CD105 antibodies were added to bind the endothelial cells. Following 30 minutes of 4 °C incubation, the cell suspension was washed 5 times with PBS/BSA. Streptavidin-linked dynabeads were added to the cell suspension, rotated for 5 minutes at 4 °C, captured with the Dynal-50 magnet, and washed 5-10 times with PBS/BSA until only bead-bound cells remained. Cells were then washed twice with complete media (RPMI 1640 with 10% FCS) with 1% penicillin/streptomycin and 1 µg/ml Fungizone (E.R. Squibb & Sons; Princeton, NJ) and resuspended in the same media. Cells were rested in culture at 37 °C and 5% CO_2_ for 1 hour before RNA isolation.

*Murine gene expression profiling*: RNA was collected and purified from confluent WT and KO tumor endothelial cell cultures using TRIzol reagent according to the manufacturer’s recommendations (Invitrogen). The quality of samples was assessed by using gel electrophoresis in 1.8% agarose and spectrophotometry. Samples of high quality were transferred to the Functional Genomics Facility of The University of Chicago for labeling and hybridization in duplicates with Affymetrix GeneChip® Mouse Genome 430 2.0 arrays. Retrieved data were scaled using MAS 5.0 suit (Affymetrix) and evaluated for chip quality using the Bioconductor Affy [29] and Simpleaffy [30] packages. All RNA samples and chips used in this study met established quality criteria (data not shown). Microarray data have been deposited in Gene Expression Omnibus (GEO) (<http://www.ncbi.nlm.nih.gov/geo/query/acc.cgi?token=dfyldgammaawwjw&acc=GSE33253>). The GC Robust Multi-array Average (GCRMA) function in the GCRMA package of Bioconductor [31] was applied to calculate the intensities of Affymetrix probe sets. Only the probe sets with “Present Calls” for both replicates in at least one group were used for further analysis. Differentially expressed probe sets between WT and KO tumor endothelial cells were identified by using Significance Analysis of Microarrays (SAM) [15]. The probe sets with a false discovery rate (FDR) < 0.1% and fold-change > 2 were considered as differentially expressed. The selected probe sets were annotated and functionally designated using Ingenuity Pathway Analysis (IPA) (Ingenuity Systems Inc.; Redwood City, CA). Fisher’s exact test was used to estimate the significance of the incidence of genes belonging to specific functional groups or networks. This method estimates the probability that the association between an experimental and reference gene set is due to random chance. A p-value ≤ 0.05 was considered statistically significant and indicates a nonrandom enrichment of an experimental dataset by members of a specific functional group.

*Quantitative RT-PCR of endothelial inflammatory genes*: RNA was extracted from HUVECs using Trizol reagent (Invitrogen). Total RNA (2 μg) was subjected to DNAse treatment using Invitrogen's Amplification Grade DNAse I Kit. cDNA was synthesized from one-half of the DNAse treated RNA using the High Capacity cDNA Reverse Transcription Kit (Applied Biosystems; Carlsbad, CA). cDNA reaction products were diluted 1:100 following synthesis. PCR was performed in a 384-well reaction plate on an Applied Biosystems 7900HT Sequence Detection System using Applied Biosystems 2X Power SYBR Green Master Mix in a 5 μl reaction volume containing 2 μl of diluted cDNA, 2.5 μl 2X Master Mix, and 0.5 μl 10uM primer mixture. Two sets of marker genes were selected for analysis. The first set included *VCAM1* (vascular cell adhesion molecule 1), *ICAM1* (intercellular adhesion molecule 1), and *SELE* (E-selectin), which encode for proteins with established pro-inflammatory functions in endothelial cells [3]. The second set included five experimentally derived tumor endothelium genes from our microarray analysis. These genes have known functions involved in inflammation and were among the genes with the largest differences in expression between WT and KO tumor endothelial cells. These five genes were *CXCL10* (C-X-C motif chemokine 10), *IFI44* (interferon-induced protein 44), *IRF7* (interferon regulatory factor 7), *STAT1* (signal transducer and activator of transcription 1), and *TAP1* (transporter 1, ATP-binding cassette, major histocompatibility complex, 1). Gene expression was quantified in relation to the expression of *GAPDH*. Primers for these genes are provided in Table S11.

**Supplementary References**

28. Seaman S, Stevens J, Yang MY, Logsdon D, Graff-Cherry C, et al (2007) Genes that distinguish physiological and pathological angiogenesis. Cancer Cell 11: 539-554.

29. Gautier L, Cope L, Bolstad BM, Irizarry RA (2004) affy--analysis of Affymetrix GeneChip data at the probe level. Bioinformatics 20: 307-315.

30. Wilson CL, Miller CJ (2005) Simpleaffy: a BioConductor package for Affymetrix Quality Control and data analysis. Bioinformatics 21: 3683-3685.

31. Wu Z, Irizarry RA, Gentleman R, Murillo FM, Spencer F (2004) A model based background adjustment for oligonucleotide expression arrays. J Am Stat Assoc 99: 909-917.
