# Supplementary figures and images for: Tumor Endothelial Inflammation Predicts Clinical Outcome in Diverse Human Cancers
Source: PLoS One. 2012 Oct 4;7(10):e46104. doi: 10.1371/journal.pone.0046104 (PMC3464251; doi:10.1371/journal.pone.0046104)

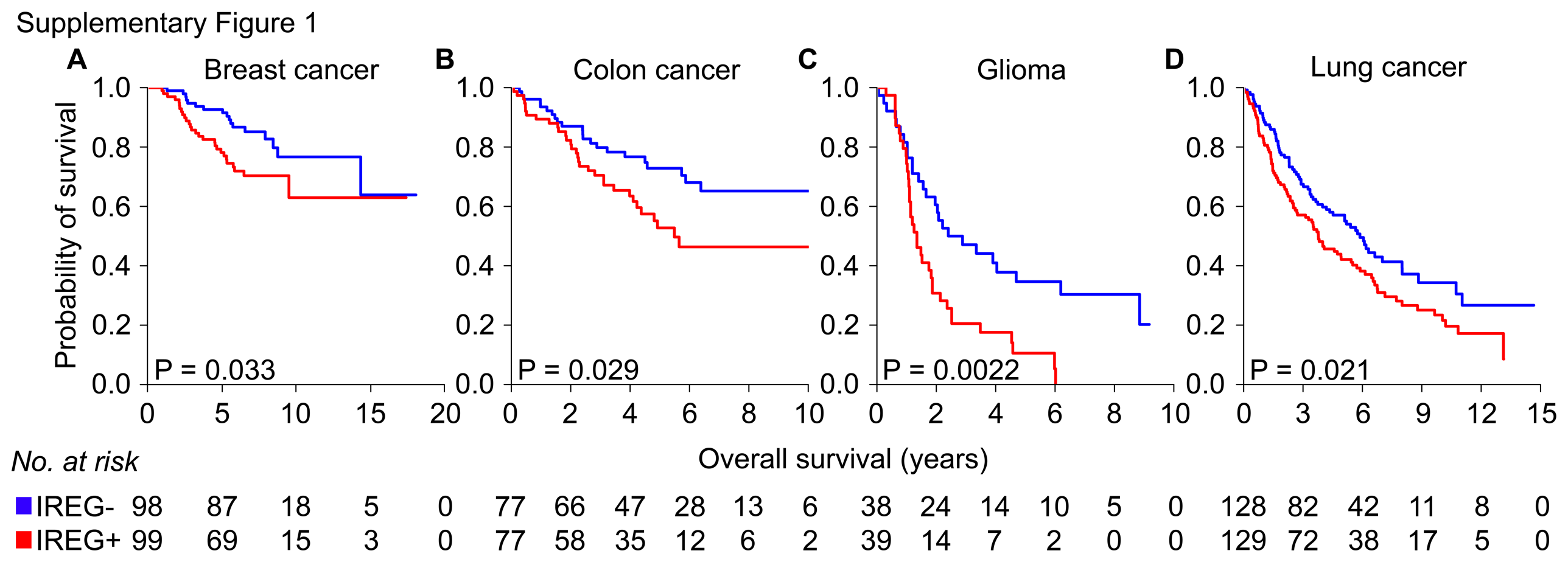

Supplement: Figure S1 — Application of the six-gene IREG signature to training datasets representing four human cancers. Kaplan-Meier survival curves of patient groups defined by IREG score in breast cancer (n = 197), colon cancer (n = 154), lung cancer (n = 257), and glioma (n = 77). IREG+ was defined as a score greater than or equal to the group median score. P-values represent significance of log-rank tests for differences in overall survival comparing IREG+ and IREG− groups. Red = IREG+, blue = IREG−. (TIF) [file pone.0046104.s002.tif]

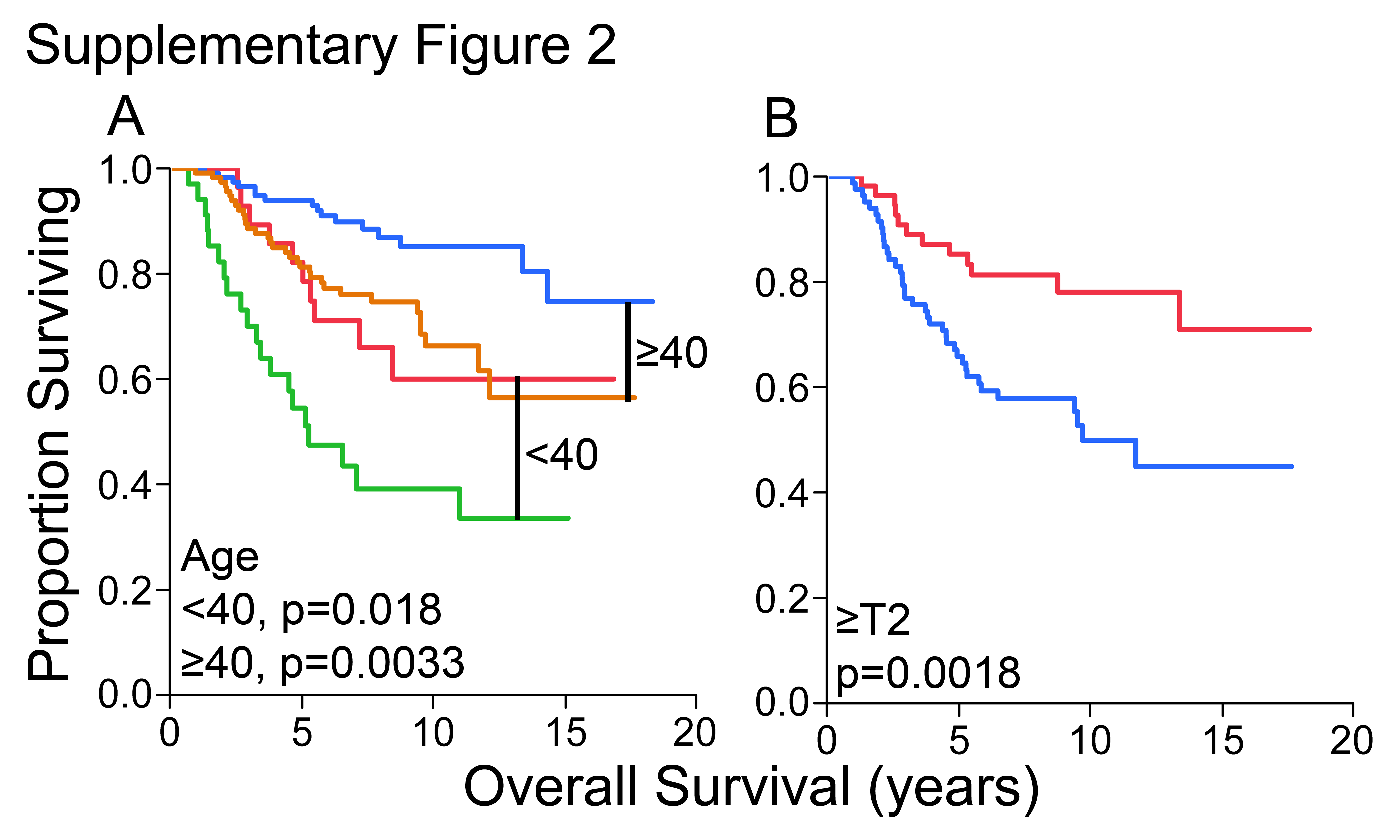

Supplement: Figure S2 — IREG expression adds prognostic value to clinicopathologic factors associated with survival in human breast cancer. Kaplan-Meier survival curves of patient cohorts grouped by (A) age (<40: n = 63 or ≥40: n = 232) or (B) tumor extent greater than or equal to T2 (n = 140) and further stratified by IREG score. P-values represent significance of log-rank tests for differences in overall survival comparing IREG+ and IREG− groups. (TIF) [file pone.0046104.s003.tif]

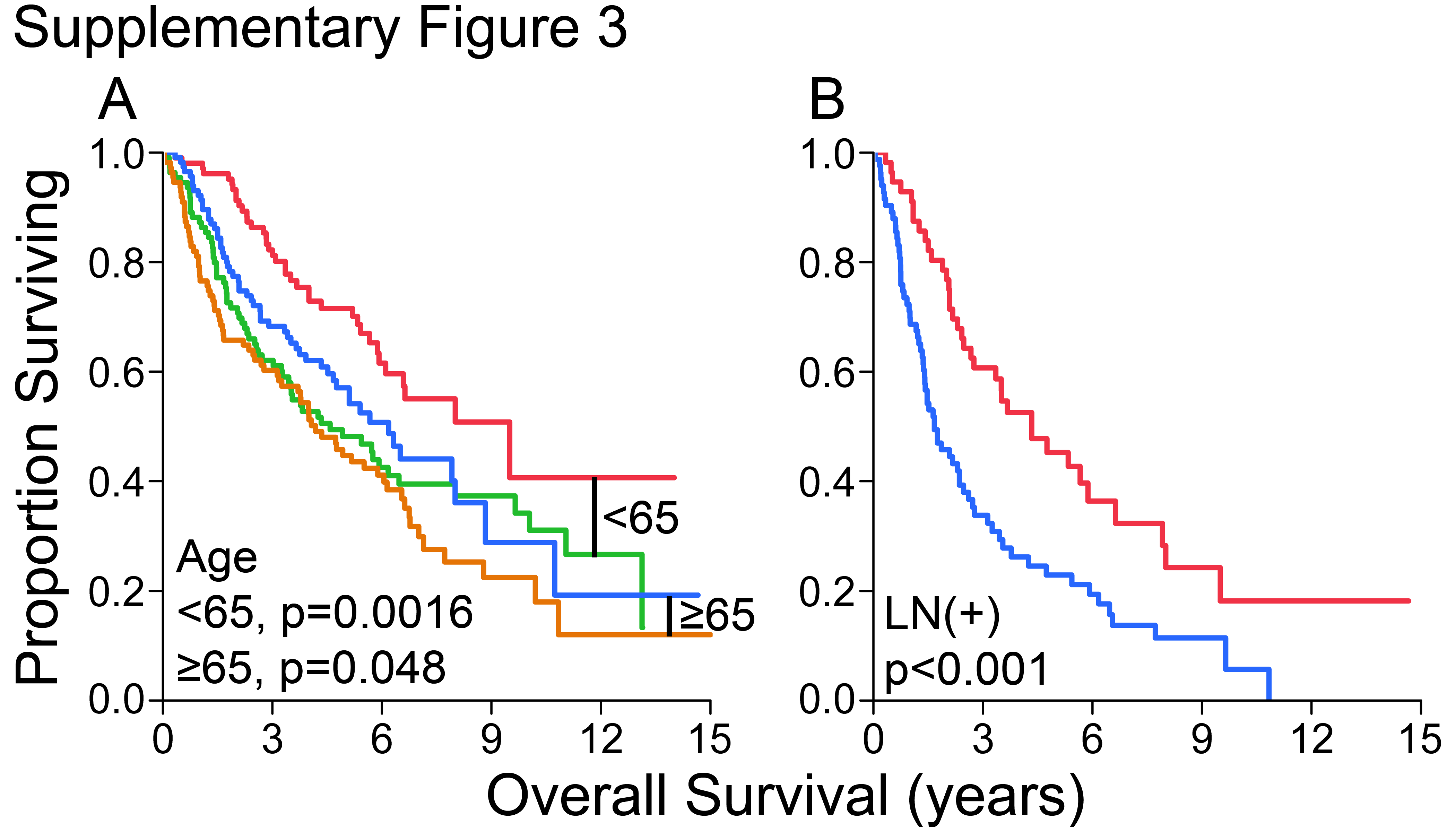

Supplement: Figure S3 — IREG expression adds prognostic value to clinicopathologic factors associated with survival in human lung cancer. Kaplan-Meier survival curves of patient cohorts grouped by (A) age (<65: n = 214 or ≥65: n = 227) or (B) lymph node involvement (n = 142) and further stratified by IREG score. P-values represent significance of log-rank tests for differences in overall survival comparing IREG+ and IREG− groups. (TIF) [file pone.0046104.s004.tif]

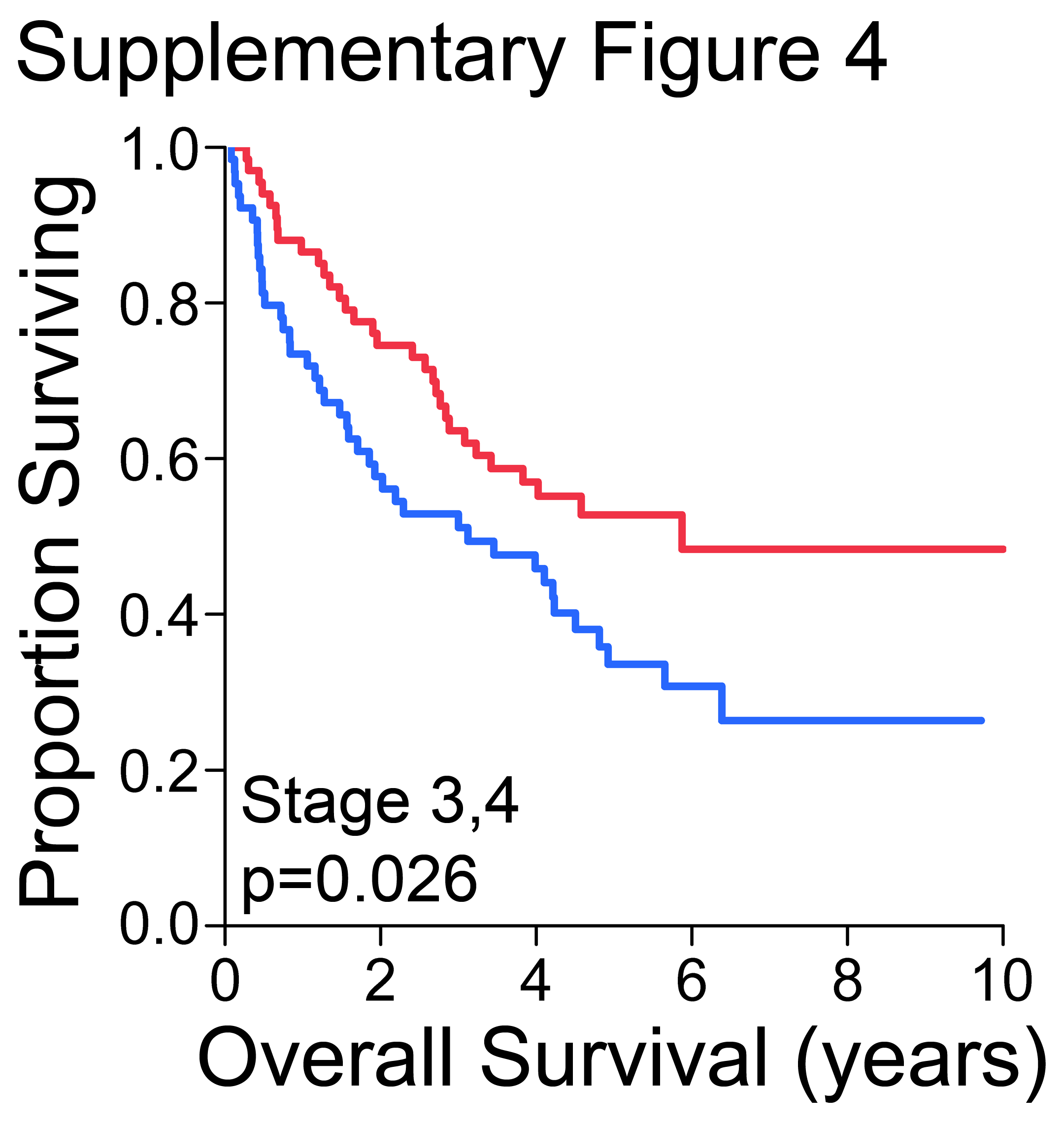

Supplement: Figure S4 — IREG expression adds prognostic value to clinicopathologic factors associated with survival in human colon cancer. Kaplan-Meier survival curves of patient cohorts grouped by clinical stage 3 or 4 (n = 132) and further stratified by IREG score. P-values represent significance of log-rank tests for differences in overall survival comparing IREG+ and IREG− groups. (TIF) [file pone.0046104.s005.tif]

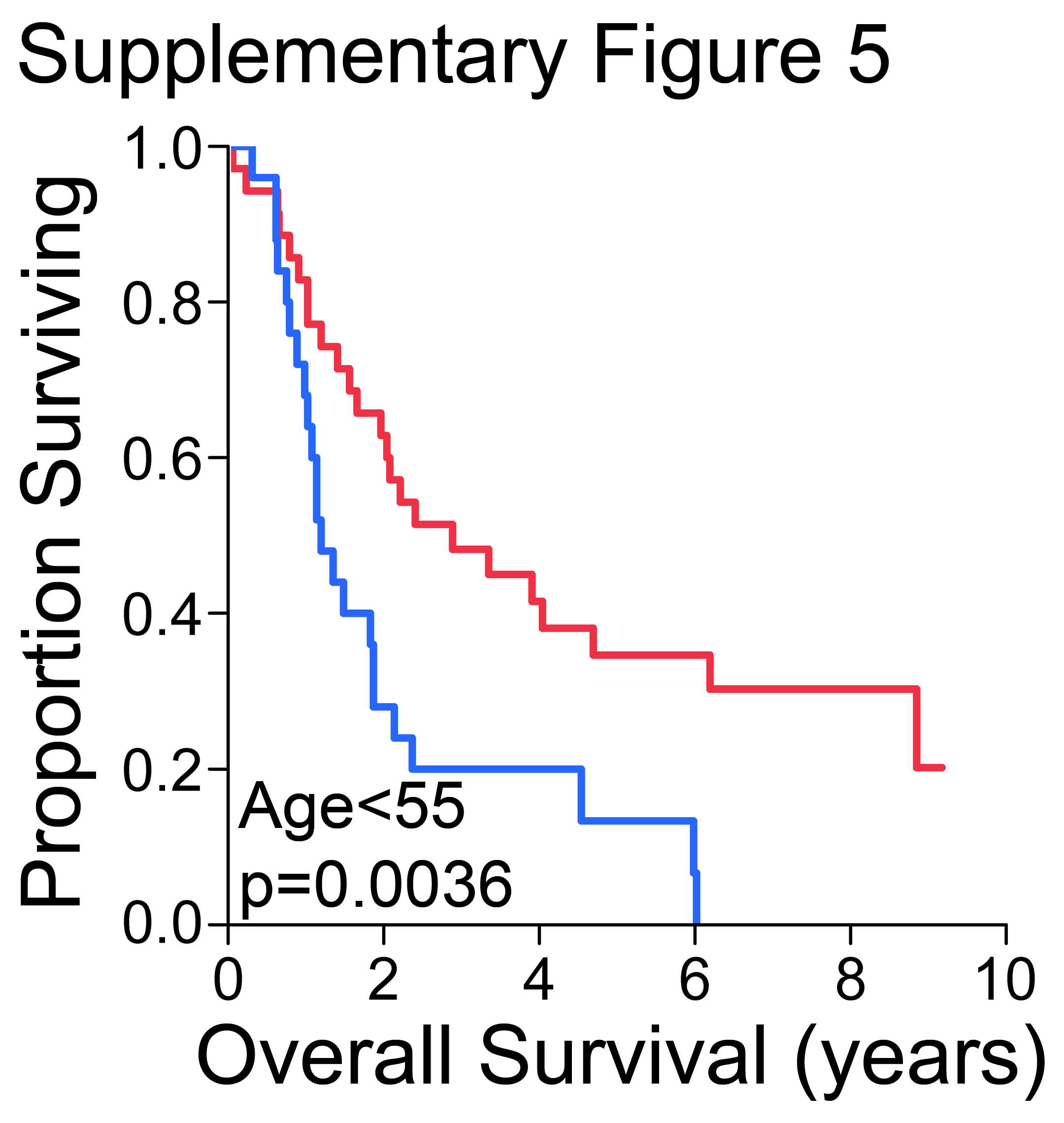

Supplement: Figure S5 — IREG expression adds prognostic value to clinicopathologic factors associated with survival in human glioma. Kaplan-Meier survival curves of patient cohorts grouped by age (<55: n = 60) and further stratified by IREG score. P-value represents significance of log-rank test for differences in overall survival comparing IREG+ and IREG− groups. (TIF) [file pone.0046104.s006.tif]
