# Supplementary material for: Tumor Endothelial Inflammation Predicts Clinical Outcome in Diverse Human Cancers
Source: PLoS One. 2012 Oct 4;7(10):e46104. doi: 10.1371/journal.pone.0046104 (PMC3464251; doi:10.1371/journal.pone.0046104)
Supplement: Table S1 — Cancer patient characteristics for training and testing cohorts. Shown are the percentages of patients in each category. For each clinical or pathological variable, p-values were calculated by Fisher's exact test comparing training and testing datasets. (DOC) [file pone.0046104.s007.doc]

|  |  | Training set |  | Testing set |  | P-value |
| --- | --- | --- | --- | --- | --- | --- |
| **Breast cancer** |  | n = 197 |  | n = 98 |  |  |
| Age (years) |  |  |  |  |  | 0.13 |
| < 40 |  | 19% |  | 27% |  |  |
| ≥ 40 |  | 81% |  | 73% |  |  |
|  |  |  |  |  |  |  |
| Tumor size |  |  |  |  |  | 0.80 |
| < T2 |  | 53% |  | 51% |  |  |
| ≥ T2 |  | 47% |  | 49% |  |  |
|  |  |  |  |  |  |  |
| Lymph nodes |  |  |  |  |  | 0.22 |
| Uninvolved |  | 54% |  | 46% |  |  |
| Involved |  | 46% |  | 54% |  |  |
|  |  |  |  |  |  |  |
| ER expression |  |  |  |  |  | 0.38 |
| Negative |  | 22% |  | 27% |  |  |
| Positive |  | 78% |  | 73% |  |  |
|  |  |  |  |  |  |  |
| Tumor grade |  |  |  |  |  | 0.13 |
| 1, 2 |  | 63% |  | 53% |  |  |
| 3 |  | 37% |  | 47% |  |  |
|  |  |  |  |  |  |  |
| **Colon cancer** |  | n = 154 |  | n = 78 |  |  |
| Age (years) |  |  |  |  |  | 0.14 |
| < 60 |  | 31% |  | 41% |  |  |
| ≥ 60 |  | 69% |  | 59% |  |  |
|  |  |  |  |  |  |  |
| Stage |  |  |  |  |  | < 0.001 |
| I, II |  | 53% |  | 25% |  |  |
| III, IV |  | 47% |  | 75% |  |  |
|  |  |  |  |  |  |  |
| Tumor grade |  |  |  |  |  | 0.19 |
| 1, 2 |  | 88% |  | 81% |  |  |
| 3 |  | 12% |  | 19% |  |  |
|  |  |  |  |  |  |  |
| **Lung cancer** |  | n = 257 |  | n = 184 |  |  |
| Age (years) |  |  |  |  |  | 0.043 |
| < 65 |  | 44% |  | 54% |  |  |
| ≥ 65 |  | 56% |  | 46% |  |  |
|  |  |  |  |  |  |  |
| Lymph nodes |  |  |  |  |  | 0.60 |
| Uninvolved |  | 69% |  | 67% |  |  |
| Involved |  | 31% |  | 33% |  |  |
|  |  |  |  |  |  |  |
| Tumor size |  |  |  |  |  | 0.0012 |
| < T3 |  | 87% |  | 96% |  |  |
| ≥ T3 |  | 13% |  | 4% |  |  |
|  |  |  |  |  |  |  |
| Tumor grade |  |  |  |  |  |  |
| 1, 2 |  | 67% |  | 55% |  | 0.012 |
| 3 |  | 33% |  | 45% |  |  |
|  |  |  |  |  |  |  |
| **Glioma** |  | n = 77 |  | n = 50 |  |  |
| Age (years) |  |  |  |  |  | NA |
| < 55 |  | 78% |  | NA |  |  |
| ≥ 55 |  | 22% |  | NA |  |  |
