# Supplementary material for: Tumor Endothelial Inflammation Predicts Clinical Outcome in Diverse Human Cancers
Source: PLoS One. 2012 Oct 4;7(10):e46104. doi: 10.1371/journal.pone.0046104 (PMC3464251; doi:10.1371/journal.pone.0046104)
Supplement: Table S2 — Regression coefficients for the 49-gene set across cancer types. Univariate Cox proportional hazard regression was used to evaluate the association between overall survival and gene expression for each of the 49 genes. Shown are the regression coefficients calculated for each training dataset. A positive value indicates an association with an increased risk for death. (DOC) [file pone.0046104.s008.doc]

| **Gene Symbol** | **Breast** | **Colon** | **Glioma** | **Lung** |
| --- | --- | --- | --- | --- |
| AKAP12 | -0.196 | 3.907 | -0.956 | 2.803 |
| ANXA3 | 0.124 | 0.503 | 0.538 | 1.231 |
| AQP1 | 0.412 | 0.094 | 3.467 | -1.064 |
| BGN | 1.587 | 2.141 | 0.415 | -0.775 |
| BST2 | -0.217 | 0.245 | 1.254 | -0.588 |
| CCNG2 | -3.001 | -0.420 | -1.501 | 1.133 |
| CD74 | 0.044 | -0.616 | 0.740 | -1.553 |
| CD93 | -0.448 | 1.629 | 1.189 | 0.259 |
| CKLF | 1.283 | 0.473 | 2.734 | -0.166 |
| COL1A2 | 0.043 | 3.223 | 1.208 | -0.524 |
| COL4A2 | 3.981 | 2.086 | 1.463 | -0.147 |
| COL5A1 | -0.652 | 2.512 | 1.196 | -0.332 |
| COL6A1 | 0.834 | 1.234 | 1.539 | -1.317 |
| COL6A3 | 1.412 | 3.032 | 0.249 | -0.767 |
| CXCL10 | 0.398 | -0.540 | 0.832 | 0.340 |
| DDIT4 | 1.537 | 3.097 | -1.281 | 2.374 |
| DDX60 | -0.147 | -0.029 | 1.697 | 1.342 |
| F2R | 1.350 | 0.660 | 2.089 | -0.664 |
| FCGR2B | 0.075 | 1.109 | 0.439 | -0.849 |
| FSTL1 | -0.065 | 2.050 | 2.970 | -1.244 |
| GIMAP4 | 0.342 | -1.071 | 0.069 | -1.393 |
| HERC6 | -0.453 | 0.372 | 0.594 | 0.473 |
| HLA-E | 0.859 | -0.424 | 0.873 | -0.211 |
| IFI44 | 0.402 | 0.445 | 1.481 | 0.548 |
| IGFBP7 | 0.176 | 1.690 | 2.362 | -0.872 |
| IRF7 | -0.095 | 0.770 | 1.644 | -0.677 |
| ISG15 | -0.105 | 1.443 | 0.673 | -0.068 |
| LCK | 1.830 | 0.070 | -0.559 | -1.332 |
| NFKBIE | 1.399 | -1.109 | -0.339 | -0.837 |
| PDK1 | 0.494 | 1.203 | 2.442 | 0.982 |
| PIK3IP1 | NA | -1.522 | -3.325 | -2.235 |
| PLVAP | NA | 1.339 | 0.336 | -1.524 |
| PSMB10 | 0.190 | -0.332 | 0.346 | -0.425 |
| PSMB8 | 1.249 | -0.966 | 1.521 | 0.971 |
| PSMB9 | 2.103 | -0.405 | 1.118 | 1.368 |
| RFX5 | -0.101 | 1.609 | 1.982 | 0.506 |
| RGS2 | 1.088 | 2.099 | 1.108 | 0.037 |
| RGS5 | -0.826 | 0.292 | -0.398 | -1.311 |
| RTP4 | NA | -1.210 | 0.563 | 0.917 |
| SERPINB9 | 0.798 | 2.226 | -0.372 | -0.935 |
| SPP1 | 1.181 | 1.892 | 0.965 | 0.532 |
| STAT1 | 0.991 | -0.667 | 1.760 | 0.352 |
| TAP1 | 1.397 | 0.191 | 1.047 | 2.666 |
| TAPBP | 0.973 | -0.161 | 0.241 | -0.099 |
| TMEM204 | NA | 0.915 | -0.442 | -1.346 |
| TNC | 0.625 | 2.480 | 2.230 | -0.208 |
| TNFAIP3 | 3.242 | 0.185 | 0.088 | -0.005 |
| UBE2L6 | 0.060 | -0.366 | -1.425 | 0.443 |
| WSB2 | 3.194 | 0.848 | -2.004 | 0.525 |
