# Supplementary material for: Tumor Endothelial Inflammation Predicts Clinical Outcome in Diverse Human Cancers
Source: PLoS One. 2012 Oct 4;7(10):e46104. doi: 10.1371/journal.pone.0046104 (PMC3464251; doi:10.1371/journal.pone.0046104)
Supplement: Table S3 — Probe sets differentially expressed in tumor-associated endothelial cells (TAECs) derived from WT mice as compared to those in KO mice. Probe Set ID corresponds to Affymetrix GeneChip® Mouse Genome 430 2.0 arrays. Expression values are presented as the ratio between WT and KO TAECs. Significance is indicated as a false-discovery rate-adjusted p-value. (DOC) [file pone.0046104.s009.doc]

| **Probe Set ID** | **WT/KO** | **FDR** | **Symbol** | **Entrez Gene Name** |
| --- | --- | --- | --- | --- |
| 1450783_at | 78.46 | 0.00000 | IFIT1B | interferon-induced protein with tetratricopeptide repeats 1B |
| 1420768_a_at | 70.01 | 0.00000 | DHX58 | DEXH (Asp-Glu-X-His) box polypeptide 58 |
| 1423555_a_at | 69.41 | 0.00000 | IFI44 | interferon-induced protein 44 |
| 1449025_at | 62.43 | 0.00000 | IFIT3 | interferon-induced protein with tetratricopeptide repeats 3 |
| 1439114_at | 55.47 | 0.00000 | DDX60 | DEAD (Asp-Glu-Ala-Asp) box polypeptide 60 |
| 1417244_a_at | 54.14 | 0.00000 | IRF7 | interferon regulatory factor 7 |
| 1456494_a_at | 52.88 | 0.00000 | Trim30a/Trim30d | tripartite motif-containing 30A |
| 1420380_at | 52.44 | 0.00000 | CCL13 | chemokine (C-C motif) ligand 13 |
| 1451426_at | 50.28 | 0.00000 | DHX58 | DEXH (Asp-Glu-X-His) box polypeptide 58 |
| 1449009_at | 47.60 | 0.00000 | Tgtp1 | T-cell specific GTPase 1 |
| 1417292_at | 45.76 | 0.00000 | Ifi47 | interferon gamma inducible protein 47 |
| 1450484_a_at | 41.44 | 0.00000 | CMPK2 | cytidine monophosphate (UMP-CMP) kinase 2, mitochondrial |
| 1419043_a_at | 37.95 | 0.00000 | Iigp1/Iigp1b | interferon inducible GTPase 1 |
| 1451905_a_at | 37.15 | 0.00000 | MX1 | myxovirus (influenza virus) resistance 1, interferon-inducible protein p78 (mouse) |
| 1449984_at | 29.86 | 0.00000 | CXCL3 | chemokine (C-X-C motif) ligand 3 |
| 1426906_at | 29.52 | 0.07195 | Mndal | myeloid nuclear differentiation antigen like |
| 1438868_at | 28.71 | 0.00000 | D14Ertd668e | DNA segment, Chr 14, ERATO Doi 668, expressed |
| 1431591_s_at | 27.27 | 0.00000 | ISG15 | ISG15 ubiquitin-like modifier |
| 1438676_at | 27.23 | 0.00000 | GBP6 | guanylate binding protein family, member 6 |
| 1418392_a_at | 25.88 | 0.00000 | GBP4 | guanylate binding protein 4 |
| 1418240_at | 24.11 | 0.00000 | GBP2 | guanylate binding protein 2, interferon-inducible |
| 1435906_x_at | 23.15 | 0.00000 | GBP2 | guanylate binding protein 2, interferon-inducible |
| 1451567_a_at | 21.77 | 0.00000 | Ifi203 | interferon activated gene 203 |
| 1452231_x_at | 20.05 | 0.00000 | Mndal | myeloid nuclear differentiation antigen like |
| 1417141_at | 19.19 | 0.00000 | Igtp | interferon gamma induced GTPase |
| 1418191_at | 18.75 | 0.00000 | USP18 | ubiquitin specific peptidase 18 |
| 1421008_at | 18.60 | 0.00000 | RSAD2 | radical S-adenosyl methionine domain containing 2 |
| 1440481_at | 18.32 | 0.07195 | STAT1 | signal transducer and activator of transcription 1, 91kDa |
| 1425156_at | 17.35 | 0.00000 | GBP7 | guanylate binding protein 7 |
| 1418580_at | 17.19 | 0.00000 | RTP4 | receptor (chemosensory) transporter protein 4 |
| 1437308_s_at | 16.99 | 0.00000 | F2R | coagulation factor II (thrombin) receptor |
| 1452349_x_at | 16.32 | 0.00000 | Ifi204 | interferon activated gene 204 |
| 1457644_s_at | 16.10 | 0.00000 | CXCL2 | chemokine (C-X-C motif) ligand 2 |
| 1417961_a_at | 15.98 | 0.00000 | Trim30a/Trim30d | tripartite motif-containing 30A |
| 1436058_at | 15.92 | 0.00000 | RSAD2 | radical S-adenosyl methionine domain containing 2 |
| 1426276_at | 15.73 | 0.00000 | IFIH1 | interferon induced with helicase C domain 1 |
| 1421009_at | 15.50 | 0.00000 | RSAD2 | radical S-adenosyl methionine domain containing 2 |
| 1417172_at | 14.90 | 0.00000 | UBE2L6 | ubiquitin-conjugating enzyme E2L 6 |
| 1451860_a_at | 14.81 | 0.00000 | Trim30a/Trim30d | tripartite motif-containing 30A |
| 1452348_s_at | 14.45 | 0.00000 | Ifi203 | interferon activated gene 203 |
| 1459137_at | 13.58 | 0.00000 | PML | promyelocytic leukemia |
| 1419186_a_at | 13.55 | 0.07195 | ST8SIA4 | ST8 alpha-N-acetyl-neuraminide alpha-2,8-sialyltransferase 4 |
| 1456046_at | 13.12 | 0.07195 | CD93 | CD93 molecule |
| 1419209_at | 12.51 | 0.00000 | CXCL2 | chemokine (C-X-C motif) ligand 2 |
| 1451564_at | 12.04 | 0.00000 | PARP14 | poly (ADP-ribose) polymerase family, member 14 |
| 1434380_at | 12.04 | 0.00000 | GBP7 | guanylate binding protein 7 |
| 1425582_a_at | 11.92 | 0.00000 | EMCN | endomucin |
| 1437176_at | 11.42 | 0.00000 | Nlrc5 | NLR family, CARD domain containing 5 |
| 1450033_a_at | 11.28 | 0.00000 | STAT1 | signal transducer and activator of transcription 1, 91kDa |
| 1443858_at | 10.79 | 0.00000 | TRIM5 | tripartite motif containing 5 |
| 1445271_at | 10.62 | 0.00000 | TRIM5 | tripartite motif containing 5 |
| 1436562_at | 10.46 | 0.00000 | DDX58 | DEAD (Asp-Glu-Ala-Asp) box polypeptide 58 |
| 1417793_at | 10.35 | 0.00000 | Irgm2 | immunity-related GTPase family M member 2 |
| 1450034_at | 9.64 | 0.00000 | STAT1 | signal transducer and activator of transcription 1, 91kDa |
| 1456890_at | 9.40 | 0.00000 | DDX58 | DEAD (Asp-Glu-Ala-Asp) box polypeptide 58 |
| 1420915_at | 9.16 | 0.00000 | STAT1 | signal transducer and activator of transcription 1, 91kDa |
| 1421550_a_at | 9.00 | 0.00000 | TRIM34 | tripartite motif containing 34 |
| 1435454_a_at | 9.00 | 0.00000 | PRIC285 | peroxisomal proliferator-activated receptor A interacting complex 285 |
| 1436538_at | 8.95 | 0.07195 | ANKRD37 | ankyrin repeat domain 37 |
| 1416897_at | 8.89 | 0.00000 | PARP9 | poly (ADP-ribose) polymerase family, member 9 |
| 1416016_at | 8.84 | 0.00000 | TAP1 | transporter 1, ATP-binding cassette, sub-family B (MDR/TAP) |
| 1424857_a_at | 8.68 | 0.00000 | TRIM34 | tripartite motif containing 34 |
| 1416380_at | 8.15 | 0.07195 | MOV10 | Mov10, Moloney leukemia virus 10, homolog (mouse) |
| 1443698_at | 8.10 | 0.00000 | XAF1 | XIAP associated factor 1 |
| 1424617_at | 8.03 | 0.00000 | IFI35 | interferon-induced protein 35 |
| 1445897_s_at | 7.94 | 0.00000 | IFI35 | interferon-induced protein 35 |
| 1455930_at | 7.85 | 0.00000 |  |  |
| 1459151_x_at | 7.73 | 0.00000 | IFI35 | interferon-induced protein 35 |
| 1455500_at | 7.53 | 0.00000 | RNF213 | ring finger protein 213 |
| 1417289_at | 7.43 | 0.00000 | PLEKHA2 | pleckstrin homology domain containing, family A (phosphoinositide binding specific) member 2 |
| 1428660_s_at | 7.30 | 0.00000 | TOR3A | torsin family 3, member A |
| 1450403_at | 7.16 | 0.00000 | STAT2 | signal transducer and activator of transcription 2, 113kDa |
| 1450696_at | 7.12 | 0.07195 | PSMB9 | proteasome (prosome, macropain) subunit, beta type, 9 (large multifunctional peptidase 2) |
| 1453840_at | 6.92 | 0.00000 | PABPC1 | poly(A) binding protein, cytoplasmic 1 |
| 1423062_at | 6.70 | 0.00000 | IGFBP3 | insulin-like growth factor binding protein 3 |
| 1416203_at | 6.66 | 0.00000 | AQP1 | aquaporin 1 (Colton blood group) |
| 1435465_at | 6.64 | 0.00000 | KBTBD11 | kelch repeat and BTB (POZ) domain containing 11 |
| 1418825_at | 6.56 | 0.00000 | IRGM | immunity-related GTPase family, M |
| 1427511_at | 6.47 | 0.07195 |  |  |
| 1426092_a_at | 6.39 | 0.00000 | TRIM34 | tripartite motif containing 34 |
| 1426774_at | 6.31 | 0.00000 | PARP12 | poly (ADP-ribose) polymerase family, member 12 |
| 1441727_s_at | 6.15 | 0.00000 | ZNF467 | zinc finger protein 467 |
| 1460603_at | 6.13 | 0.00000 | SAMD9L | sterile alpha motif domain containing 9-like |
| 1428909_at | 6.12 | 0.00000 | A130040M12Rik | RIKEN cDNA A130040M12 gene |
| 1452178_at | 6.10 | 0.00000 | PLEC | plectin |
| 1422962_a_at | 6.09 | 0.00000 | PSMB8 | proteasome (prosome, macropain) subunit, beta type, 8 (large multifunctional peptidase 7) |
| 1436172_at | 6.04 | 0.00000 | 9530028C05 | hypothetical protein 9530028C05 |
| 1421998_at | 5.98 | 0.00000 | TOR3A | torsin family 3, member A |
| 1455840_at | 5.94 | 0.07195 | RAPGEF5 | Rap guanine nucleotide exchange factor (GEF) 5 |
| 1425025_at | 5.79 | 0.00000 | TMEM106A | transmembrane protein 106A |
| 1435290_x_at | 5.71 | 0.07195 | HLA-DQA1 | major histocompatibility complex, class II, DQ alpha 1 |
| 1447016_at | 5.70 | 0.07195 | TBC1D1 | TBC1 (tre-2/USP6, BUB2, cdc16) domain family, member 1 |
| 1435945_a_at | 5.48 | 0.00000 | KCNN4 | potassium intermediate/small conductance calcium-activated channel, subfamily N, member 4 |
| 1439839_at | 5.40 | 0.00000 | D130051D11Rik | RIKEN cDNA D130051D11 gene |
| 1449875_s_at | 5.32 | 0.00000 | H2-T10/H2-T22 | histocompatibility 2, T region locus 10 |
| 1424775_at | 5.25 | 0.00000 | OAS1 | 2',5'-oligoadenylate synthetase 1, 40/46kDa |
| 1443339_at | 5.21 | 0.00000 | FAM162A | family with sequence similarity 162, member A |
| 1440717_at | 5.15 | 0.07195 | AA407881 | expressed sequence AA407881 |
| 1425396_a_at | 5.03 | 0.07195 | LCK | lymphocyte-specific protein tyrosine kinase |
| 1453757_at | 5.00 | 0.00000 | HERC6 | hect domain and RLD 6 |
| 1435208_at | 4.96 | 0.00000 | DTX3L | deltex 3-like (Drosophila) |
| 1422782_s_at | 4.96 | 0.00000 | TLR3 | toll-like receptor 3 |
| 1441631_at | 4.94 | 0.07195 | DDX24 | DEAD (Asp-Glu-Ala-Asp) box polypeptide 24 |
| 1451674_at | 4.92 | 0.07195 | SLC12A5 | solute carrier family 12 (potassium/chloride transporter), member 5 |
| 1431095_a_at | 4.91 | 0.00000 | HERC6 | hect domain and RLD 6 |
| 1423584_at | 4.89 | 0.07195 | IGFBP7 | insulin-like growth factor binding protein 7 |
| 1450454_at | 4.86 | 0.00000 | TOR3A | torsin family 3, member A |
| 1440028_at | 4.81 | 0.00000 | 4631423B10Rik | RIKEN cDNA 4631423B10 gene |
| 1438037_at | 4.80 | 0.00000 | HERC6 | hect domain and RLD 6 |
| 1456547_at | 4.75 | 0.07195 |  |  |
| 1446330_at | 4.69 | 0.07195 |  |  |
| 1456005_a_at | 4.68 | 0.07195 | BCL2L11 | BCL2-like 11 (apoptosis facilitator) |
| 1455581_x_at | 4.60 | 0.00000 | 9530028C05 | hypothetical protein 9530028C05 |
| 1432026_a_at | 4.59 | 0.00000 | HERC6 | hect domain and RLD 6 |
| 1422781_at | 4.55 | 0.07195 | TLR3 | toll-like receptor 3 |
| 1430362_at | 4.53 | 0.00000 | 5730409N24Rik | RIKEN cDNA 5730409N24 gene |
| 1445940_at | 4.51 | 0.07195 |  |  |
| 1421358_at | 4.46 | 0.00000 | HLA-G | major histocompatibility complex, class I, G |
| 1439276_at | 4.45 | 0.07195 | ADAR | adenosine deaminase, RNA-specific |
| 1459678_at | 4.45 | 0.07195 |  |  |
| 1448380_at | 4.43 | 0.00000 | LGALS3BP | lectin, galactoside-binding, soluble, 3 binding protein |
| 1417288_at | 4.42 | 0.00000 | PLEKHA2 | pleckstrin homology domain containing, family A (phosphoinositide binding specific) member 2 |
| 1447294_at | 4.41 | 0.00000 | FBXW11 | F-box and WD repeat domain containing 11 |
| 1425519_a_at | 4.40 | 0.07195 | CD74 | CD74 molecule, major histocompatibility complex, class II invariant chain |
| 1448940_at | 4.36 | 0.00000 | TRIM21 | tripartite motif containing 21 |
| 1459677_at | 4.35 | 0.07195 |  |  |
| 1459485_at | 4.31 | 0.00000 |  |  |
| 1458617_at | 4.30 | 0.07195 | ZMYND8 | zinc finger, MYND-type containing 8 |
| 1441593_at | 4.29 | 0.00000 |  |  |
| 1426971_at | 4.26 | 0.00000 | UBA7 | ubiquitin-like modifier activating enzyme 7 |
| 1446972_at | 4.21 | 0.07195 |  |  |
| 1454984_at | 4.20 | 0.07195 | LIFR | leukemia inhibitory factor receptor alpha |
| 1417023_a_at | 4.18 | 0.00000 | FABP4 | fatty acid binding protein 4, adipocyte |
| 1443675_at | 4.15 | 0.07195 |  |  |
| 1422005_at | 4.13 | 0.00000 | EIF2AK2 | eukaryotic translation initiation factor 2-alpha kinase 2 |
| 1435529_at | 4.13 | 0.00000 | IFIT1 | interferon-induced protein with tetratricopeptide repeats 1 |
| 1459845_at | 4.11 | 0.00000 |  |  |
| 1418174_at | 4.11 | 0.07195 | DBP | D site of albumin promoter (albumin D-box) binding protein |
| 1417149_at | 4.09 | 0.00000 | P4HA2 | prolyl 4-hydroxylase, alpha polypeptide II |
| 1441274_at | 4.08 | 0.07195 |  |  |
| 1433699_at | 4.07 | 0.07195 | TNFAIP3 | tumor necrosis factor, alpha-induced protein 3 |
| 1440341_at | 4.05 | 0.00000 |  |  |
| 1436457_at | 4.03 | 0.07195 | UBE2I | ubiquitin-conjugating enzyme E2I (UBC9 homolog, yeast) |
| 1417466_at | 4.01 | 0.00000 | RGS5 | regulator of G-protein signaling 5 |
| 1445827_at | 4.00 | 0.07195 | ZMYND8 | zinc finger, MYND-type containing 8 |
| 1421217_a_at | 4.00 | 0.00000 | LGALS9B | lectin, galactoside-binding, soluble, 9B |
| 1438257_at | 3.98 | 0.07195 |  |  |
| 1421322_a_at | 3.97 | 0.00000 | IRF9 | interferon regulatory factor 9 |
| 1429570_at | 3.91 | 0.00000 | MLKL | mixed lineage kinase domain-like |
| AFFX-18SRNAMur/X00686_5_at | 3.90 | 0.00000 | Rn18s | 18S ribosomal RNA |
| 1442925_at | 3.84 | 0.07195 | SMG1 | SMG1 homolog, phosphatidylinositol 3-kinase-related kinase (C. elegans) |
| 1429060_at | 3.82 | 0.00000 | MALAT1 | metastasis associated lung adenocarcinoma transcript 1 (non-protein coding) |
| 1421038_a_at | 3.81 | 0.00000 | KCNN4 | potassium intermediate/small conductance calcium-activated channel, subfamily N, member 4 |
| 1441536_at | 3.77 | 0.07195 | HMGCS1 | 3-hydroxy-3-methylglutaryl-CoA synthase 1 (soluble) |
| 1457847_at | 3.76 | 0.00000 |  |  |
| 1441769_at | 3.75 | 0.07195 |  |  |
| 1422640_at | 3.74 | 0.07195 | Pcdhb9 | protocadherin beta 9 |
| 1427285_s_at | 3.73 | 0.07195 | MALAT1 | metastasis associated lung adenocarcinoma transcript 1 (non-protein coding) |
| 1439087_a_at | 3.72 | 0.07195 | PIK3IP1 | phosphoinositide-3-kinase interacting protein 1 |
| 1439825_at | 3.71 | 0.07195 | DTX3L | deltex 3-like (Drosophila) |
| 1430932_at | 3.71 | 0.07195 | SLC9A8 | solute carrier family 9 (sodium/hydrogen exchanger), member 8 |
| 1442625_at | 3.70 | 0.07195 |  |  |
| 1458257_at | 3.70 | 0.07195 |  |  |
| 1444194_at | 3.69 | 0.07195 |  |  |
| 1416997_a_at | 3.69 | 0.00000 | HAP1 | huntingtin-associated protein 1 |
| 1416811_s_at | 3.66 | 0.07195 | Ctla2a | cytotoxic T lymphocyte-associated protein 2 alpha |
| 1457550_at | 3.64 | 0.00000 | 9530059O14Rik | RIKEN cDNA 9530059O14 gene |
| 1436936_s_at | 3.64 | 0.07195 | XIST | X (inactive)-specific transcript (non-protein coding) |
| 1420098_s_at | 3.63 | 0.00000 | D13Ertd787e | DNA segment, Chr 13, ERATO Doi 787, expressed |
| 1457019_s_at | 3.60 | 0.07195 | RDH14 | retinol dehydrogenase 14 (all-trans/9-cis/11-cis) |
| 1425719_a_at | 3.59 | 0.00000 | NMI | N-myc (and STAT) interactor |
| 1457806_at | 3.59 | 0.00000 | FAM196A | family with sequence similarity 196, member A |
| 1451263_a_at | 3.57 | 0.00000 | FABP4 | fatty acid binding protein 4, adipocyte |
| 1437432_a_at | 3.54 | 0.07195 | Trim12a | tripartite motif-containing 12A |
| 1444599_at | 3.53 | 0.07195 | HERC4 | hect domain and RLD 4 |
| 1418930_at | 3.53 | 0.00000 | CXCL10 | chemokine (C-X-C motif) ligand 10 |
| 1436698_x_at | 3.52 | 0.00000 | TMEM204 | transmembrane protein 204 |
| 1437689_x_at | 3.52 | 0.07195 | CLU | clusterin |
| 1438127_at | 3.50 | 0.07195 |  |  |
| 1418483_a_at | 3.50 | 0.07195 | GGTA1 | glycoprotein, alpha-galactosyltransferase 1 pseudogene |
| 1440163_at | 3.49 | 0.07195 | SETD3 | SET domain containing 3 |
| 1417185_at | 3.49 | 0.07195 | Ly6a | lymphocyte antigen 6 complex, locus A |
| 1447408_at | 3.49 | 0.07195 |  |  |
| 1418077_at | 3.47 | 0.07195 | TRIM21 | tripartite motif containing 21 |
| 1433242_at | 3.46 | 0.07195 | 5830415B17Rik | RIKEN cDNA 5830415B17 gene |
| 1459649_at | 3.46 | 0.07195 |  |  |
| 1449254_at | 3.45 | 0.07195 | SPP1 | secreted phosphoprotein 1 |
| 1441558_at | 3.44 | 0.00000 | D230044B12Rik | RIKEN cDNA D230044B12 gene |
| 1438033_at | 3.43 | 0.07195 | TEF | thyrotrophic embryonic factor |
| 1425405_a_at | 3.43 | 0.00000 | ADAR | adenosine deaminase, RNA-specific |
| 1456014_s_at | 3.43 | 0.07195 | FERMT3 | fermitin family member 3 |
| 1428332_at | 3.42 | 0.07195 | PIK3IP1 | phosphoinositide-3-kinase interacting protein 1 |
| 1458556_at | 3.42 | 0.07195 |  |  |
| 1453864_at | 3.42 | 0.00000 | RDH14 | retinol dehydrogenase 14 (all-trans/9-cis/11-cis) |
| 1447258_at | 3.42 | 0.07195 |  |  |
| 1447706_at | 3.41 | 0.07195 |  |  |
| 1424375_s_at | 3.39 | 0.00000 | GIMAP4 | GTPase, IMAP family member 4 |
| 1430622_at | 3.39 | 0.07195 | 4833423F13Rik | RIKEN cDNA 4833423F13 gene |
| 1437062_s_at | 3.39 | 0.00000 | PHYHIPL | phytanoyl-CoA 2-hydroxylase interacting protein-like |
| 1448323_a_at | 3.37 | 0.07195 | BGN | biglycan |
| 1443109_at | 3.37 | 0.07195 |  |  |
| 1457304_at | 3.35 | 0.07195 | D13Ertd787e | DNA segment, Chr 13, ERATO Doi 787, expressed |
| 1443353_at | 3.34 | 0.07195 |  |  |
| 1444228_s_at | 3.34 | 0.07195 | HERC2 | hect domain and RLD 2 |
| 1424051_at | 3.34 | 0.00000 | COL4A2 | collagen, type IV, alpha 2 |
| 1444349_at | 3.33 | 0.00000 |  |  |
| 1456808_at | 3.33 | 0.07195 |  |  |
| 1439948_at | 3.32 | 0.00000 | BC046401 | cDNA sequence BC046401 |
| 1430058_at | 3.32 | 0.07195 | SLBP | stem-loop binding protein |
| 1426210_x_at | 3.30 | 0.07195 | PARP3 | poly (ADP-ribose) polymerase family, member 3 |
| 1438009_at | 3.30 | 0.07195 | HIST1H2AB/HIST1H2AE | histone cluster 1, H2ae |
| 1448632_at | 3.29 | 0.00000 | PSMB10 | proteasome (prosome, macropain) subunit, beta type, 10 |
| 1444498_at | 3.29 | 0.07195 | GPHN | gephyrin |
| 1443923_at | 3.28 | 0.07195 | AKAP13 | A kinase (PRKA) anchor protein 13 |
| 1446475_at | 3.28 | 0.07195 |  |  |
| 1438338_at | 3.26 | 0.07195 | MDH1 | malate dehydrogenase 1, NAD (soluble) |
| 1423679_at | 3.26 | 0.07195 | C9orf125 | chromosome 9 open reading frame 125 |
| 1457626_at | 3.25 | 0.00000 | D3Wsu106e | DNA segment, Chr 3, Wayne State University 106, expressed |
| 1442338_at | 3.25 | 0.07195 |  |  |
| 1448590_at | 3.25 | 0.07195 | COL6A1 | collagen, type VI, alpha 1 |
| 1458721_at | 3.24 | 0.00000 |  |  |
| 1460032_at | 3.24 | 0.07195 |  |  |
| 1415806_at | 3.23 | 0.07195 | PLAT | plasminogen activator, tissue |
| 1418536_at | 3.23 | 0.00000 | HLA-B | major histocompatibility complex, class I, B |
| 1458038_at | 3.22 | 0.00000 |  |  |
| 1458050_at | 3.21 | 0.00000 |  |  |
| 1422155_at | 3.21 | 0.07195 | HIST1H3A | histone cluster 1, H3a |
| 1419473_a_at | 3.21 | 0.07195 | CCK | cholecystokinin |
| 1426716_at | 3.21 | 0.00000 | TDRD7 | tudor domain containing 7 |
| 1435477_s_at | 3.20 | 0.00000 | FCGR2B | Fc fragment of IgG, low affinity IIb, receptor (CD32) |
| 1446939_at | 3.20 | 0.07195 | Trim12a | tripartite motif-containing 12A |
| 1443088_at | 3.19 | 0.07195 | 9930031P18Rik | RIKEN cDNA 9930031P18 gene |
| 1436363_a_at | 3.19 | 0.07195 | NFIX | nuclear factor I/X (CCAAT-binding transcription factor) |
| 1424354_at | 3.19 | 0.07195 | TMEM140 | transmembrane protein 140 |
| 1439908_at | 3.17 | 0.00000 | ZKSCAN1 | zinc finger with KRAB and SCAN domains 1 |
| 1440578_at | 3.17 | 0.07195 |  |  |
| 1459463_at | 3.17 | 0.07195 |  |  |
| 1448416_at | 3.16 | 0.00000 | MGP | matrix Gla protein |
| 1444598_at | 3.16 | 0.07195 |  |  |
| 1433110_at | 3.16 | 0.07195 | 5830474E16Rik | RIKEN cDNA 5830474E16 gene |
| 1441843_s_at | 3.16 | 0.00000 | 5230400M03Rik | RIKEN cDNA 5230400M03 gene |
| 1441779_at | 3.15 | 0.00000 | 9530006C21Rik | RIKEN cDNA 9530006C21 gene |
| 1416405_at | 3.15 | 0.00000 | BGN | biglycan |
| 1447984_at | 3.15 | 0.07195 |  |  |
| 1440295_at | 3.15 | 0.00000 | C2orf43 | chromosome 2 open reading frame 43 |
| 1459497_at | 3.15 | 0.07195 |  |  |
| 1456661_at | 3.14 | 0.07195 |  |  |
| 1444126_at | 3.11 | 0.07195 |  |  |
| 1442494_at | 3.11 | 0.07195 | C79242 | expressed sequence C79242 |
| 1444507_at | 3.10 | 0.07195 | USP53 | ubiquitin specific peptidase 53 |
| 1418090_at | 3.09 | 0.00000 | PLVAP | plasmalemma vesicle associated protein |
| 1452378_at | 3.09 | 0.07195 | MALAT1 | metastasis associated lung adenocarcinoma transcript 1 (non-protein coding) |
| 1441460_at | 3.09 | 0.00000 |  |  |
| 1446802_at | 3.09 | 0.07195 | ATOX1 | ATX1 antioxidant protein 1 homolog (yeast) |
| 1447416_at | 3.09 | 0.07195 | C12orf28 | chromosome 12 open reading frame 28 |
| 1458296_at | 3.08 | 0.07195 |  |  |
| 1434438_at | 3.07 | 0.00000 | SAMHD1 | SAM domain and HD domain 1 |
| 1416221_at | 3.07 | 0.07195 | FSTL1 | follistatin-like 1 |
| 1456717_at | 3.07 | 0.07195 |  |  |
| 1452477_at | 3.06 | 0.07195 | C4orf29 | chromosome 4 open reading frame 29 |
| 1456960_at | 3.05 | 0.07195 |  |  |
| 1460302_at | 3.05 | 0.07195 | THBS1 | thrombospondin 1 |
| 1424921_at | 3.05 | 0.07195 | BST2 | bone marrow stromal cell antigen 2 |
| 1446223_at | 3.05 | 0.07195 |  |  |
| 1428306_at | 3.05 | 0.00000 | DDIT4 | DNA-damage-inducible transcript 4 |
| 1439650_at | 3.03 | 0.00000 | RTN4 | reticulon 4 |
| 1460330_at | 3.03 | 0.00000 | ANXA3 | annexin A3 |
| 1457458_at | 3.03 | 0.00000 | ZC3H4 | zinc finger CCCH-type containing 4 |
| 1457934_at | 3.02 | 0.07195 |  |  |
| 1443020_at | 3.02 | 0.07195 | HMBOX1 | homeobox containing 1 |
| 1457559_at | 3.01 | 0.07195 |  |  |
| 1429722_at | 3.01 | 0.07195 | ZBTB4 | zinc finger and BTB domain containing 4 |
| 1459917_at | 3.00 | 0.07195 | GGNBP2 | gametogenetin binding protein 2 |
| 1430412_at | 3.00 | 0.07195 | 2310068C19Rik | RIKEN cDNA 2310068C19 gene |
| 1437868_at | 3.00 | 0.00000 | FAM46A | family with sequence similarity 46, member A |
| 1448076_at | 2.98 | 0.07195 | CTR9 | Ctr9, Paf1/RNA polymerase II complex component, homolog (S. cerevisiae) |
| 1459143_at | 2.98 | 0.00000 | CHCHD3 | coiled-coil-helix-coiled-coil-helix domain containing 3 |
| 1435198_at | 2.97 | 0.07195 | Gm5817 | predicted gene 5817 |
| 1427091_at | 2.97 | 0.00000 | ZNFX1 | zinc finger, NFX1-type containing 1 |
| 1441231_at | 2.97 | 0.07195 | Gm3626 | predicted gene 3626 |
| 1418187_at | 2.95 | 0.07195 | RAMP2 | receptor (G protein-coupled) activity modifying protein 2 |
| 1443513_at | 2.95 | 0.00000 |  |  |
| 1447147_at | 2.94 | 0.07195 |  |  |
| 1457794_at | 2.93 | 0.07195 | WHSC1L1 | Wolf-Hirschhorn syndrome candidate 1-like 1 |
| 1458947_at | 2.93 | 0.07195 |  |  |
| 1450672_a_at | 2.93 | 0.00000 | TREX1 | three prime repair exonuclease 1 |
| 1448289_at | 2.92 | 0.00000 | CRMP1 | collapsin response mediator protein 1 |
| 1440513_at | 2.92 | 0.07195 | C80258 | expressed sequence C80258 |
| 1451644_a_at | 2.92 | 0.07195 | H2-Gs10 | MHC class I like protein GS10 |
| 1443956_at | 2.92 | 0.07195 | ZNF397 | zinc finger protein 397 |
| 1454532_at | 2.92 | 0.07195 | C030043A13Rik | RIKEN cDNA C030043A13 gene |
| 1439946_at | 2.92 | 0.07195 |  |  |
| 1416740_at | 2.92 | 0.07195 | COL5A1 | collagen, type V, alpha 1 |
| 1454849_x_at | 2.91 | 0.07195 | CLU | clusterin |
| 1448259_at | 2.91 | 0.07195 | FSTL1 | follistatin-like 1 |
| 1445387_at | 2.91 | 0.00000 | SENP6 | SUMO1/sentrin specific peptidase 6 |
| 1458661_at | 2.90 | 0.07195 |  |  |
| 1459154_at | 2.90 | 0.07195 |  |  |
| 1449556_at | 2.90 | 0.07195 | HLA-E | major histocompatibility complex, class I, E |
| 1441855_x_at | 2.89 | 0.07195 | CXCL2 | chemokine (C-X-C motif) ligand 2 |
| 1429921_at | 2.88 | 0.07195 | C5orf15 | chromosome 5 open reading frame 15 |
| 1446938_at | 2.87 | 0.07195 | AA408213 | expressed sequence AA408213 |
| 1440892_at | 2.87 | 0.07195 |  |  |
| 1431528_at | 2.85 | 0.07195 | 5830427D02Rik | RIKEN cDNA 5830427D02 gene |
| 1444070_at | 2.83 | 0.07195 | AA914427 | EST AA914427 |
| 1429701_at | 2.83 | 0.00000 | MAGEB16 | melanoma antigen family B, 16 |
| 1423754_at | 2.83 | 0.00000 | IFITM3 | interferon induced transmembrane protein 3 (1-8U) |
| 1456277_at | 2.83 | 0.07195 | 7530414M10Rik | RIKEN cDNA 7530414M10 gene |
| 1445116_at | 2.83 | 0.07195 |  |  |
| 1415978_at | 2.82 | 0.00000 | TUBB3 | tubulin, beta 3 |
| 1444785_at | 2.81 | 0.07195 |  |  |
| 1441352_at | 2.81 | 0.07195 | 9430034N14Rik | RIKEN cDNA 9430034N14 gene |
| 1441896_x_at | 2.81 | 0.07195 |  |  |
| 1431316_at | 2.81 | 0.07195 | ITCH | itchy E3 ubiquitin protein ligase homolog (mouse) |
| 1441858_at | 2.80 | 0.07195 |  |  |
| 1419706_a_at | 2.80 | 0.07195 | AKAP12 | A kinase (PRKA) anchor protein 12 |
| 1442238_a_at | 2.80 | 0.07195 | KIF6 | kinesin family member 6 |
| 1416454_s_at | 2.79 | 0.00000 | ACTG2 | actin, gamma 2, smooth muscle, enteric |
| 1451969_s_at | 2.79 | 0.00000 | PARP3 | poly (ADP-ribose) polymerase family, member 3 |
| 1453791_at | 2.78 | 0.07195 | C130071C03Rik | RIKEN cDNA C130071C03 gene |
| 1430608_at | 2.78 | 0.07195 | 4930535I16Rik | RIKEN cDNA 4930535I16 gene |
| 1431381_at | 2.78 | 0.07195 | 3110005L24Rik | RIKEN cDNA 3110005L24 gene |
| 1457390_at | 2.77 | 0.07195 |  |  |
| 1419647_a_at | 2.75 | 0.00000 | IER3 | immediate early response 3 |
| 1442609_at | 2.74 | 0.07195 |  |  |
| 1436197_at | 2.74 | 0.07195 | CDC42BPG | CDC42 binding protein kinase gamma (DMPK-like) |
| 1442269_at | 2.73 | 0.07195 |  |  |
| 1454463_at | 2.73 | 0.07195 | 5430402O13Rik | RIKEN cDNA 5430402O13 gene |
| 1440083_at | 2.73 | 0.07195 | A430061O12Rik | RIKEN cDNA A430061O12 gene |
| 1457404_at | 2.73 | 0.07195 | NFKBIZ | nuclear factor of kappa light polypeptide gene enhancer in B-cells inhibitor, zeta |
| 1444886_at | 2.72 | 0.07195 | APPBP2 | amyloid beta precursor protein (cytoplasmic tail) binding protein 2 |
| 1430585_at | 2.72 | 0.07195 | 5930436O19Rik | RIKEN cDNA 5930436O19 gene |
| 1433741_at | 2.71 | 0.07195 | CD38 | CD38 molecule |
| 1439952_at | 2.71 | 0.07195 | DDX23 | DEAD (Asp-Glu-Ala-Asp) box polypeptide 23 |
| 1458299_s_at | 2.71 | 0.07195 | NFKBIE | nuclear factor of kappa light polypeptide gene enhancer in B-cells inhibitor, epsilon |
| 1458305_at | 2.70 | 0.07195 | TMTC3 | transmembrane and tetratricopeptide repeat containing 3 |
| 1448914_a_at | 2.69 | 0.00000 | CSF1 | colony stimulating factor 1 (macrophage) |
| 1448908_at | 2.69 | 0.07195 | PPAP2B | phosphatidic acid phosphatase type 2B |
| 1442251_at | 2.68 | 0.07195 | VCPIP1 | valosin containing protein (p97)/p47 complex interacting protein 1 |
| 1440068_at | 2.68 | 0.07195 |  |  |
| 1416942_at | 2.68 | 0.07195 | ERAP1 | endoplasmic reticulum aminopeptidase 1 |
| 1441006_at | 2.68 | 0.07195 | DUS4L | dihydrouridine synthase 4-like (S. cerevisiae) |
| 1431060_at | 2.67 | 0.07195 | PELI1 | pellino homolog 1 (Drosophila) |
| 1443547_at | 2.66 | 0.07195 |  |  |
| 1459887_at | 2.65 | 0.07195 |  |  |
| 1444128_at | 2.64 | 0.07195 | ARHGAP26 | Rho GTPase activating protein 26 |
| 1448649_at | 2.64 | 0.07195 | ENPEP | glutamyl aminopeptidase (aminopeptidase A) |
| 1454933_at | 2.64 | 0.07195 | FAM176B | family with sequence similarity 176, member B |
| 1423586_at | 2.64 | 0.07195 | AXL | AXL receptor tyrosine kinase |
| 1418131_at | 2.63 | 0.07195 | SAMHD1 | SAM domain and HD domain 1 |
| 1445888_x_at | 2.63 | 0.07195 | PARP3 | poly (ADP-ribose) polymerase family, member 3 |
| 1447526_at | 2.62 | 0.07195 | D5Ertd255e | DNA segment, Chr 5, ERATO Doi 255, expressed |
| 1457166_at | 2.62 | 0.07195 |  |  |
| 1445427_at | 2.60 | 0.07195 |  |  |
| 1455265_a_at | 2.60 | 0.07195 | RGS16 | regulator of G-protein signaling 16 |
| 1436851_at | 2.60 | 0.07195 | PTGER1 | prostaglandin E receptor 1 (subtype EP1), 42kDa |
| 1440866_at | 2.60 | 0.07195 | EIF2AK2 | eukaryotic translation initiation factor 2-alpha kinase 2 |
| 1442358_at | 2.59 | 0.07195 | AA409587 | expressed sequence AA409587 |
| 1437503_a_at | 2.59 | 0.00000 | SHISA5 | shisa homolog 5 (Xenopus laevis) |
| 1448757_at | 2.59 | 0.07195 | PML | promyelocytic leukemia |
| 1457477_at | 2.59 | 0.07195 | MBNL2 | muscleblind-like 2 (Drosophila) |
| 1438890_at | 2.59 | 0.07195 | KDM2A | lysine (K)-specific demethylase 2A |
| 1444728_at | 2.59 | 0.07195 |  |  |
| 1439348_at | 2.58 | 0.07195 | S100A10 | S100 calcium binding protein A10 |
| 1438929_at | 2.58 | 0.07195 |  |  |
| 1457508_at | 2.57 | 0.07195 | C430003N24Rik | RIKEN cDNA C430003N24 gene |
| 1458282_at | 2.57 | 0.07195 |  |  |
| 1439626_at | 2.57 | 0.07195 | TMOD3 | tropomodulin 3 (ubiquitous) |
| 1460220_a_at | 2.56 | 0.07195 | CSF1 | colony stimulating factor 1 (macrophage) |
| 1423986_a_at | 2.56 | 0.07195 | SHISA5 | shisa homolog 5 (Xenopus laevis) |
| 1441397_at | 2.56 | 0.07195 |  |  |
| 1447901_x_at | 2.55 | 0.07195 | SFI1 | Sfi1 homolog, spindle assembly associated (yeast) |
| 1420342_at | 2.54 | 0.07195 | Gdap10 | ganglioside-induced differentiation-associated-protein 10 |
| 1421811_at | 2.54 | 0.07195 | THBS1 | thrombospondin 1 |
| 1442295_at | 2.54 | 0.07195 | ARPC2 | actin related protein 2/3 complex, subunit 2, 34kDa |
| 1442760_x_at | 2.54 | 0.07195 |  |  |
| 1443689_at | 2.53 | 0.07195 |  |  |
| 1458469_at | 2.53 | 0.07195 | CBLB | Cas-Br-M (murine) ecotropic retroviral transforming sequence b |
| 1416488_at | 2.52 | 0.07195 | CCNG2 | cyclin G2 |
| 1448562_at | 2.52 | 0.07195 | UPP1 | uridine phosphorylase 1 |
| 1456868_at | 2.52 | 0.07195 |  |  |
| 1457357_at | 2.52 | 0.07195 | TLK2 | tousled-like kinase 2 |
| 1441415_at | 2.52 | 0.07195 |  |  |
| 1443083_at | 2.51 | 0.07195 |  |  |
| 1441293_at | 2.51 | 0.00000 |  |  |
| 1447691_x_at | 2.50 | 0.07195 |  |  |
| 1433501_at | 2.50 | 0.07195 | CTSO | cathepsin O |
| 1456358_at | 2.50 | 0.07195 | ETV3 | ets variant 3 |
| 1430792_at | 2.50 | 0.07195 |  |  |
| 1440457_at | 2.49 | 0.07195 |  |  |
| 1458179_at | 2.49 | 0.07195 |  |  |
| 1445759_at | 2.49 | 0.07195 |  |  |
| 1456821_at | 2.49 | 0.07195 |  |  |
| 1458376_at | 2.49 | 0.07195 | B930025B16Rik | RIKEN cDNA B930025B16 gene |
| 1448436_a_at | 2.49 | 0.00000 | IRF1 | interferon regulatory factor 1 |
| 1444378_at | 2.49 | 0.07195 |  |  |
| 1442168_at | 2.48 | 0.07195 | A230071A22Rik | RIKEN cDNA A230071A22 gene |
| 1417061_at | 2.48 | 0.07195 | SLC40A1 | solute carrier family 40 (iron-regulated transporter), member 1 |
| 1456933_at | 2.48 | 0.07195 |  |  |
| 1439652_at | 2.48 | 0.07195 |  |  |
| 1457233_at | 2.47 | 0.07195 | DNAJA2 | DnaJ (Hsp40) homolog, subfamily A, member 2 |
| 1441531_at | 2.47 | 0.07195 | PLCB4 | phospholipase C, beta 4 |
| 1440260_at | 2.46 | 0.07195 | CCDC58 | coiled-coil domain containing 58 |
| 1458134_at | 2.46 | 0.07195 |  |  |
| 1440925_at | 2.46 | 0.07195 |  |  |
| 1427167_at | 2.46 | 0.07195 | ARMCX4 | armadillo repeat containing, X-linked 4 |
| 1447312_at | 2.46 | 0.07195 |  |  |
| 1457551_at | 2.45 | 0.07195 |  |  |
| 1444764_at | 2.45 | 0.07195 |  |  |
| 1443702_at | 2.45 | 0.07195 | MAP4 | microtubule-associated protein 4 |
| 1456103_at | 2.45 | 0.07195 | PML | promyelocytic leukemia |
| 1452500_at | 2.45 | 0.07195 |  |  |
| 1424131_at | 2.45 | 0.07195 | COL6A3 | collagen, type VI, alpha 3 |
| 1425573_a_at | 2.45 | 0.07195 | ASAP1 | ArfGAP with SH3 domain, ankyrin repeat and PH domain 1 |
| 1447491_at | 2.44 | 0.07195 |  |  |
| 1444677_at | 2.44 | 0.07195 | C77673 | expressed sequence C77673 |
| 1447830_s_at | 2.44 | 0.07195 | RGS2 | regulator of G-protein signaling 2, 24kDa |
| 1442775_at | 2.44 | 0.07195 |  |  |
| 1439414_x_at | 2.44 | 0.07195 |  |  |
| 1448793_a_at | 2.44 | 0.07195 | SDC4 | syndecan 4 |
| 1439038_at | 2.43 | 0.07195 | KHNYN | KH and NYN domain containing |
| 1426037_a_at | 2.43 | 0.07195 | RGS16 | regulator of G-protein signaling 16 |
| 1439068_at | 2.43 | 0.07195 | ERAP1 | endoplasmic reticulum aminopeptidase 1 |
| 1431047_at | 2.42 | 0.07195 | CHD6 | chromodomain helicase DNA binding protein 6 |
| 1440594_at | 2.42 | 0.07195 |  |  |
| 1430529_at | 2.41 | 0.07195 | CSNK1A1 | casein kinase 1, alpha 1 |
| 1431347_at | 2.41 | 0.07195 | 5730407M17Rik | RIKEN cDNA 5730407M17 gene |
| 1445746_at | 2.41 | 0.07195 | EIF4H | eukaryotic translation initiation factor 4H |
| 1453266_at | 2.41 | 0.07195 | ZBTB4 | zinc finger and BTB domain containing 4 |
| 1441823_at | 2.41 | 0.07195 | ZMIZ1 | zinc finger, MIZ-type containing 1 |
| 1446731_at | 2.41 | 0.07195 | FANCF | Fanconi anemia, complementation group F |
| 1446245_at | 2.40 | 0.07195 |  |  |
| 1444034_at | 2.40 | 0.07195 |  |  |
| 1444037_at | 2.40 | 0.00000 | LMAN1 | lectin, mannose-binding, 1 |
| 1434773_a_at | 2.40 | 0.00000 | SLC2A1 | solute carrier family 2 (facilitated glucose transporter), member 1 |
| 1443390_at | 2.39 | 0.07195 |  |  |
| 1419435_at | 2.39 | 0.07195 | AOX1 | aldehyde oxidase 1 |
| 1420072_s_at | 2.39 | 0.07195 |  |  |
| 1438921_at | 2.39 | 0.07195 | ATR | ataxia telangiectasia and Rad3 related |
| 1422622_at | 2.39 | 0.07195 | NOS3 | nitric oxide synthase 3 (endothelial cell) |
| 1445708_x_at | 2.38 | 0.00000 | 3110021A11Rik | RIKEN cDNA 3110021A11 gene |
| 1436830_at | 2.38 | 0.07195 | MARVELD1 | MARVEL domain containing 1 |
| 1446512_at | 2.38 | 0.07195 | ZC3H15 | zinc finger CCCH-type containing 15 |
| 1446914_at | 2.38 | 0.07195 | C80425 | expressed sequence C80425 |
| 1457202_at | 2.38 | 0.07195 |  |  |
| 1441570_at | 2.38 | 0.07195 |  |  |
| 1444574_at | 2.37 | 0.07195 |  |  |
| 1428173_at | 2.37 | 0.07195 | EML2 | echinoderm microtubule associated protein like 2 |
| 1444044_at | 2.37 | 0.07195 |  |  |
| 1440183_x_at | 2.37 | 0.07195 | C14orf153 | chromosome 14 open reading frame 153 |
| 1456662_at | 2.37 | 0.07195 | AA386476 | expressed sequence AA386476 |
| 1418166_at | 2.36 | 0.07195 | IL12RB1 | interleukin 12 receptor, beta 1 |
| 1450402_at | 2.36 | 0.07195 | MED1 | mediator complex subunit 1 |
| 1457161_at | 2.36 | 0.07195 | 9530029O12Rik | RIKEN cDNA 9530029O12 gene |
| 1422006_at | 2.36 | 0.07195 | EIF2AK2 | eukaryotic translation initiation factor 2-alpha kinase 2 |
| 1445613_at | 2.36 | 0.07195 | 9430019H13Rik | RIKEN cDNA 9430019H13 gene |
| 1460188_at | 2.35 | 0.07195 | PTPN6 | protein tyrosine phosphatase, non-receptor type 6 |
| 1435605_at | 2.35 | 0.07195 | ACTR3B | ARP3 actin-related protein 3 homolog B (yeast) |
| 1443010_at | 2.35 | 0.07195 |  |  |
| 1444295_at | 2.35 | 0.07195 | NEO1 | neogenin 1 |
| 1439572_at | 2.35 | 0.07195 | R3HDM1 | R3H domain containing 1 |
| 1439664_at | 2.35 | 0.07195 | DCAF7 | DDB1 and CUL4 associated factor 7 |
| 1431843_a_at | 2.34 | 0.07195 | NFKBIE | nuclear factor of kappa light polypeptide gene enhancer in B-cells inhibitor, epsilon |
| 1440351_at | 2.34 | 0.07195 |  |  |
| 1459315_at | 2.34 | 0.07195 |  |  |
| 1440268_at | 2.34 | 0.07195 | TRIM41 | tripartite motif containing 41 |
| 1450541_at | 2.34 | 0.07195 | PVT1 | Pvt1 oncogene (non-protein coding) |
| 1422341_s_at | 2.34 | 0.07195 | PLA2G15 | phospholipase A2, group XV |
| 1431677_at | 2.34 | 0.07195 |  |  |
| 1438585_at | 2.34 | 0.07195 | SYNE2 | spectrin repeat containing, nuclear envelope 2 |
| 1442274_at | 2.33 | 0.07195 | ZDHHC15 | zinc finger, DHHC-type containing 15 |
| 1451832_at | 2.33 | 0.07195 | CKLF | chemokine-like factor |
| 1453824_at | 2.33 | 0.07195 | CCDC76 | coiled-coil domain containing 76 |
| 1436870_s_at | 2.33 | 0.07195 | AFAP1L2 | actin filament associated protein 1-like 2 |
| 1452388_at | 2.33 | 0.07195 | HSPA1A/HSPA1B | heat shock 70kDa protein 1A |
| 1427425_at | 2.32 | 0.07195 | MCART1 | mitochondrial carrier triple repeat 1 |
| 1429423_at | 2.32 | 0.07195 | 4930518I15Rik | RIKEN cDNA 4930518I15 gene |
| 1450387_s_at | 2.32 | 0.07195 | AK4 | adenylate kinase 4 |
| 1441108_at | 2.32 | 0.00000 |  |  |
| 1430220_at | 2.32 | 0.07195 | C5orf34 | chromosome 5 open reading frame 34 |
| 1443521_at | 2.31 | 0.07195 |  |  |
| 1436889_at | 2.31 | 0.07195 | GABRA1 | gamma-aminobutyric acid (GABA) A receptor, alpha 1 |
| 1457689_at | 2.31 | 0.07195 | SBF2 | SET binding factor 2 |
| 1435137_s_at | 2.30 | 0.07195 | A130040M12Rik | RIKEN cDNA A130040M12 gene |
| 1440551_at | 2.30 | 0.07195 |  |  |
| 1444557_at | 2.30 | 0.07195 |  |  |
| 1446118_at | 2.30 | 0.07195 |  |  |
| 1458811_at | 2.29 | 0.07195 | 9430047L24Rik | RIKEN cDNA 9430047L24 gene |
| 1438316_a_at | 2.29 | 0.07195 | CCDC102A | coiled-coil domain containing 102A |
| 1458708_at | 2.29 | 0.07195 |  |  |
| 1439293_at | 2.29 | 0.07195 | KIAA1370 | KIAA1370 |
| 1428814_at | 2.29 | 0.07195 |  |  |
| 1439655_at | 2.29 | 0.07195 |  |  |
| 1447064_at | 2.28 | 0.07195 |  |  |
| 1444828_at | 2.28 | 0.07195 |  |  |
| 1443489_at | 2.28 | 0.07195 |  |  |
| 1456272_at | 2.28 | 0.07195 |  |  |
| 1441172_at | 2.28 | 0.07195 |  |  |
| 1447143_at | 2.27 | 0.07195 |  |  |
| 1426604_at | 2.27 | 0.07195 | RNASEL | ribonuclease L (2',5'-oligoisoadenylate synthetase-dependent) |
| 1458345_s_at | 2.27 | 0.07195 | COLEC11 | collectin sub-family member 11 |
| 1444328_at | 2.27 | 0.00000 | CLTA | clathrin, light chain A |
| 1437676_at | 2.27 | 0.07195 | SPAG9 | sperm associated antigen 9 |
| 1443526_at | 2.26 | 0.07195 |  |  |
| 1459322_at | 2.26 | 0.07195 |  |  |
| 1459745_at | 2.26 | 0.07195 |  |  |
| 1455712_at | 2.26 | 0.07195 | HIST3H2A | histone cluster 3, H2a |
| 1457814_at | 2.26 | 0.07195 |  |  |
| 1457901_at | 2.26 | 0.07195 |  |  |
| 1454393_at | 2.25 | 0.07195 | DZIP3 | DAZ interacting protein 3, zinc finger |
| 1441789_at | 2.25 | 0.07195 |  |  |
| 1445646_at | 2.25 | 0.07195 |  |  |
| 1419879_s_at | 2.24 | 0.07195 | TRIM25 | tripartite motif containing 25 |
| 1443586_at | 2.24 | 0.00000 | FIP1L1 | FIP1 like 1 (S. cerevisiae) |
| 1436405_at | 2.24 | 0.07195 | DOCK4 | dedicator of cytokinesis 4 |
| 1437341_x_at | 2.24 | 0.00000 | CNP | 2',3'-cyclic nucleotide 3' phosphodiesterase |
| 1435284_at | 2.24 | 0.07195 | RTN4 | reticulon 4 |
| 1457313_at | 2.24 | 0.00000 | OCRL | oculocerebrorenal syndrome of Lowe |
| 1457338_at | 2.24 | 0.07195 |  |  |
| 1416342_at | 2.23 | 0.07195 | TNC | tenascin C |
| 1451082_at | 2.23 | 0.07195 | FTSJD2 | FtsJ methyltransferase domain containing 2 |
| 1440421_at | 2.23 | 0.07195 |  |  |
| 1455796_x_at | 2.23 | 0.07195 | OLFM1 | olfactomedin 1 |
| 1439261_x_at | 2.23 | 0.00000 | MITD1 | MIT, microtubule interacting and transport, domain containing 1 |
| 1437614_x_at | 2.23 | 0.07195 | ZDHHC14 | zinc finger, DHHC-type containing 14 |
| 1440847_at | 2.23 | 0.00000 | MTSS1 | metastasis suppressor 1 |
| 1420940_x_at | 2.23 | 0.00000 | RGS5 | regulator of G-protein signaling 5 |
| 1453627_at | 2.22 | 0.07195 | 2610020P09Rik | RIKEN cDNA 2610020P09 gene |
| 1439537_at | 2.22 | 0.07195 |  |  |
| 1443330_at | 2.22 | 0.07195 |  |  |
| 1443926_at | 2.22 | 0.07195 |  |  |
| 1426599_a_at | 2.22 | 0.00000 | SLC2A1 | solute carrier family 2 (facilitated glucose transporter), member 1 |
| 1442052_at | 2.22 | 0.07195 |  |  |
| 1439828_at | 2.21 | 0.07195 |  |  |
| 1442547_at | 2.21 | 0.07195 |  |  |
| 1424105_a_at | 2.21 | 0.07195 | PTTG1 | pituitary tumor-transforming 1 |
| 1442700_at | 2.21 | 0.07195 | PDE4B | phosphodiesterase 4B, cAMP-specific |
| 1442624_at | 2.21 | 0.07195 | C920008N22Rik | RIKEN cDNA C920008N22 gene |
| 1426541_a_at | 2.21 | 0.00000 | ENDOD1 | endonuclease domain containing 1 |
| 1459635_at | 2.21 | 0.07195 |  |  |
| 1453238_s_at | 2.21 | 0.07195 | A130040M12Rik | RIKEN cDNA A130040M12 gene |
| 1440013_at | 2.20 | 0.07195 |  |  |
| 1439467_at | 2.20 | 0.07195 |  |  |
| 1426415_a_at | 2.20 | 0.00000 | TRIM25 | tripartite motif containing 25 |
| 1429586_at | 2.20 | 0.07195 | 4930558N01Rik | RIKEN cDNA 4930558N01 gene |
| 1429900_at | 2.20 | 0.07195 | 5330406M23Rik | RIKEN cDNA 5330406M23 gene |
| 1460056_at | 2.19 | 0.07195 | 1700109F18Rik | RIKEN cDNA 1700109F18 gene |
| 1438104_at | 2.19 | 0.07195 | SRR | serine racemase |
| 1416355_at | 2.19 | 0.07195 | RBMX | RNA binding motif protein, X-linked |
| 1417856_at | 2.19 | 0.07195 | RELB | v-rel reticuloendotheliosis viral oncogene homolog B |
| 1430491_at | 2.19 | 0.07195 | BOP1/LOC727967 | block of proliferation 1 |
| 1426133_a_at | 2.19 | 0.07195 | MITD1 | MIT, microtubule interacting and transport, domain containing 1 |
| 1441956_s_at | 2.19 | 0.07195 | CUX1 | cut-like homeobox 1 |
| 1424906_at | 2.18 | 0.07195 | PQLC3 | PQ loop repeat containing 3 |
| 1457007_at | 2.18 | 0.07195 |  |  |
| 1441992_at | 2.18 | 0.07195 | RAB14 | RAB14, member RAS oncogene family |
| 1438508_at | 2.18 | 0.07195 |  |  |
| 1446365_at | 2.17 | 0.07195 | VTI1A | vesicle transport through interaction with t-SNAREs homolog 1A (yeast) |
| 1453475_at | 2.17 | 0.07195 |  |  |
| 1437638_at | 2.17 | 0.07195 | SRRM2 | serine/arginine repetitive matrix 2 |
| 1431432_at | 2.17 | 0.07195 | CFL2 | cofilin 2 (muscle) |
| 1444552_at | 2.17 | 0.07195 |  |  |
| 1433293_at | 2.17 | 0.07195 | SDHA | succinate dehydrogenase complex, subunit A, flavoprotein (Fp) |
| 1449632_s_at | 2.17 | 0.07195 | FKBP10 | FK506 binding protein 10, 65 kDa |
| 1440789_at | 2.17 | 0.07195 | NEO1 | neogenin 1 |
| 1424159_at | 2.17 | 0.07195 | FAM134C | family with sequence similarity 134, member C |
| 1458833_at | 2.16 | 0.07195 | NRCAM | neuronal cell adhesion molecule |
| 1436867_at | 2.16 | 0.07195 | SRL | sarcalumenin |
| 1456216_at | 2.16 | 0.07195 |  |  |
| 1448812_at | 2.16 | 0.07195 | HPCAL1 | hippocalcin-like 1 |
| 1457788_at | 2.15 | 0.07195 | Rassf1 | Ras association (RalGDS/AF-6) domain family member 1 |
| 1443533_at | 2.15 | 0.07195 |  |  |
| 1457839_at | 2.15 | 0.07195 |  |  |
| 1444570_at | 2.15 | 0.07195 |  |  |
| 1436324_at | 2.14 | 0.07195 | STARD9 | StAR-related lipid transfer (START) domain containing 9 |
| 1457900_at | 2.14 | 0.07195 | ASCC3 | activating signal cointegrator 1 complex subunit 3 |
| 1417645_at | 2.14 | 0.07195 | SSPN | sarcospan (Kras oncogene-associated gene) |
| 1416564_at | 2.14 | 0.07195 | SOX7 | SRY (sex determining region Y)-box 7 |
| 1441556_at | 2.13 | 0.07195 |  |  |
| 1423103_at | 2.13 | 0.07195 | RFX5 | regulatory factor X, 5 (influences HLA class II expression) |
| 1438336_at | 2.13 | 0.07195 | FBXW11 | F-box and WD repeat domain containing 11 |
| 1456377_x_at | 2.13 | 0.07195 | LIMD2 | LIM domain containing 2 |
| 1456282_at | 2.13 | 0.07195 | ZNF440/ZNF808 | zinc finger protein 440 |
| 1439376_x_at | 2.13 | 0.07195 | DMTF1 | cyclin D binding myb-like transcription factor 1 |
| 1444411_at | 2.13 | 0.07195 |  |  |
| AFFX-18SRNAMur/X00686_M_at | 2.13 | 0.07195 | Rn18s | 18S ribosomal RNA |
| 1439915_at | 2.13 | 0.07195 |  |  |
| 1441677_at | 2.13 | 0.07195 |  |  |
| 1435964_a_at | 2.13 | 0.07195 | TAOK3 | TAO kinase 3 |
| 1430075_at | 2.12 | 0.07195 | SF3B3 | splicing factor 3b, subunit 3, 130kDa |
| 1442679_at | 2.12 | 0.07195 |  |  |
| 1456303_at | 2.12 | 0.07195 |  |  |
| 1456655_at | 2.12 | 0.07195 |  |  |
| 1429898_at | 2.12 | 0.07195 | OSTC | oligosaccharyltransferase complex subunit |
| 1458942_at | 2.12 | 0.07195 | C230037E05Rik | RIKEN cDNA C230037E05 gene |
| 1426539_at | 2.12 | 0.07195 | USP11 | ubiquitin specific peptidase 11 |
| 1438157_s_at | 2.12 | 0.00000 | NFKBIA | nuclear factor of kappa light polypeptide gene enhancer in B-cells inhibitor, alpha |
| 1440771_at | 2.12 | 0.07195 | ZKSCAN1 | zinc finger with KRAB and SCAN domains 1 |
| 1424607_a_at | 2.12 | 0.07195 | CWC22 | CWC22 spliceosome-associated protein homolog (S. cerevisiae) |
| 1441799_at | 2.12 | 0.07195 | 6030422H21Rik | RIKEN cDNA 6030422H21 gene |
| 1440317_at | 2.11 | 0.07195 | C130068B02Rik | RIKEN cDNA C130068B02 gene |
| 1456822_at | 2.11 | 0.07195 | RAD23B | RAD23 homolog B (S. cerevisiae) |
| 1424609_a_at | 2.11 | 0.07195 | CWC22 | CWC22 spliceosome-associated protein homolog (S. cerevisiae) |
| 1445260_at | 2.11 | 0.07195 | DCUN1D1 | DCN1, defective in cullin neddylation 1, domain containing 1 (S. cerevisiae) |
| 1422512_a_at | 2.10 | 0.07195 | OGFR | opioid growth factor receptor |
| 1423747_a_at | 2.10 | 0.07195 | PDK1 | pyruvate dehydrogenase kinase, isozyme 1 |
| 1446670_at | 2.10 | 0.07195 |  |  |
| 1434277_a_at | 2.10 | 0.07195 | YPEL2 | yippee-like 2 (Drosophila) |
| 1457552_at | 2.10 | 0.07195 | ZNF295 | zinc finger protein 295 |
| 1441030_at | 2.10 | 0.07195 | RAI14 | retinoic acid induced 14 |
| 1435640_x_at | 2.10 | 0.07195 | A130040M12Rik | RIKEN cDNA A130040M12 gene |
| 1442330_at | 2.10 | 0.07195 | SRRM1 | serine/arginine repetitive matrix 1 |
| 1429861_at | 2.09 | 0.07195 | PCDH9 | protocadherin 9 |
| 1459734_at | 2.09 | 0.07195 |  |  |
| 1431300_at | 2.09 | 0.07195 | SGIP1 | SH3-domain GRB2-like (endophilin) interacting protein 1 |
| 1445626_at | 2.09 | 0.07195 |  |  |
| 1440365_at | 2.09 | 0.07195 |  |  |
| 1458292_at | 2.09 | 0.07195 | PSMA1 | proteasome (prosome, macropain) subunit, alpha type, 1 |
| 1429222_at | 2.09 | 0.07195 | PUS3 | pseudouridylate synthase 3 |
| 1443742_x_at | 2.09 | 0.07195 |  |  |
| 1429978_at | 2.08 | 0.07195 | AMN1 | antagonist of mitotic exit network 1 homolog (S. cerevisiae) |
| 1449578_at | 2.08 | 0.07195 | SUPT16H | suppressor of Ty 16 homolog (S. cerevisiae) |
| 1442811_at | 2.08 | 0.07195 | RGMB | RGM domain family, member B |
| 1426543_x_at | 2.08 | 0.07195 | ENDOD1 | endonuclease domain containing 1 |
| 1458407_s_at | 2.08 | 0.07195 |  |  |
| 1449099_at | 2.07 | 0.07195 | LRBA | LPS-responsive vesicle trafficking, beach and anchor containing |
| 1445559_at | 2.07 | 0.07195 |  |  |
| 1420136_a_at | 2.07 | 0.07195 |  |  |
| 1458406_at | 2.07 | 0.07195 |  |  |
| 1442285_at | 2.07 | 0.07195 | SYNE2 | spectrin repeat containing, nuclear envelope 2 |
| 1430216_at | 2.07 | 0.07195 | ZNF292 | zinc finger protein 292 |
| 1441912_x_at | 2.07 | 0.00000 | C2 | complement component 2 |
| 1418288_at | 2.06 | 0.07195 | LPIN1 | lipin 1 |
| 1443621_at | 2.06 | 0.07195 | XAF1 | XIAP associated factor 1 |
| 1444500_at | 2.06 | 0.07195 | AHSA1 | AHA1, activator of heat shock 90kDa protein ATPase homolog 1 (yeast) |
| 1437917_at | 2.06 | 0.07195 | D530037H12Rik | RIKEN cDNA D530037H12 gene |
| 1450857_a_at | 2.06 | 0.07195 | COL1A2 | collagen, type I, alpha 2 |
| 1441967_at | 2.06 | 0.00000 | PDDC1 | Parkinson disease 7 domain containing 1 |
| 1453513_at | 2.06 | 0.07195 |  |  |
| 1422601_at | 2.06 | 0.07195 | SERPINB9 | serpin peptidase inhibitor, clade B (ovalbumin), member 9 |
| 1445178_at | 2.06 | 0.07195 | SH3RF1 | SH3 domain containing ring finger 1 |
| 1440690_at | 2.05 | 0.07195 |  |  |
| 1439871_at | 2.05 | 0.07195 |  |  |
| 1456251_x_at | 2.05 | 0.07195 | TSPO | translocator protein (18kDa) |
| 1429968_at | 2.05 | 0.07195 | JKAMP | JNK1/MAPK8-associated membrane protein |
| 1456843_at | 2.05 | 0.07195 | YES1 | v-yes-1 Yamaguchi sarcoma viral oncogene homolog 1 |
| 1425362_at | 2.05 | 0.07195 | AGFG2 | ArfGAP with FG repeats 2 |
| 1445512_at | 2.05 | 0.07195 |  |  |
| 1458436_at | 2.05 | 0.07195 | AUH | AU RNA binding protein/enoyl-CoA hydratase |
| 1458077_at | 2.05 | 0.07195 |  |  |
| 1417821_at | 2.04 | 0.07195 | D17H6S56E-5 | DNA segment, Chr 17, human D6S56E 5 |
| 1437798_at | 2.04 | 0.07195 | 6720422M22Rik | RIKEN cDNA 6720422M22 gene |
| 1420088_at | 2.04 | 0.07195 | NFKBIA | nuclear factor of kappa light polypeptide gene enhancer in B-cells inhibitor, alpha |
| 1447873_x_at | 2.04 | 0.07195 | BID | BH3 interacting domain death agonist |
| 1444352_at | 2.04 | 0.07195 | ZNF287 | zinc finger protein 287 |
| 1440091_at | 2.04 | 0.07195 | MEIS2 | Meis homeobox 2 |
| 1439127_at | 2.04 | 0.07195 | KIAA0368 | KIAA0368 |
| 1442568_at | 2.03 | 0.07195 |  |  |
| 1442590_at | 2.03 | 0.07195 | Tnfrsf22/Tnfrsf23 | tumor necrosis factor receptor superfamily, member 22 |
| 1455425_at | 2.03 | 0.07195 | TET1 | tet oncogene 1 |
| 1415951_at | 2.03 | 0.07195 | FKBP10 | FK506 binding protein 10, 65 kDa |
| 1421005_at | 2.03 | 0.00000 | CEP110 | centrosomal protein 110kDa |
| 1436520_at | 2.03 | 0.07195 | AHNAK2 | AHNAK nucleoprotein 2 |
| 1433668_at | 2.03 | 0.07195 | PNRC1 | proline-rich nuclear receptor coactivator 1 |
| 1436631_at | 2.03 | 0.07195 | CTNNA1 | catenin (cadherin-associated protein), alpha 1, 102kDa |
| 1437554_at | 2.03 | 0.07195 | PLEC | plectin |
| 1458562_at | 2.03 | 0.07195 |  |  |
| 1451362_at | 2.02 | 0.07195 | RAB7L1 | RAB7, member RAS oncogene family-like 1 |
| 1442944_at | 2.02 | 0.07195 | C76555 | expressed sequence C76555 |
| 1433338_at | 2.02 | 0.07195 | 6720460K10Rik | RIKEN cDNA 6720460K10 gene |
| 1420973_at | 2.02 | 0.07195 | ARID5B | AT rich interactive domain 5B (MRF1-like) |
| 1438301_at | 2.02 | 0.07195 |  |  |
| 1457214_at | 2.02 | 0.07195 |  |  |
| 1421679_a_at | 2.02 | 0.07195 | CDKN1A | cyclin-dependent kinase inhibitor 1A (p21, Cip1) |
| 1417581_at | 2.02 | 0.07195 | DHODH | dihydroorotate dehydrogenase |
| 1440573_at | 2.01 | 0.07195 |  |  |
| 1415713_a_at | 2.01 | 0.07195 | DDX24 | DEAD (Asp-Glu-Ala-Asp) box polypeptide 24 |
| 1441592_at | 2.01 | 0.07195 |  |  |
| 1440841_at | 2.01 | 0.07195 | BB217526 | expressed sequence BB217526 |
| 1456955_at | 2.01 | 0.07195 |  |  |
| 1421812_at | 2.00 | 0.07195 | TAPBP | TAP binding protein (tapasin) |
| 1420161_at | 2.00 | 0.07195 | AA409749 | expressed sequence AA409749 |
| 1457817_at | 2.00 | 0.07195 |  |  |
| 1436357_at | 2.00 | 0.07195 | Gm10374 | predicted gene 10374 |
| 1425538_x_at | 2.00 | 0.07195 | Ceacam1 | carcinoembryonic antigen-related cell adhesion molecule 1 |
| 1460555_at | 2.00 | 0.07195 | FAM65B | family with sequence similarity 65, member B |
| 1436279_at | -2.00 | 0.01872 | SLC26A7 | solute carrier family 26, member 7 |
| 1435644_at | -2.00 | 0.01872 | SH3PXD2B | SH3 and PX domains 2B |
| 1430147_a_at | -2.00 | 0.01872 | TAF1D | TATA box binding protein (TBP)-associated factor, RNA polymerase I, D, 41kDa |
| 1436719_at | -2.01 | 0.01872 | SLC35F1 | solute carrier family 35, member F1 |
| 1428383_a_at | -2.01 | 0.01872 | KIAA0913 | KIAA0913 |
| 1440859_at | -2.01 | 0.01872 | AKAP6 | A kinase (PRKA) anchor protein 6 |
| 1430031_at | -2.01 | 0.05544 | IPMK | inositol polyphosphate multikinase |
| 1439942_at | -2.01 | 0.01872 | PREP | prolyl endopeptidase |
| 1436706_at | -2.01 | 0.01872 | MMGT1 | membrane magnesium transporter 1 |
| 1430271_x_at | -2.01 | 0.01872 | TAF1D | TATA box binding protein (TBP)-associated factor, RNA polymerase I, D, 41kDa |
| 1428467_at | -2.02 | 0.01872 | TARDBP | TAR DNA binding protein |
| 1420834_at | -2.02 | 0.01872 | VAMP2 | vesicle-associated membrane protein 2 (synaptobrevin 2) |
| 1460228_at | -2.02 | 0.01872 | USF2 | upstream transcription factor 2, c-fos interacting |
| 1451847_s_at | -2.02 | 0.01872 | ARID4B | AT rich interactive domain 4B (RBP1-like) |
| 1426301_at | -2.02 | 0.01872 | ALCAM | activated leukocyte cell adhesion molecule |
| 1450533_a_at | -2.02 | 0.01872 | PLAGL1 | pleiomorphic adenoma gene-like 1 |
| 1459900_at | -2.02 | 0.02566 | C79468 | expressed sequence C79468 |
| 1421846_at | -2.02 | 0.01872 | WSB2 | WD repeat and SOCS box containing 2 |
| 1419061_at | -2.03 | 0.01872 | RHOD | ras homolog gene family, member D |
| 1437329_at | -2.03 | 0.01872 | PTPLB | protein tyrosine phosphatase-like (proline instead of catalytic arginine), member b |
| 1421340_at | -2.03 | 0.02566 | MAP3K5 | mitogen-activated protein kinase kinase kinase 5 |
| 1421847_at | -2.03 | 0.01872 | WSB2 | WD repeat and SOCS box containing 2 |
| 1430516_at | -2.03 | 0.02566 | 4930428B01Rik | RIKEN cDNA 4930428B01 gene |
| 1418251_at | -2.03 | 0.01872 | TULP3 | tubby like protein 3 |
| 1424380_at | -2.04 | 0.01872 | VPS37B | vacuolar protein sorting 37 homolog B (S. cerevisiae) |
| 1424542_at | -2.04 | 0.01872 | S100A4 | S100 calcium binding protein A4 |
| 1425986_a_at | -2.04 | 0.01872 | DCUN1D1 | DCN1, defective in cullin neddylation 1, domain containing 1 (S. cerevisiae) |
| 1455983_at | -2.04 | 0.02566 | CDCA2 | cell division cycle associated 2 |
| 1428940_at | -2.05 | 0.01872 | GNAQ | guanine nucleotide binding protein (G protein), q polypeptide |
| 1416778_at | -2.05 | 0.01872 | SDPR | serum deprivation response |
| 1417848_at | -2.06 | 0.01872 | ZNF704 | zinc finger protein 704 |
| 1435696_s_at | -2.06 | 0.01872 | SREK1IP1 | SREK1-interacting protein 1 |
| 1430214_a_at | -2.06 | 0.01872 | LOC81691 | exonuclease NEF-sp |
| 1417662_at | -2.06 | 0.03566 | ELK3 | ELK3, ETS-domain protein (SRF accessory protein 2) |
| 1435437_at | -2.07 | 0.01872 | SETD7 | SET domain containing (lysine methyltransferase) 7 |
| 1428649_at | -2.07 | 0.01872 | CAND1 | cullin-associated and neddylation-dissociated 1 |
| 1416701_at | -2.07 | 0.01872 | RND3 | Rho family GTPase 3 |
| 1425666_at | -2.07 | 0.01872 | ZIC5 | Zic family member 5 (odd-paired homolog, Drosophila) |
| 1428914_at | -2.07 | 0.01872 | SH3PXD2A | SH3 and PX domains 2A |
| 1452387_a_at | -2.07 | 0.01872 | AMOTL2 | angiomotin like 2 |
| 1452666_a_at | -2.08 | 0.01872 | TMCC2 | transmembrane and coiled-coil domain family 2 |
| 1435938_at | -2.08 | 0.01872 | CKAP2L | cytoskeleton associated protein 2-like |
| 1452863_at | -2.08 | 0.01872 | C20orf144 | chromosome 20 open reading frame 144 |
| 1435384_at | -2.09 | 0.01872 | UBE2N | ubiquitin-conjugating enzyme E2N (UBC13 homolog, yeast) |
| 1442046_at | -2.09 | 0.01872 | SHROOM2 | shroom family member 2 |
| 1419678_at | -2.09 | 0.01872 | LATS2 | LATS, large tumor suppressor, homolog 2 (Drosophila) |
| 1440478_at | -2.09 | 0.02566 | NUS1 | nuclear undecaprenyl pyrophosphate synthase 1 homolog (S. cerevisiae) |
| 1431200_a_at | -2.10 | 0.03566 | DPH3 | DPH3, KTI11 homolog (S. cerevisiae) |
| 1429404_at | -2.10 | 0.01872 | C9orf140 | chromosome 9 open reading frame 140 |
| 1457716_at | -2.10 | 0.03566 | OTUD7B | OTU domain containing 7B |
| 1417196_s_at | -2.10 | 0.01872 | WWC2 | WW and C2 domain containing 2 |
| 1454997_at | -2.10 | 0.02566 | MSRB3 | methionine sulfoxide reductase B3 |
| 1428710_at | -2.10 | 0.02566 | RIT1 | Ras-like without CAAX 1 |
| 1428925_at | -2.11 | 0.01872 | SENP1 | SUMO1/sentrin specific peptidase 1 |
| 1436300_at | -2.11 | 0.01872 | DSTYK | dual serine/threonine and tyrosine protein kinase |
| 1436222_at | -2.11 | 0.01872 | GAS5 | growth arrest-specific 5 (non-protein coding) |
| 1428571_at | -2.11 | 0.05544 | COL9A1 | collagen, type IX, alpha 1 |
| 1417395_at | -2.11 | 0.01872 | KLF4 | Kruppel-like factor 4 (gut) |
| 1452764_at | -2.11 | 0.02566 | SOCS6 | suppressor of cytokine signaling 6 |
| 1427414_at | -2.11 | 0.01872 | PRKAR2A | protein kinase, cAMP-dependent, regulatory, type II, alpha |
| 1437733_at | -2.11 | 0.01872 | EIF4EBP2 | eukaryotic translation initiation factor 4E binding protein 2 |
| 1421727_at | -2.11 | 0.01872 | EYA1 | eyes absent homolog 1 (Drosophila) |
| 1435946_at | -2.11 | 0.01872 | SEPSECS | Sep (O-phosphoserine) tRNA:Sec (selenocysteine) tRNA synthase |
| 1452449_at | -2.12 | 0.01872 | HMBOX1 | homeobox containing 1 |
| 1455014_at | -2.12 | 0.03566 | HINT3 | histidine triad nucleotide binding protein 3 |
| 1434830_at | -2.12 | 0.01872 | MXD1 | MAX dimerization protein 1 |
| 1422994_at | -2.12 | 0.02566 | PIKFYVE | phosphoinositide kinase, FYVE finger containing |
| 1450780_s_at | -2.13 | 0.01872 | HMGA2 | high mobility group AT-hook 2 |
| 1416515_at | -2.13 | 0.05544 | FSCN1 | fascin homolog 1, actin-bundling protein (Strongylocentrotus purpuratus) |
| 1460717_at | -2.13 | 0.01872 | TSPYL1 | TSPY-like 1 |
| 1423297_at | -2.13 | 0.01872 | ADD3 | adducin 3 (gamma) |
| 1448547_at | -2.13 | 0.02566 | RASSF3 | Ras association (RalGDS/AF-6) domain family member 3 |
| 1419365_at | -2.13 | 0.05544 | PEX11A | peroxisomal biogenesis factor 11 alpha |
| 1435768_at | -2.13 | 0.01872 | ARID4B | AT rich interactive domain 4B (RBP1-like) |
| 1417568_at | -2.14 | 0.01872 | NCALD | neurocalcin delta |
| 1426791_at | -2.14 | 0.01872 | RUSC2 | RUN and SH3 domain containing 2 |
| 1434506_at | -2.14 | 0.01872 | ARID2 | AT rich interactive domain 2 (ARID, RFX-like) |
| 1418132_a_at | -2.14 | 0.05544 | UBFD1 | ubiquitin family domain containing 1 |
| 1418294_at | -2.15 | 0.01872 | EPB41L4B | erythrocyte membrane protein band 4.1 like 4B |
| 1437885_at | -2.15 | 0.01872 | D030029J20Rik | RIKEN cDNA D030029J20 gene |
| 1423596_at | -2.15 | 0.01872 | NEK6 | NIMA (never in mitosis gene a)-related kinase 6 |
| 1423707_at | -2.15 | 0.01872 | TMEM50B | transmembrane protein 50B |
| 1442156_at | -2.15 | 0.01872 | E030030I06Rik | RIKEN cDNA E030030I06 gene |
| 1436103_at | -2.15 | 0.02566 | RAB3IP | RAB3A interacting protein (rabin3) |
| 1421935_at | -2.15 | 0.01872 | Rps20 | ribosomal protein S20 |
| 1424580_at | -2.16 | 0.01872 | SLC35A3 | solute carrier family 35 (UDP-N-acetylglucosamine (UDP-GlcNAc) transporter), member A3 |
| 1456067_at | -2.16 | 0.01872 | GLI3 | GLI family zinc finger 3 |
| 1435157_at | -2.16 | 0.01872 | HOOK3 | hook homolog 3 (Drosophila) |
| 1429096_at | -2.16 | 0.01872 | 2810455D13Rik | RIKEN cDNA 2810455D13 gene |
| 1427973_s_at | -2.17 | 0.01872 | CDC73 | cell division cycle 73, Paf1/RNA polymerase II complex component, homolog (S. cerevisiae) |
| 1426513_at | -2.17 | 0.01872 | RBM28 | RNA binding motif protein 28 |
| 1455420_at | -2.17 | 0.01872 | RAD23B | RAD23 homolog B (S. cerevisiae) |
| 1436427_at | -2.18 | 0.01872 | PRPF4B | PRP4 pre-mRNA processing factor 4 homolog B (yeast) |
| 1428573_at | -2.18 | 0.02566 | Chn2 | chimerin (chimaerin) 2 |
| 1449257_at | -2.18 | 0.01872 | FAM104A | family with sequence similarity 104, member A |
| 1425098_at | -2.18 | 0.02566 | ZFP106 | zinc finger protein 106 homolog (mouse) |
| 1423831_at | -2.18 | 0.01872 | PRKAG2 | protein kinase, AMP-activated, gamma 2 non-catalytic subunit |
| 1442870_at | -2.19 | 0.02566 |  |  |
| 1434789_at | -2.19 | 0.01872 | DEPDC1B | DEP domain containing 1B |
| 1431358_at | -2.19 | 0.01872 | C12orf48 | chromosome 12 open reading frame 48 |
| 1455625_at | -2.19 | 0.01872 | TAF10 | TAF10 RNA polymerase II, TATA box binding protein (TBP)-associated factor, 30kDa |
| 1433735_a_at | -2.19 | 0.01872 | TMEM64 | transmembrane protein 64 |
| 1419203_at | -2.19 | 0.01872 | Gm16516 | predicted gene, Gm16516 |
| 1436341_at | -2.19 | 0.01872 | HMBOX1 | homeobox containing 1 |
| 1426352_s_at | -2.19 | 0.02566 | TIAL1 | TIA1 cytotoxic granule-associated RNA binding protein-like 1 |
| 1428105_at | -2.19 | 0.01872 | TPX2 | TPX2, microtubule-associated, homolog (Xenopus laevis) |
| 1455034_at | -2.19 | 0.01872 | NR4A2 | nuclear receptor subfamily 4, group A, member 2 |
| 1437627_at | -2.20 | 0.01872 | MEX3D | mex-3 homolog D (C. elegans) |
| 1449896_at | -2.20 | 0.01872 | MLPH | melanophilin |
| 1427984_at | -2.21 | 0.01872 | SENP6 | SUMO1/sentrin specific peptidase 6 |
| 1437570_at | -2.21 | 0.03566 | AI503301 | expressed sequence AI503301 |
| 1450037_at | -2.21 | 0.01872 | USP9X | ubiquitin specific peptidase 9, X-linked |
| 1423100_at | -2.22 | 0.01872 | FOS | FBJ murine osteosarcoma viral oncogene homolog |
| 1435554_at | -2.22 | 0.01872 | TMCC3 | transmembrane and coiled-coil domain family 3 |
| 1424246_a_at | -2.22 | 0.01872 | TES | testis derived transcript (3 LIM domains) |
| 1455667_at | -2.22 | 0.05544 | PREB | prolactin regulatory element binding |
| 1425826_a_at | -2.23 | 0.01872 | SORBS1 | sorbin and SH3 domain containing 1 |
| 1453186_at | -2.23 | 0.01872 | SFRS18 | splicing factor, arginine/serine-rich 18 |
| 1421146_at | -2.23 | 0.03566 | RAPGEF1 | Rap guanine nucleotide exchange factor (GEF) 1 |
| 1416039_x_at | -2.23 | 0.01872 | CYR61 | cysteine-rich, angiogenic inducer, 61 |
| 1436424_at | -2.25 | 0.01872 | 1600020E01Rik | RIKEN cDNA 1600020E01 gene |
| 1428774_at | -2.25 | 0.01872 | GPC6 | glypican 6 |
| 1435679_at | -2.25 | 0.01872 | OPTN | optineurin |
| 1428819_at | -2.26 | 0.01872 | MAPRE1 | microtubule-associated protein, RP/EB family, member 1 |
| 1454982_at | -2.26 | 0.01872 | ARFGEF2 | ADP-ribosylation factor guanine nucleotide-exchange factor 2 (brefeldin A-inhibited) |
| 1449931_at | -2.26 | 0.01872 | CPEB4 | cytoplasmic polyadenylation element binding protein 4 |
| 1456200_at | -2.26 | 0.01872 | IPMK | inositol polyphosphate multikinase |
| 1420159_at | -2.27 | 0.05544 | MYO1E | myosin IE |
| 1431102_at | -2.27 | 0.05544 | CEP350 | centrosomal protein 350kDa |
| 1443579_s_at | -2.27 | 0.01872 | DEPTOR | DEP domain containing MTOR-interacting protein |
| 1426647_at | -2.28 | 0.05544 | C19orf42 | chromosome 19 open reading frame 42 |
| 1436108_at | -2.28 | 0.01872 | TXNDC9 | thioredoxin domain containing 9 |
| 1448611_at | -2.28 | 0.01872 | WWC2 | WW and C2 domain containing 2 |
| 1434200_at | -2.29 | 0.01872 | BC010981 | cDNA sequence BC010981 |
| 1450750_a_at | -2.29 | 0.02566 | NR4A2 | nuclear receptor subfamily 4, group A, member 2 |
| 1437658_a_at | -2.30 | 0.01872 | SNORD22 | small nucleolar RNA, C/D box 22 |
| 1444108_at | -2.30 | 0.01872 | DNAJC25 | DnaJ (Hsp40) homolog, subfamily C , member 25 |
| 1429139_at | -2.31 | 0.01872 | OTUD7B | OTU domain containing 7B |
| 1420414_at | -2.31 | 0.01872 | HOXA11 | homeobox A11 |
| 1436580_at | -2.31 | 0.01872 | HGSNAT | heparan-alpha-glucosaminide N-acetyltransferase |
| 1454646_at | -2.31 | 0.01872 | TCP11L2 | t-complex 11 (mouse)-like 2 |
| 1452514_a_at | -2.31 | 0.02566 | KIT | v-kit Hardy-Zuckerman 4 feline sarcoma viral oncogene homolog |
| 1424481_s_at | -2.31 | 0.01872 | ARMCX5 | armadillo repeat containing, X-linked 5 |
| 1432430_a_at | -2.32 | 0.01872 | KIAA1267 | KIAA1267 |
| 1435186_s_at | -2.32 | 0.03566 | LRRCC1 | leucine rich repeat and coiled-coil domain containing 1 |
| 1439129_at | -2.35 | 0.01872 | DOCK5 | dedicator of cytokinesis 5 |
| 1421529_a_at | -2.36 | 0.01872 | TXNRD1 | thioredoxin reductase 1 |
| 1453760_at | -2.36 | 0.01872 | MIER1 | mesoderm induction early response 1 homolog (Xenopus laevis) |
| 1416514_a_at | -2.37 | 0.01872 | FSCN1 | fascin homolog 1, actin-bundling protein (Strongylocentrotus purpuratus) |
| 1429591_at | -2.37 | 0.03566 | TACC1 | transforming, acidic coiled-coil containing protein 1 |
| 1450156_a_at | -2.37 | 0.01872 | HMMR | hyaluronan-mediated motility receptor (RHAMM) |
| 1419621_at | -2.37 | 0.01872 | ANKRD2 | ankyrin repeat domain 2 (stretch responsive muscle) |
| 1418726_a_at | -2.37 | 0.01872 | TNNT2 | troponin T type 2 (cardiac) |
| 1416521_at | -2.38 | 0.01872 | SEPW1 | selenoprotein W, 1 |
| 1424486_a_at | -2.39 | 0.01872 | TXNRD1 | thioredoxin reductase 1 |
| 1425285_a_at | -2.39 | 0.01872 | RAB27A | RAB27A, member RAS oncogene family |
| 1439024_at | -2.39 | 0.01872 | BAG4 | BCL2-associated athanogene 4 |
| 1425284_a_at | -2.40 | 0.01872 | RAB27A | RAB27A, member RAS oncogene family |
| 1426767_at | -2.40 | 0.01872 | WDR90 | WD repeat domain 90 |
| 1421339_at | -2.40 | 0.01872 | EXTL3 | exostoses (multiple)-like 3 |
| 1450976_at | -2.42 | 0.01872 | NDRG1 | N-myc downstream regulated 1 |
| 1450157_a_at | -2.43 | 0.01872 | HMMR | hyaluronan-mediated motility receptor (RHAMM) |
| 1417195_at | -2.43 | 0.01872 | WWC2 | WW and C2 domain containing 2 |
| 1435981_at | -2.43 | 0.01872 | Nav2 | neuron navigator 2 |
| 1448662_at | -2.43 | 0.01872 | FZD6 | frizzled homolog 6 (Drosophila) |
| 1428834_at | -2.43 | 0.01872 | DUSP4 | dual specificity phosphatase 4 |
| 1424005_at | -2.44 | 0.01872 | C5orf24 | chromosome 5 open reading frame 24 |
| 1426226_at | -2.44 | 0.01872 | DYRK1A | dual-specificity tyrosine-(Y)-phosphorylation regulated kinase 1A |
| 1421237_at | -2.44 | 0.01872 | TMPO | thymopoietin |
| 1448069_at | -2.44 | 0.02566 | TM4SF1 | transmembrane 4 L six family member 1 |
| 1423298_at | -2.46 | 0.01872 | ADD3 | adducin 3 (gamma) |
| 1437002_at | -2.47 | 0.01872 | FAM73A | family with sequence similarity 73, member A |
| 1427541_x_at | -2.47 | 0.01872 | HMMR | hyaluronan-mediated motility receptor (RHAMM) |
| 1428990_at | -2.48 | 0.02566 | 2310047K21Rik | RIKEN cDNA 2310047K21 gene |
| 1456563_at | -2.48 | 0.03566 | ARHGEF37 | Rho guanine nucleotide exchange factor (GEF) 37 |
| 1437290_at | -2.48 | 0.01872 | IMPAD1 | inositol monophosphatase domain containing 1 |
| 1422705_at | -2.49 | 0.02566 | PMEPA1 | prostate transmembrane protein, androgen induced 1 |
| 1437024_at | -2.49 | 0.01872 | SMPD4 | sphingomyelin phosphodiesterase 4, neutral membrane (neutral sphingomyelinase-3) |
| 1428888_at | -2.50 | 0.01872 | TMEM33 | transmembrane protein 33 |
| 1421171_at | -2.50 | 0.01872 | ADAM12 | ADAM metallopeptidase domain 12 |
| 1456830_at | -2.51 | 0.01872 | Ppp1r2 | protein phosphatase 1, regulatory (inhibitor) subunit 2 |
| 1434559_at | -2.51 | 0.01872 | STX3 | syntaxin 3 |
| 1439925_at | -2.52 | 0.01872 | TM4SF1 | transmembrane 4 L six family member 1 |
| 1425668_a_at | -2.52 | 0.01872 | ST3GAL4 | ST3 beta-galactoside alpha-2,3-sialyltransferase 4 |
| 1420477_at | -2.53 | 0.01872 | NAP1L1 | nucleosome assembly protein 1-like 1 |
| 1451596_a_at | -2.53 | 0.01872 | SPHK1 | sphingosine kinase 1 |
| 1417943_at | -2.53 | 0.02566 | GNG4 | guanine nucleotide binding protein (G protein), gamma 4 |
| 1425369_a_at | -2.54 | 0.01872 | SOX10 | SRY (sex determining region Y)-box 10 |
| 1420998_at | -2.55 | 0.03566 | ETV5 | ets variant 5 |
| 1448546_at | -2.55 | 0.01872 | RASSF3 | Ras association (RalGDS/AF-6) domain family member 3 |
| 1454628_at | -2.56 | 0.02566 | IFFO1 | intermediate filament family orphan 1 |
| 1420760_s_at | -2.56 | 0.01872 | NDRG1 | N-myc downstream regulated 1 |
| 1449576_at | -2.57 | 0.01872 | EIF1AX | eukaryotic translation initiation factor 1A, X-linked |
| 1454709_at | -2.57 | 0.01872 | TMEM64 | transmembrane protein 64 |
| 1458599_at | -2.58 | 0.02566 |  |  |
| 1435398_at | -2.59 | 0.01872 | STXBP5 | syntaxin binding protein 5 (tomosyn) |
| 1434307_at | -2.60 | 0.01872 | TMEM64 | transmembrane protein 64 |
| 1457275_at | -2.61 | 0.01872 | SYNM | synemin, intermediate filament protein |
| 1441275_at | -2.62 | 0.02566 | KBTBD8 | kelch repeat and BTB (POZ) domain containing 8 |
| 1426965_at | -2.63 | 0.01872 | RAP2A | RAP2A, member of RAS oncogene family |
| 1422739_at | -2.64 | 0.01872 | HS2ST1 | heparan sulfate 2-O-sulfotransferase 1 |
| 1437227_at | -2.64 | 0.05544 | URM1 | ubiquitin related modifier 1 |
| 1424263_at | -2.64 | 0.01872 | AIF1L | allograft inflammatory factor 1-like |
| 1458882_at | -2.64 | 0.01872 | SERPINB8 | serpin peptidase inhibitor, clade B (ovalbumin), member 8 |
| 1431022_at | -2.66 | 0.01872 | SNCA | synuclein, alpha (non A4 component of amyloid precursor) |
| 1434253_s_at | -2.67 | 0.01872 | TMCC3 | transmembrane and coiled-coil domain family 3 |
| 1445328_at | -2.67 | 0.03566 | COL4A4 | collagen, type IV, alpha 4 |
| 1429443_at | -2.68 | 0.03566 | CPNE4 | copine IV |
| 1438132_at | -2.69 | 0.01872 | Gm5089 | predicted gene 5089 |
| 1424967_x_at | -2.69 | 0.02566 | TNNT2 | troponin T type 2 (cardiac) |
| 1419228_at | -2.71 | 0.05544 | ELAC1 | elaC homolog 1 (E. coli) |
| 1438796_at | -2.72 | 0.01872 | NR4A3 | nuclear receptor subfamily 4, group A, member 3 |
| 1421841_at | -2.73 | 0.02566 | FGFR3 | fibroblast growth factor receptor 3 |
| 1420579_s_at | -2.74 | 0.01872 | CFTR | cystic fibrosis transmembrane conductance regulator (ATP-binding cassette sub-family C, member 7) |
| 1438133_a_at | -2.76 | 0.01872 | CYR61 | cysteine-rich, angiogenic inducer, 61 |
| 1454917_at | -2.78 | 0.01872 | ALG10 | asparagine-linked glycosylation 10, alpha-1,2-glucosyltransferase homolog (S. pombe) |
| 1435321_at | -2.78 | 0.01872 | LIMCH1 | LIM and calponin homology domains 1 |
| 1434294_at | -2.79 | 0.02566 | FAM199X | family with sequence similarity 199, X-linked |
| 1423389_at | -2.80 | 0.01872 | SMAD7 | SMAD family member 7 |
| 1452015_at | -2.81 | 0.01872 | C9orf91 | chromosome 9 open reading frame 91 |
| 1422249_s_at | -2.83 | 0.01872 | ZFX | zinc finger protein, X-linked |
| 1443915_at | -2.83 | 0.02566 | MRPL47 | mitochondrial ribosomal protein L47 |
| 1417197_at | -2.83 | 0.01872 | WWC2 | WW and C2 domain containing 2 |
| 1457124_at | -2.84 | 0.01872 |  |  |
| 1450155_at | -2.85 | 0.05544 | ITGA4 | integrin, alpha 4 (antigen CD49D, alpha 4 subunit of VLA-4 receptor) |
| 1435641_at | -2.87 | 0.01872 | MGAT4A | mannosyl (alpha-1,3-)-glycoprotein beta-1,4-N-acetylglucosaminyltransferase, isozyme A |
| 1435781_at | -2.88 | 0.01872 | CAND1 | cullin-associated and neddylation-dissociated 1 |
| 1417394_at | -2.88 | 0.01872 | KLF4 | Kruppel-like factor 4 (gut) |
| 1428081_at | -2.88 | 0.01872 | KLHL21 | kelch-like 21 (Drosophila) |
| 1450730_at | -2.88 | 0.01872 | HS2ST1 | heparan sulfate 2-O-sulfotransferase 1 |
| 1417801_a_at | -2.90 | 0.01872 | PPFIBP2 | PTPRF interacting protein, binding protein 2 (liprin beta 2) |
| 1434252_at | -2.91 | 0.01872 | TMCC3 | transmembrane and coiled-coil domain family 3 |
| 1442030_at | -2.91 | 0.01872 |  |  |
| 1418731_at | -2.91 | 0.01872 | RLIM | ring finger protein, LIM domain interacting |
| 1422537_a_at | -2.93 | 0.01872 | ID2 | inhibitor of DNA binding 2, dominant negative helix-loop-helix protein |
| 1427679_at | -2.93 | 0.01872 | LATS1 | LATS, large tumor suppressor, homolog 1 (Drosophila) |
| 1451794_at | -2.93 | 0.01872 | TMCC3 | transmembrane and coiled-coil domain family 3 |
| 1434657_at | -2.93 | 0.01872 | GLS | glutaminase |
| 1460365_a_at | -2.94 | 0.01872 | DNM1 | dynamin 1 |
| 1431068_at | -2.95 | 0.01872 | RMND5A | required for meiotic nuclear division 5 homolog A (S. cerevisiae) |
| 1453736_s_at | -2.97 | 0.01872 | C5orf24 | chromosome 5 open reading frame 24 |
| 1432249_a_at | -2.98 | 0.01872 | ERCC8 | excision repair cross-complementing rodent repair deficiency, complementation group 8 |
| 1429530_a_at | -2.99 | 0.01872 | SMPD4 | sphingomyelin phosphodiesterase 4, neutral membrane (neutral sphingomyelinase-3) |
| 1452858_at | -3.00 | 0.01872 | ELAVL1 | ELAV (embryonic lethal, abnormal vision, Drosophila)-like 1 (Hu antigen R) |
| 1428149_at | -3.01 | 0.01872 | CORO7 | coronin 7 |
| 1437466_at | -3.01 | 0.01872 | ALCAM | activated leukocyte cell adhesion molecule |
| 1448375_at | -3.01 | 0.01872 | TM9SF3 | transmembrane 9 superfamily member 3 |
| 1418252_at | -3.04 | 0.01872 | PADI2 | peptidyl arginine deiminase, type II |
| 1422054_a_at | -3.05 | 0.01872 | SKIL | SKI-like oncogene |
| 1419163_s_at | -3.10 | 0.01872 | DNAJC3 | DnaJ (Hsp40) homolog, subfamily C, member 3 |
| 1441327_a_at | -3.11 | 0.01872 | SSR1 | signal sequence receptor, alpha |
| 1449373_at | -3.11 | 0.01872 | DNAJC3 | DnaJ (Hsp40) homolog, subfamily C, member 3 |
| 1450229_at | -3.13 | 0.01872 | MED14 | mediator complex subunit 14 |
| 1429021_at | -3.16 | 0.02566 | EPHA4 | EPH receptor A4 |
| 1429468_at | -3.16 | 0.01872 | DNAJB14 | DnaJ (Hsp40) homolog, subfamily B, member 14 |
| 1421995_at | -3.17 | 0.01872 | TFAP2A | transcription factor AP-2 alpha (activating enhancer binding protein 2 alpha) |
| 1441751_at | -3.18 | 0.05544 | AW493563 | expressed sequence AW493563 |
| 1440195_at | -3.18 | 0.01872 | SERBP1 | SERPINE1 mRNA binding protein 1 |
| 1419191_at | -3.20 | 0.01872 | HIPK3 | homeodomain interacting protein kinase 3 |
| 1424704_at | -3.20 | 0.02566 | RUNX2 | runt-related transcription factor 2 |
| 1425895_a_at | -3.22 | 0.01872 | ID1 | inhibitor of DNA binding 1, dominant negative helix-loop-helix protein |
| 1441899_x_at | -3.22 | 0.01872 | BCAN | brevican |
| 1420961_a_at | -3.22 | 0.01872 | IVNS1ABP | influenza virus NS1A binding protein |
| 1434404_at | -3.26 | 0.01872 | FAM73A | family with sequence similarity 73, member A |
| 1436871_at | -3.28 | 0.01872 | SRSF7 | serine/arginine-rich splicing factor 7 |
| 1435590_at | -3.28 | 0.01872 | DCUN1D5 | DCN1, defective in cullin neddylation 1, domain containing 5 (S. cerevisiae) |
| 1423322_at | -3.29 | 0.01872 | LIN7C | lin-7 homolog C (C. elegans) |
| 1450937_at | -3.37 | 0.01872 | LIN7C | lin-7 homolog C (C. elegans) |
| 1428574_a_at | -3.42 | 0.01872 | Chn2 | chimerin (chimaerin) 2 |
| 1421492_at | -3.46 | 0.01872 | HPGDS | hematopoietic prostaglandin D synthase |
| 1438502_x_at | -3.47 | 0.01872 | Rps17 | ribosomal protein S17 |
| 1417198_at | -3.63 | 0.01872 | WWC2 | WW and C2 domain containing 2 |
| 1417358_s_at | -3.67 | 0.01872 | SORBS1 | sorbin and SH3 domain containing 1 |
| 1416846_a_at | -3.73 | 0.00000 | PDZRN3 | PDZ domain containing ring finger 3 |
| 1449262_s_at | -3.75 | 0.01872 | LIN7C | lin-7 homolog C (C. elegans) |
| 1435763_at | -3.88 | 0.01872 | TBC1D16 | TBC1 domain family, member 16 |
| 1452042_a_at | -3.91 | 0.01872 | TMEM144 | transmembrane protein 144 |
| 1450781_at | -3.91 | 0.01872 | HMGA2 | high mobility group AT-hook 2 |
| 1422706_at | -3.93 | 0.01872 | PMEPA1 | prostate transmembrane protein, androgen induced 1 |
| 1431031_at | -3.94 | 0.01872 | ARID4B | AT rich interactive domain 4B (RBP1-like) |
| 1455059_at | -3.97 | 0.01872 | HELZ | helicase with zinc finger |
| 1434553_at | -4.09 | 0.01872 | TMEM56 | transmembrane protein 56 |
| 1426362_at | -4.20 | 0.01872 | TMEM144 | transmembrane protein 144 |
| 1441501_at | -4.20 | 0.01872 | SERBP1 | SERPINE1 mRNA binding protein 1 |
| 1437318_at | -4.43 | 0.01872 | PAK3 | p21 protein (Cdc42/Rac)-activated kinase 3 |
| 1440626_at | -4.48 | 0.01872 | HOXD13 | homeobox D13 |
| 1435162_at | -4.65 | 0.01872 | PRKG2 | protein kinase, cGMP-dependent, type II |
| 1438501_at | -4.71 | 0.01872 | Rps17 | ribosomal protein S17 |
| 1451287_s_at | -4.76 | 0.01872 | AIF1L | allograft inflammatory factor 1-like |
| 1421943_at | -4.98 | 0.02566 | TGFA | transforming growth factor, alpha |
| 1430357_at | -5.04 | 0.01872 | H3F3C | H3 histone, family 3C |
| 1416630_at | -5.07 | 0.01872 | ID3 | inhibitor of DNA binding 3, dominant negative helix-loop-helix protein |
| 1439793_at | -5.62 | 0.01872 | GJA3 | gap junction protein, alpha 3, 46kDa |
| 1441971_at | -5.62 | 0.01872 |  |  |
| 1435511_at | -5.90 | 0.01872 | SYN2 | synapsin II |
| 1458518_at | -6.05 | 0.01872 | CPEB2 | cytoplasmic polyadenylation element binding protein 2 |
| 1437071_at | -6.71 | 0.01872 | EIF1AX | eukaryotic translation initiation factor 1A, X-linked |
| 1430596_s_at | -6.78 | 0.00000 | VGLL3 | vestigial like 3 (Drosophila) |
| 1454877_at | -7.00 | 0.01872 | SERTAD4 | SERTA domain containing 4 |
| 1426528_at | -7.76 | 0.00000 | NRP2 | neuropilin 2 |
| 1453593_at | -8.04 | 0.00000 | VGLL3 | vestigial like 3 (Drosophila) |
| 1455299_at | -8.04 | 0.01872 | VGLL3 | vestigial like 3 (Drosophila) |
| 1456778_at | -8.68 | 0.01872 |  |  |
| 1416361_a_at | -9.23 | 0.01872 | DYNC1I1 | dynein, cytoplasmic 1, intermediate chain 1 |
| 1435349_at | -9.35 | 0.00000 | NRP2 | neuropilin 2 |
| 1422771_at | -10.75 | 0.00000 | SMAD6 | SMAD family member 6 |
| 1435106_at | -13.44 | 0.00000 | LIMCH1 | LIM and calponin homology domains 1 |
