# Supplementary material for: Tumor Endothelial Inflammation Predicts Clinical Outcome in Diverse Human Cancers
Source: PLoS One. 2012 Oct 4;7(10):e46104. doi: 10.1371/journal.pone.0046104 (PMC3464251; doi:10.1371/journal.pone.0046104)
Supplement: Table S4 — Differential expression of human orthologs of tumor endothelium-derived genes in datasets of chronic inflammatory diseases. Expression indicates the direction of gene expression in the experimental model (WT/KO TAECs) with UP signifying up-regulation and DOWN signifying down-regulation. Inflammatory bowel disease, IBD. Rheumatoid arthritis, RA. Cirrhosis, CIR. Genes with differential expression in diseased samples compared to normal tissue controls are indicated by a “1”. The 49 genes designated as mutually dysregulated and concordant in expression with the experimental model are indicated in the final column. (DOC) [file pone.0046104.s010.doc]

| **Gene Symbol** | **Expression** | **IBD** | **RA** | **CIR** | **49-gene set** |
| --- | --- | --- | --- | --- | --- |
| ACTG2 | UP | 1 | 0 | 0 | 0 |
| ACTR3B | UP | 0 | 0 | 0 | 0 |
| ADAM12 | DOWN | 1 | 0 | 0 | 0 |
| ADAR | UP | 0 | 0 | 0 | 0 |
| ADD3 | DOWN | 0 | 1 | 1 | 0 |
| AFAP1L2 | UP | 1 | 0 | 0 | 0 |
| AGFG2 | UP | 0 | 0 | 1 | 0 |
| AHNAK2 | UP | 0 | 0 | 1 | 0 |
| AHSA1 | UP | 0 | 0 | 0 | 0 |
| AIF1L | DOWN | 0 | 0 | 0 | 0 |
| AK3L1 | UP | 0 | 0 | 1 | 0 |
| AKAP12 | UP | 1 | 0 | 1 | 1 |
| AKAP13 | UP | 0 | 1 | 1 | 0 |
| AKAP6 | DOWN | 0 | 0 | 0 | 0 |
| ALCAM | DOWN | 0 | 1 | 0 | 0 |
| ALG10 | DOWN | 0 | 0 | 0 | 0 |
| AMN1 | UP | 0 | 0 | 0 | 0 |
| AMOTL2 | DOWN | 1 | 0 | 1 | 0 |
| ANKRD2 | DOWN | 0 | 0 | 0 | 0 |
| ANKRD37 | UP | 0 | 0 | 0 | 0 |
| ANXA3 | UP | 1 | 0 | 1 | 1 |
| AOX1 | UP | 1 | 0 | 1 | 0 |
| APPBP2 | UP | 0 | 0 | 0 | 0 |
| AQP1 | UP | 1 | 0 | 1 | 1 |
| ARFGEF2 | DOWN | 0 | 0 | 0 | 0 |
| ARHGAP26 | UP | 0 | 0 | 0 | 0 |
| ARHGEF37 | DOWN | 0 | 0 | 0 | 0 |
| ARID2 | DOWN | 0 | 0 | 0 | 0 |
| ARID4B | DOWN | 0 | 0 | 0 | 0 |
| ARID5B | UP | 0 | 0 | 1 | 0 |
| ARPC2 | UP | 0 | 0 | 1 | 0 |
| ASAP1 | UP | 1 | 0 | 1 | 0 |
| ASCC3 | UP | 0 | 1 | 0 | 0 |
| ATOX1 | UP | 1 | 0 | 0 | 0 |
| AUH | UP | 0 | 0 | 0 | 0 |
| AXL | UP | 0 | 0 | 1 | 0 |
| BAG4 | DOWN | 0 | 0 | 0 | 0 |
| BCAN | DOWN | 0 | 0 | 0 | 0 |
| BCL2L11 | UP | 0 | 0 | 0 | 0 |
| BGN | UP | 1 | 0 | 1 | 1 |
| BID | UP | 0 | 0 | 0 | 0 |
| BOP1 | UP | 0 | 1 | 1 | 0 |
| BST2 | UP | 1 | 0 | 1 | 1 |
| C12orf28 | UP | 0 | 0 | 0 | 0 |
| C12orf48 | DOWN | 0 | 0 | 0 | 0 |
| C14orf153 | UP | 0 | 0 | 0 | 0 |
| C19orf42 | DOWN | 0 | 0 | 0 | 0 |
| C2 | UP | 1 | 0 | 0 | 0 |
| C2orf43 | UP | 0 | 0 | 1 | 0 |
| C5orf15 | UP | 1 | 0 | 0 | 0 |
| C5orf24 | DOWN | 0 | 0 | 0 | 0 |
| C5orf34 | UP | 0 | 0 | 0 | 0 |
| C9orf125 | UP | 0 | 0 | 0 | 0 |
| C9orf140 | DOWN | 0 | 0 | 0 | 0 |
| C9orf91 | DOWN | 0 | 0 | 0 | 0 |
| CAND1 | DOWN | 0 | 1 | 0 | 0 |
| CBLB | UP | 1 | 1 | 1 | 0 |
| CCDC102A | UP | 1 | 0 | 0 | 0 |
| CCDC58 | UP | 0 | 0 | 0 | 0 |
| CCDC76 | UP | 0 | 0 | 0 | 0 |
| CCK | UP | 0 | 1 | 0 | 0 |
| CCL13 | UP | 0 | 1 | 0 | 0 |
| CCNG2 | UP | 1 | 0 | 1 | 1 |
| CD38 | UP | 1 | 1 | 0 | 0 |
| CD74 | UP | 1 | 0 | 1 | 1 |
| CD93 | UP | 1 | 0 | 1 | 1 |
| CDC42BPG | UP | 0 | 0 | 0 | 0 |
| CDC73 | DOWN | 0 | 0 | 0 | 0 |
| CDCA2 | DOWN | 0 | 0 | 0 | 0 |
| CDKN1A | UP | 0 | 1 | 0 | 0 |
| CEP350 | DOWN | 0 | 0 | 0 | 0 |
| CFL2 | UP | 1 | 0 | 0 | 0 |
| CFTR | DOWN | 1 | 0 | 1 | 0 |
| CHCHD3 | UP | 0 | 0 | 0 | 0 |
| CHD6 | UP | 0 | 0 | 0 | 0 |
| CKAP2L | DOWN | 0 | 0 | 0 | 0 |
| CKLF | UP | 1 | 0 | 1 | 1 |
| CLTA | UP | 0 | 0 | 0 | 0 |
| CLU | UP | 1 | 0 | 1 | 0 |
| CMPK2 | UP | 0 | 0 | 0 | 0 |
| CNP | UP | 0 | 0 | 0 | 0 |
| COL1A2 | UP | 1 | 0 | 1 | 1 |
| COL4A2 | UP | 1 | 0 | 1 | 1 |
| COL4A4 | DOWN | 1 | 0 | 0 | 0 |
| COL5A1 | UP | 1 | 0 | 1 | 1 |
| COL6A1 | UP | 1 | 1 | 1 | 1 |
| COL6A3 | UP | 1 | 0 | 1 | 1 |
| COL9A1 | DOWN | 0 | 0 | 0 | 0 |
| COLEC11 | UP | 0 | 0 | 1 | 0 |
| CORO7 | DOWN | 0 | 0 | 0 | 0 |
| CPEB2 | DOWN | 0 | 0 | 0 | 0 |
| CPNE4 | DOWN | 0 | 0 | 0 | 0 |
| CRMP1 | UP | 0 | 0 | 0 | 0 |
| CSF1 | UP | 0 | 1 | 0 | 0 |
| CSNK1A1 | UP | 0 | 0 | 0 | 0 |
| CTNNA1 | UP | 0 | 0 | 0 | 0 |
| CTSO | UP | 0 | 0 | 1 | 0 |
| CUX1 | UP | 0 | 0 | 0 | 0 |
| CWC22 | UP | 0 | 0 | 0 | 0 |
| CXCL10 | UP | 0 | 1 | 1 | 1 |
| CXCL2 | UP | 1 | 0 | 0 | 0 |
| CXCL3 | UP | 1 | 0 | 0 | 0 |
| CYR61 | DOWN | 1 | 1 | 1 | 0 |
| DBP | UP | 0 | 1 | 0 | 0 |
| DCAF7 | UP | 0 | 1 | 0 | 0 |
| DCUN1D1 | UP | 0 | 0 | 0 | 0 |
| DCUN1D5 | DOWN | 0 | 0 | 0 | 0 |
| DDIT4 | UP | 1 | 0 | 1 | 1 |
| DDX23 | UP | 0 | 1 | 0 | 0 |
| DDX24 | UP | 0 | 1 | 0 | 0 |
| DDX58 | UP | 0 | 0 | 1 | 0 |
| DDX60 | UP | 0 | 1 | 1 | 1 |
| DEPDC1B | DOWN | 0 | 0 | 0 | 0 |
| DEPDC6 | DOWN | 0 | 0 | 1 | 0 |
| DHODH | UP | 0 | 0 | 1 | 0 |
| DHX58 | UP | 0 | 0 | 0 | 0 |
| DMTF1 | UP | 0 | 0 | 1 | 0 |
| DNAJA2 | UP | 0 | 0 | 0 | 0 |
| DNAJB14 | DOWN | 0 | 0 | 0 | 0 |
| DNAJC3 | DOWN | 1 | 0 | 1 | 0 |
| DNM1 | DOWN | 0 | 0 | 0 | 0 |
| DOCK4 | UP | 1 | 0 | 0 | 0 |
| DOCK5 | DOWN | 0 | 0 | 0 | 0 |
| DPH3 | DOWN | 0 | 0 | 0 | 0 |
| DSTYK | DOWN | 0 | 0 | 0 | 0 |
| DTX3L | UP | 0 | 0 | 0 | 0 |
| DUS4L | UP | 0 | 0 | 0 | 0 |
| DUSP4 | DOWN | 1 | 0 | 1 | 0 |
| DYNC1I1 | DOWN | 0 | 0 | 0 | 0 |
| DYRK1A | DOWN | 0 | 0 | 0 | 0 |
| DZIP3 | UP | 1 | 0 | 0 | 0 |
| EIF1AX | DOWN | 0 | 0 | 1 | 0 |
| EIF2AK2 | UP | 0 | 0 | 0 | 0 |
| EIF4EBP2 | DOWN | 0 | 1 | 0 | 0 |
| EIF4H | UP | 0 | 0 | 0 | 0 |
| ELAC1 | DOWN | 0 | 0 | 0 | 0 |
| ELAVL1 | DOWN | 0 | 0 | 0 | 0 |
| ELK3 | DOWN | 1 | 0 | 1 | 0 |
| EMCN | UP | 1 | 0 | 1 | 0 |
| EML2 | UP | 0 | 1 | 0 | 0 |
| ENDOD1 | UP | 0 | 0 | 1 | 0 |
| ENPEP | UP | 0 | 0 | 0 | 0 |
| EPB41L4B | DOWN | 1 | 0 | 0 | 0 |
| EPHA4 | DOWN | 0 | 0 | 0 | 0 |
| ERAP1 | UP | 0 | 0 | 1 | 0 |
| ERCC8 | DOWN | 0 | 0 | 0 | 0 |
| ETV3 | UP | 0 | 0 | 0 | 0 |
| ETV5 | DOWN | 0 | 1 | 0 | 0 |
| EXTL3 | DOWN | 0 | 1 | 0 | 0 |
| EYA1 | DOWN | 0 | 0 | 0 | 0 |
| F2R | UP | 1 | 0 | 1 | 1 |
| FABP4 | UP | 0 | 0 | 1 | 0 |
| FAM104A | DOWN | 0 | 0 | 0 | 0 |
| FAM134C | UP | 0 | 0 | 0 | 0 |
| FAM162A | UP | 1 | 0 | 0 | 0 |
| FAM176B | UP | 0 | 0 | 0 | 0 |
| FAM196A | UP | 0 | 0 | 0 | 0 |
| FAM199X | DOWN | 0 | 0 | 0 | 0 |
| FAM46A | UP | 0 | 0 | 0 | 0 |
| FAM65B | UP | 1 | 0 | 0 | 0 |
| FAM73A | DOWN | 0 | 0 | 0 | 0 |
| FANCF | UP | 0 | 1 | 0 | 0 |
| FBXW11 | UP | 0 | 1 | 0 | 0 |
| FCGR2B | UP | 1 | 1 | 1 | 1 |
| FERMT3 | UP | 1 | 0 | 0 | 0 |
| FGFR3 | DOWN | 1 | 0 | 0 | 0 |
| FIP1L1 | UP | 0 | 0 | 0 | 0 |
| FKBP10 | UP | 0 | 0 | 0 | 0 |
| FOS | DOWN | 0 | 1 | 1 | 0 |
| FSCN1 | DOWN | 0 | 0 | 0 | 0 |
| FSTL1 | UP | 1 | 0 | 1 | 1 |
| FTSJD2 | UP | 0 | 0 | 0 | 0 |
| FZD6 | DOWN | 0 | 0 | 1 | 0 |
| GABRA1 | UP | 0 | 0 | 0 | 0 |
| GBP2 | UP | 1 | 0 | 0 | 0 |
| GBP4 | UP | 0 | 0 | 0 | 0 |
| GBP6 | UP | 0 | 0 | 0 | 0 |
| GGNBP2 | UP | 0 | 0 | 0 | 0 |
| GIMAP4 | UP | 1 | 1 | 1 | 1 |
| GJA3 | DOWN | 0 | 0 | 0 | 0 |
| GLI3 | DOWN | 0 | 0 | 0 | 0 |
| GLS | DOWN | 0 | 1 | 1 | 0 |
| GNAQ | DOWN | 0 | 0 | 0 | 0 |
| GNG4 | DOWN | 0 | 0 | 0 | 0 |
| GOLGA6L4 | UP | 0 | 0 | 0 | 0 |
| GPC6 | DOWN | 1 | 0 | 0 | 0 |
| HAP1 | UP | 0 | 1 | 0 | 0 |
| HELZ | DOWN | 0 | 0 | 0 | 0 |
| HERC2 | UP | 0 | 0 | 0 | 0 |
| HERC4 | UP | 0 | 0 | 0 | 0 |
| HERC6 | UP | 0 | 1 | 1 | 1 |
| HGSNAT | DOWN | 0 | 0 | 0 | 0 |
| HINT3 | DOWN | 0 | 0 | 0 | 0 |
| HIPK3 | DOWN | 0 | 0 | 0 | 0 |
| HIST1H2AB | UP | 0 | 0 | 0 | 0 |
| HIST1H2AD | UP | 0 | 0 | 0 | 0 |
| HIST1H2AE | UP | 0 | 0 | 0 | 0 |
| HIST1H3A | UP | 0 | 1 | 0 | 0 |
| HIST1H3B | UP | 0 | 0 | 0 | 0 |
| HIST1H3C | UP | 0 | 0 | 0 | 0 |
| HIST1H3E | UP | 0 | 0 | 0 | 0 |
| HIST1H3F | UP | 0 | 0 | 0 | 0 |
| HIST1H3G | UP | 0 | 0 | 0 | 0 |
| HIST1H3H | UP | 0 | 0 | 0 | 0 |
| HIST1H3I | UP | 0 | 0 | 0 | 0 |
| HIST1H3J | UP | 0 | 0 | 0 | 0 |
| HIST3H2A | UP | 0 | 0 | 0 | 0 |
| HLA-B | UP | 0 | 0 | 1 | 0 |
| HLA-DQA1 | UP | 1 | 1 | 1 | 0 |
| HLA-E | UP | 1 | 0 | 1 | 1 |
| HLA-G | UP | 0 | 0 | 1 | 0 |
| HMBOX1 | UP | 0 | 0 | 0 | 0 |
| HMGA2 | DOWN | 0 | 0 | 0 | 0 |
| HMGCS1 | UP | 0 | 0 | 1 | 0 |
| HMMR | DOWN | 0 | 1 | 0 | 0 |
| HOOK3 | DOWN | 1 | 0 | 0 | 0 |
| HOXA11 | DOWN | 0 | 0 | 0 | 0 |
| HOXD13 | DOWN | 0 | 0 | 0 | 0 |
| HPCAL1 | UP | 0 | 0 | 0 | 0 |
| HPGDS | DOWN | 0 | 1 | 0 | 0 |
| HS2ST1 | DOWN | 0 | 0 | 1 | 0 |
| HSPA1A | UP | 0 | 1 | 1 | 0 |
| ID1 | DOWN | 0 | 0 | 0 | 0 |
| ID2 | DOWN | 0 | 1 | 0 | 0 |
| ID3 | DOWN | 0 | 0 | 1 | 0 |
| IER3 | UP | 1 | 1 | 1 | 0 |
| IFFO1 | DOWN | 1 | 0 | 0 | 0 |
| IFI35 | UP | 0 | 0 | 1 | 0 |
| IFI44 | UP | 1 | 0 | 1 | 1 |
| IFIH1 | UP | 0 | 0 | 1 | 0 |
| IFIT1 | UP | 0 | 0 | 1 | 0 |
| IFIT3 | UP | 0 | 0 | 1 | 0 |
| IFITM3 | UP | 1 | 0 | 0 | 0 |
| IGFBP3 | UP | 0 | 0 | 1 | 0 |
| IGFBP7 | UP | 1 | 0 | 1 | 1 |
| IL12RB1 | UP | 1 | 0 | 0 | 0 |
| IMPAD1 | DOWN | 0 | 0 | 0 | 0 |
| IPMK | DOWN | 0 | 0 | 0 | 0 |
| IRF1 | UP | 0 | 0 | 0 | 0 |
| IRF7 | UP | 1 | 0 | 1 | 1 |
| IRF9 | UP | 1 | 0 | 0 | 0 |
| IRGM | UP | 0 | 0 | 0 | 0 |
| ISG15 | UP | 1 | 0 | 1 | 1 |
| ITCH | UP | 0 | 0 | 0 | 0 |
| ITGA4 | DOWN | 1 | 1 | 1 | 0 |
| IVNS1ABP | DOWN | 0 | 0 | 0 | 0 |
| JKAMP | UP | 0 | 0 | 0 | 0 |
| KBTBD11 | UP | 0 | 0 | 0 | 0 |
| KBTBD8 | DOWN | 1 | 0 | 0 | 0 |
| KCNN4 | UP | 1 | 0 | 0 | 0 |
| KDM2A | UP | 1 | 0 | 0 | 0 |
| KHNYN | UP | 0 | 0 | 0 | 0 |
| KIAA0368 | UP | 0 | 0 | 0 | 0 |
| KIAA1267 | DOWN | 0 | 0 | 0 | 0 |
| KIAA1370 | UP | 1 | 0 | 0 | 0 |
| KIF6 | UP | 0 | 0 | 0 | 0 |
| KIT | DOWN | 0 | 0 | 1 | 0 |
| KLF4 | DOWN | 0 | 1 | 1 | 0 |
| KLHL21 | DOWN | 0 | 0 | 0 | 0 |
| LATS1 | DOWN | 0 | 0 | 0 | 0 |
| LATS2 | DOWN | 1 | 0 | 0 | 0 |
| LCK | UP | 1 | 1 | 1 | 1 |
| LGALS3BP | UP | 0 | 0 | 1 | 0 |
| LIFR | UP | 1 | 0 | 0 | 0 |
| LIMCH1 | DOWN | 0 | 0 | 1 | 0 |
| LIMD2 | UP | 0 | 0 | 0 | 0 |
| LIN7C | DOWN | 0 | 0 | 0 | 0 |
| LMAN1 | UP | 1 | 0 | 1 | 0 |
| LPIN1 | UP | 1 | 0 | 1 | 0 |
| LRBA | UP | 0 | 0 | 0 | 0 |
| LRRCC1 | DOWN | 0 | 0 | 0 | 0 |
| MAP3K5 | DOWN | 1 | 0 | 0 | 0 |
| MAP4 | UP | 0 | 1 | 0 | 0 |
| MAPRE1 | DOWN | 0 | 0 | 1 | 0 |
| MBNL2 | UP | 1 | 0 | 0 | 0 |
| MDH1 | UP | 0 | 0 | 0 | 0 |
| MED1 | UP | 0 | 1 | 0 | 0 |
| MED14 | DOWN | 0 | 0 | 0 | 0 |
| MEIS2 | UP | 1 | 0 | 0 | 0 |
| MEX3D | DOWN | 0 | 0 | 0 | 0 |
| MGAT4A | DOWN | 0 | 0 | 0 | 0 |
| MGP | UP | 1 | 1 | 1 | 0 |
| MIER1 | DOWN | 0 | 0 | 0 | 0 |
| MITD1 | UP | 0 | 0 | 0 | 0 |
| MLKL | UP | 1 | 0 | 0 | 0 |
| MLPH | DOWN | 0 | 0 | 0 | 0 |
| MMGT1 | DOWN | 0 | 0 | 0 | 0 |
| MOV10 | UP | 0 | 0 | 0 | 0 |
| MRPL47 | DOWN | 0 | 0 | 0 | 0 |
| MSRB3 | DOWN | 1 | 0 | 0 | 0 |
| MTSS1 | UP | 0 | 1 | 0 | 0 |
| MXD1 | DOWN | 0 | 1 | 0 | 0 |
| MYO1E | DOWN | 0 | 0 | 0 | 0 |
| NAP1L1 | DOWN | 1 | 0 | 1 | 0 |
| NCALD | DOWN | 0 | 0 | 0 | 0 |
| NDRG1 | DOWN | 0 | 0 | 1 | 0 |
| NEK6 | DOWN | 0 | 0 | 0 | 0 |
| NEO1 | UP | 0 | 0 | 0 | 0 |
| NFIX | UP | 0 | 1 | 0 | 0 |
| NFKBIA | UP | 0 | 0 | 1 | 0 |
| NFKBIE | UP | 1 | 1 | 0 | 1 |
| NFKBIZ | UP | 1 | 0 | 0 | 0 |
| NMI | UP | 0 | 0 | 0 | 0 |
| NOS3 | UP | 0 | 0 | 0 | 0 |
| NR4A2 | DOWN | 0 | 1 | 1 | 0 |
| NR4A3 | DOWN | 1 | 1 | 1 | 0 |
| NRCAM | UP | 0 | 0 | 0 | 0 |
| NRP2 | DOWN | 1 | 1 | 0 | 0 |
| NUS1 | DOWN | 0 | 0 | 1 | 0 |
| OAS1 | UP | 0 | 0 | 1 | 0 |
| OCRL | UP | 0 | 0 | 0 | 0 |
| OGFR | UP | 0 | 1 | 0 | 0 |
| OLFM1 | UP | 1 | 1 | 0 | 0 |
| OPTN | DOWN | 0 | 1 | 0 | 0 |
| OSTC | UP | 0 | 0 | 0 | 0 |
| OTUD7B | DOWN | 0 | 0 | 0 | 0 |
| P4HA2 | UP | 0 | 0 | 0 | 0 |
| PABPC1 | UP | 0 | 0 | 0 | 0 |
| PADI2 | DOWN | 1 | 0 | 0 | 0 |
| PAK3 | DOWN | 0 | 0 | 0 | 0 |
| PARP10 | UP | 0 | 0 | 0 | 0 |
| PARP12 | UP | 0 | 0 | 0 | 0 |
| PARP14 | UP | 1 | 0 | 0 | 0 |
| PARP3 | UP | 0 | 0 | 0 | 0 |
| PARP9 | UP | 0 | 0 | 0 | 0 |
| PCDH9 | UP | 0 | 1 | 0 | 0 |
| PDDC1 | UP | 0 | 0 | 0 | 0 |
| PDE4B | UP | 1 | 1 | 1 | 0 |
| PDK1 | UP | 1 | 1 | 0 | 1 |
| PDZRN3 | DOWN | 0 | 0 | 1 | 0 |
| PELI1 | UP | 0 | 0 | 0 | 0 |
| PEX11A | DOWN | 0 | 1 | 0 | 0 |
| PHYHIPL | UP | 0 | 0 | 0 | 0 |
| PIK3IP1 | UP | 1 | 0 | 1 | 1 |
| PIKFYVE | DOWN | 1 | 0 | 0 | 0 |
| PKN1 | UP | 0 | 0 | 0 | 0 |
| PLA2G15 | UP | 0 | 0 | 0 | 0 |
| PLAT | UP | 0 | 0 | 1 | 0 |
| PLCB4 | UP | 0 | 0 | 0 | 0 |
| PLEC | UP | 0 | 1 | 0 | 0 |
| PLEKHA2 | UP | 1 | 0 | 0 | 0 |
| PLVAP | UP | 1 | 0 | 1 | 1 |
| PMEPA1 | DOWN | 1 | 0 | 0 | 0 |
| PML | UP | 1 | 1 | 0 | 0 |
| PNRC1 | UP | 0 | 1 | 0 | 0 |
| PPAP2B | UP | 0 | 1 | 0 | 0 |
| PPFIBP2 | DOWN | 0 | 0 | 0 | 0 |
| PQLC3 | UP | 1 | 0 | 0 | 0 |
| PREB | DOWN | 0 | 0 | 0 | 0 |
| PREP | DOWN | 0 | 0 | 0 | 0 |
| PRIC285 | UP | 0 | 0 | 0 | 0 |
| PRKAG2 | DOWN | 0 | 0 | 0 | 0 |
| PRKAR2A | DOWN | 0 | 1 | 1 | 0 |
| PRKG2 | DOWN | 1 | 0 | 0 | 0 |
| PRPF4B | DOWN | 0 | 1 | 0 | 0 |
| PSMA1 | UP | 0 | 0 | 0 | 0 |
| PSMB10 | UP | 0 | 1 | 1 | 1 |
| PSMB8 | UP | 1 | 1 | 1 | 1 |
| PSMB9 | UP | 1 | 1 | 1 | 1 |
| PTGER1 | UP | 0 | 1 | 1 | 0 |
| PTPLB | DOWN | 0 | 0 | 0 | 0 |
| PTPN6 | UP | 1 | 0 | 0 | 0 |
| PTTG1 | UP | 0 | 1 | 0 | 0 |
| PUS3 | UP | 0 | 0 | 0 | 0 |
| RAB14 | UP | 0 | 0 | 1 | 0 |
| RAB27A | DOWN | 0 | 0 | 1 | 0 |
| RAB3IP | DOWN | 0 | 0 | 0 | 0 |
| RAB7L1 | UP | 1 | 0 | 1 | 0 |
| RAD23B | UP | 0 | 0 | 0 | 0 |
| RAI14 | UP | 1 | 0 | 0 | 0 |
| RAMP2 | UP | 0 | 0 | 0 | 0 |
| RAP2A | DOWN | 0 | 1 | 0 | 0 |
| RAPGEF1 | DOWN | 0 | 1 | 0 | 0 |
| RAPGEF5 | UP | 0 | 0 | 1 | 0 |
| RBM28 | DOWN | 0 | 0 | 0 | 0 |
| RBMX | UP | 0 | 0 | 0 | 0 |
| RDH14 | UP | 0 | 0 | 0 | 0 |
| RELB | UP | 0 | 0 | 0 | 0 |
| RFX5 | UP | 1 | 1 | 1 | 1 |
| RGMB | UP | 0 | 0 | 0 | 0 |
| RGS16 | UP | 1 | 1 | 0 | 0 |
| RGS2 | UP | 1 | 0 | 1 | 1 |
| RGS5 | UP | 1 | 0 | 1 | 1 |
| RHOD | DOWN | 0 | 0 | 1 | 0 |
| RIT1 | DOWN | 1 | 0 | 1 | 0 |
| RLIM | DOWN | 0 | 0 | 0 | 0 |
| RMND5A | DOWN | 1 | 0 | 0 | 0 |
| RNASEL | UP | 0 | 0 | 0 | 0 |
| RND3 | DOWN | 0 | 0 | 1 | 0 |
| RNF213 | UP | 1 | 0 | 0 | 0 |
| RPS20 | DOWN | 0 | 0 | 0 | 0 |
| RSAD2 | UP | 0 | 0 | 1 | 0 |
| RTN4 | UP | 0 | 0 | 1 | 0 |
| RTP4 | UP | 0 | 1 | 1 | 1 |
| RUNX2 | DOWN | 1 | 0 | 0 | 0 |
| RUSC2 | DOWN | 0 | 0 | 0 | 0 |
| S100A10 | UP | 0 | 0 | 1 | 0 |
| S100A4 | DOWN | 1 | 0 | 1 | 0 |
| SAMD9L | UP | 1 | 0 | 0 | 0 |
| SAMHD1 | UP | 1 | 0 | 0 | 0 |
| SAT1 | DOWN | 0 | 1 | 0 | 0 |
| SBF2 | UP | 0 | 0 | 0 | 0 |
| SDC4 | UP | 0 | 0 | 0 | 0 |
| SDHA | UP | 0 | 0 | 0 | 0 |
| SDPR | DOWN | 1 | 0 | 1 | 0 |
| SENP1 | DOWN | 0 | 0 | 0 | 0 |
| SENP6 | UP | 0 | 0 | 1 | 0 |
| SEPSECS | DOWN | 0 | 0 | 0 | 0 |
| SEPW1 | DOWN | 0 | 0 | 0 | 0 |
| SERBP1 | DOWN | 0 | 0 | 0 | 0 |
| SERPINB8 | DOWN | 0 | 0 | 0 | 0 |
| SERPINB9 | UP | 1 | 0 | 1 | 1 |
| SERTAD4 | DOWN | 0 | 0 | 0 | 0 |
| SETD3 | UP | 0 | 0 | 0 | 0 |
| SETD7 | DOWN | 0 | 0 | 0 | 0 |
| SF3B3 | UP | 0 | 0 | 0 | 0 |
| SFI1 | UP | 0 | 1 | 0 | 0 |
| SFRS12IP1 | DOWN | 0 | 0 | 0 | 0 |
| SFRS18 | DOWN | 0 | 1 | 1 | 0 |
| SFRS7 | DOWN | 0 | 1 | 1 | 0 |
| SGIP1 | UP | 1 | 0 | 0 | 0 |
| SH3PXD2A | DOWN | 0 | 1 | 0 | 0 |
| SH3PXD2B | DOWN | 1 | 0 | 0 | 0 |
| SH3RF1 | UP | 0 | 0 | 0 | 0 |
| SHISA5 | UP | 0 | 0 | 0 | 0 |
| SHROOM2 | DOWN | 0 | 0 | 0 | 0 |
| SKIL | DOWN | 0 | 0 | 0 | 0 |
| SLBP | UP | 0 | 0 | 0 | 0 |
| SLC12A5 | UP | 0 | 0 | 0 | 0 |
| SLC26A7 | DOWN | 0 | 0 | 0 | 0 |
| SLC2A1 | UP | 0 | 0 | 0 | 0 |
| SLC35A3 | DOWN | 0 | 0 | 1 | 0 |
| SLC35F1 | DOWN | 0 | 0 | 0 | 0 |
| SLC40A1 | UP | 1 | 0 | 0 | 0 |
| SLC9A8 | UP | 0 | 0 | 1 | 0 |
| SMAD6 | DOWN | 0 | 1 | 0 | 0 |
| SMAD7 | DOWN | 0 | 0 | 1 | 0 |
| SMG1 | UP | 0 | 1 | 0 | 0 |
| SMPD4 | DOWN | 0 | 0 | 0 | 0 |
| SNCA | DOWN | 0 | 1 | 0 | 0 |
| SOCS6 | DOWN | 0 | 0 | 1 | 0 |
| SORBS1 | DOWN | 0 | 1 | 0 | 0 |
| SOX10 | DOWN | 0 | 0 | 0 | 0 |
| SOX7 | UP | 1 | 0 | 0 | 0 |
| SPAG9 | UP | 0 | 1 | 0 | 0 |
| SPHK1 | DOWN | 1 | 0 | 0 | 0 |
| SPP1 | UP | 1 | 0 | 1 | 1 |
| SRL | UP | 0 | 0 | 0 | 0 |
| SRRM1 | UP | 0 | 1 | 0 | 0 |
| SRRM2 | UP | 0 | 1 | 0 | 0 |
| SSPN | UP | 0 | 0 | 1 | 0 |
| SSR1 | DOWN | 1 | 0 | 1 | 0 |
| ST3GAL4 | DOWN | 0 | 0 | 0 | 0 |
| ST8SIA4 | UP | 1 | 0 | 0 | 0 |
| STARD9 | UP | 0 | 0 | 0 | 0 |
| STAT1 | UP | 1 | 1 | 1 | 1 |
| STAT2 | UP | 0 | 1 | 0 | 0 |
| STX3 | DOWN | 0 | 1 | 0 | 0 |
| STXBP5 | DOWN | 0 | 0 | 0 | 0 |
| SUPT16H | UP | 0 | 0 | 0 | 0 |
| SYN2 | DOWN | 0 | 0 | 0 | 0 |
| SYNE2 | UP | 0 | 0 | 0 | 0 |
| SYNM | DOWN | 1 | 0 | 0 | 0 |
| TACC1 | DOWN | 0 | 0 | 1 | 0 |
| TAF10 | DOWN | 0 | 0 | 0 | 0 |
| TAF1D | DOWN | 0 | 0 | 1 | 0 |
| TAOK3 | UP | 0 | 0 | 1 | 0 |
| TAP1 | UP | 1 | 1 | 1 | 1 |
| TAPBP | UP | 1 | 1 | 0 | 1 |
| TARDBP | DOWN | 0 | 0 | 0 | 0 |
| TBC1D1 | UP | 1 | 0 | 0 | 0 |
| TBC1D16 | DOWN | 0 | 0 | 1 | 0 |
| TCP11L2 | DOWN | 0 | 0 | 0 | 0 |
| TDRD7 | UP | 0 | 0 | 1 | 0 |
| TEF | UP | 0 | 0 | 0 | 0 |
| TES | DOWN | 0 | 0 | 0 | 0 |
| TET1 | UP | 0 | 0 | 0 | 0 |
| TFAP2A | DOWN | 0 | 1 | 0 | 0 |
| TGFA | DOWN | 0 | 0 | 1 | 0 |
| THBS1 | UP | 1 | 1 | 1 | 0 |
| TIAL1 | DOWN | 0 | 0 | 0 | 0 |
| TLK2 | UP | 0 | 0 | 0 | 0 |
| TLR3 | UP | 0 | 1 | 0 | 0 |
| TM4SF1 | DOWN | 1 | 1 | 1 | 0 |
| TM9SF3 | DOWN | 0 | 0 | 0 | 0 |
| TMCC2 | DOWN | 0 | 1 | 0 | 0 |
| TMCC3 | DOWN | 0 | 0 | 0 | 0 |
| TMEM106A | UP | 0 | 0 | 0 | 0 |
| TMEM140 | UP | 0 | 0 | 1 | 0 |
| TMEM144 | DOWN | 1 | 0 | 0 | 0 |
| TMEM204 | UP | 1 | 0 | 1 | 1 |
| TMEM33 | DOWN | 0 | 0 | 1 | 0 |
| TMEM50B | DOWN | 0 | 0 | 0 | 0 |
| TMEM56 | DOWN | 0 | 0 | 0 | 0 |
| TMEM64 | DOWN | 1 | 0 | 0 | 0 |
| TMOD3 | UP | 0 | 0 | 0 | 0 |
| TMPO | DOWN | 0 | 1 | 0 | 0 |
| TNC | UP | 1 | 1 | 1 | 1 |
| TNFAIP3 | UP | 1 | 0 | 1 | 1 |
| TNNT2 | DOWN | 0 | 0 | 0 | 0 |
| TOR3A | UP | 1 | 0 | 0 | 0 |
| TPX2 | DOWN | 0 | 0 | 0 | 0 |
| TREX1 | UP | 0 | 0 | 0 | 0 |
| TRIM21 | UP | 0 | 1 | 0 | 0 |
| TRIM25 | UP | 0 | 1 | 0 | 0 |
| TRIM41 | UP | 0 | 0 | 0 | 0 |
| TRIM5 | UP | 0 | 0 | 1 | 0 |
| TSPO | UP | 0 | 0 | 0 | 0 |
| TSPYL1 | DOWN | 0 | 0 | 0 | 0 |
| TUBB3 | UP | 0 | 0 | 0 | 0 |
| TULP3 | DOWN | 0 | 0 | 0 | 0 |
| TXNDC9 | DOWN | 0 | 0 | 0 | 0 |
| TXNRD1 | DOWN | 0 | 0 | 0 | 0 |
| UBA7 | UP | 1 | 0 | 0 | 0 |
| UBE2I | UP | 0 | 1 | 0 | 0 |
| UBE2L6 | UP | 1 | 1 | 1 | 1 |
| UBE2N | DOWN | 0 | 0 | 0 | 0 |
| UBFD1 | DOWN | 0 | 0 | 1 | 0 |
| UPP1 | UP | 0 | 0 | 0 | 0 |
| URM1 | DOWN | 0 | 0 | 0 | 0 |
| USF2 | DOWN | 0 | 0 | 1 | 0 |
| USP11 | UP | 0 | 0 | 0 | 0 |
| USP18 | UP | 0 | 0 | 1 | 0 |
| USP53 | UP | 0 | 0 | 0 | 0 |
| USP9X | DOWN | 0 | 0 | 0 | 0 |
| VAMP2 | DOWN | 0 | 0 | 0 | 0 |
| VCPIP1 | UP | 0 | 0 | 0 | 0 |
| VGLL3 | DOWN | 1 | 0 | 0 | 0 |
| VPS37B | DOWN | 0 | 0 | 1 | 0 |
| VTI1A | UP | 0 | 0 | 0 | 0 |
| WDR90 | DOWN | 0 | 0 | 0 | 0 |
| WHSC1L1 | UP | 0 | 0 | 0 | 0 |
| WSB2 | DOWN | 0 | 1 | 1 | 1 |
| WWC2 | DOWN | 0 | 1 | 0 | 0 |
| YES1 | UP | 0 | 0 | 1 | 0 |
| YPEL2 | UP | 0 | 0 | 0 | 0 |
| ZBTB4 | UP | 1 | 0 | 0 | 0 |
| ZC3H15 | UP | 0 | 0 | 0 | 0 |
| ZDHHC14 | UP | 0 | 0 | 0 | 0 |
| ZDHHC15 | UP | 0 | 0 | 0 | 0 |
| ZFP106 | DOWN | 0 | 0 | 0 | 0 |
| ZFX | DOWN | 0 | 1 | 0 | 0 |
| ZIC5 | DOWN | 0 | 0 | 0 | 0 |
| ZKSCAN1 | UP | 0 | 0 | 0 | 0 |
| ZMIZ1 | UP | 0 | 0 | 0 | 0 |
| ZMYND8 | UP | 0 | 0 | 1 | 0 |
| ZNF287 | UP | 0 | 0 | 0 | 0 |
| ZNF292 | UP | 0 | 0 | 1 | 0 |
| ZNF295 | UP | 0 | 0 | 0 | 0 |
| ZNF397 | UP | 0 | 0 | 0 | 0 |
| ZNF467 | UP | 0 | 0 | 0 | 0 |
| ZNF704 | DOWN | 0 | 0 | 0 | 0 |
| ZNF808 | UP | 0 | 0 | 0 | 0 |
| ZNFX1 | UP | 0 | 0 | 0 | 0 |
