# Supplementary material for: Tumor Endothelial Inflammation Predicts Clinical Outcome in Diverse Human Cancers
Source: PLoS One. 2012 Oct 4;7(10):e46104. doi: 10.1371/journal.pone.0046104 (PMC3464251; doi:10.1371/journal.pone.0046104)
Supplement: Table S5 — Classification values obtained by hierarchical clustering of tumor endothelial-derived genes in human inflammatory disease datasets. PPV denotes positive predictive value, while NPV indicates negative predictive value. (DOC) [file pone.0046104.s011.doc]

| **Disease** | **Accuracy** | **Sensitivity** | **Specificity** | **PPV** | **NPV** |
| --- | --- | --- | --- | --- | --- |
| Cirrhosis | 100% | 100% | 100% | 100% | 100% |
| Inflammatory bowel disease | 89% | 88% | 90% | 88% | 90% |
| Rheumatoid arthritis | 95% | 92% | 100% | 100% | 90% |
