# Supplementary material for: Tumor Endothelial Inflammation Predicts Clinical Outcome in Diverse Human Cancers
Source: PLoS One. 2012 Oct 4;7(10):e46104. doi: 10.1371/journal.pone.0046104 (PMC3464251; doi:10.1371/journal.pone.0046104)
Supplement: Table S6 — Cox proportional hazard analysis of overall survival for 295 breast cancer patients. The indicated model effects were used in the analysis. Age was considered a continuous variable. All other factors were considered as binary variables. Factors significant on univariate analysis were entered into multivariate and interaction (with IREG+) analyses. Hazard ratio = HR. Confidence interval = CI. Lymph node, LN. (DOC) [file pone.0046104.s012.doc]

|  |  | **Univariate** |  |  |  | **Multivariate** |  |  | **Interaction** |
| --- | --- | --- | --- | --- | --- | --- | --- | --- | --- |
| *Covariate* | *HR* | *95% CI* | *P-value* |  | *HR* | *95% CI* | *P-value* |  | *P-value* |
| Age (per year) | 0.94 | (0.91, 0.98) | 0.005 |  | 0.95 | (0.92, 0.99) | 0.018 |  | 0.70 |
| Size ≥T2 vs. <T2 | 2.13 | (1.36, 3.41) | <0.001 |  | 1.62 | (1.01, 2.62) | 0.044 |  | 0.31 |
| LN (+) vs. (-) | 0.88 | (0.56, 1.36) | 0.56 |  |  |  |  |  |  |
| ER (-) vs. (+) | 3.28 | (2.09, 5.12) | <0.001 |  |  |  |  |  | 0.90 |
| Grade 3 vs. 1, 2 | 3.35 | (2.13, 5.38) | <0.001 |  | 2.37 | (1.44, 3.99) | <0.001 |  | 0.89 |
| IREG (+) vs. (-) | 2.37 | (1.49, 3.84) | <0.001 |  | 1.58 | (0.96, 2.65) | 0.070 |  |  |
