# Supplementary material for: Tumor Endothelial Inflammation Predicts Clinical Outcome in Diverse Human Cancers
Source: PLoS One. 2012 Oct 4;7(10):e46104. doi: 10.1371/journal.pone.0046104 (PMC3464251; doi:10.1371/journal.pone.0046104)
Supplement: Table S7 — Cox proportional hazard analysis of overall survival for 232 colon cancer patients. The indicated model effects were used in the analysis. Age was considered a continuous variable. Stage (1–4) was considered an ordinal variable. IREG status was considered a binary variable. Factors significant on univariate analysis were entered into multivariate and interaction (with IREG+) analyses. Hazard ratio = HR. Confidence interval = CI. (DOC) [file pone.0046104.s013.doc]

|  |  | **Univariate** |  |  |  | **Multivariate** |  |  | **Interaction** |
| --- | --- | --- | --- | --- | --- | --- | --- | --- | --- |
| *Covariate* | *HR* | *95% CI* | *P-value* |  | *HR* | *95% CI* | *P-value* |  | *P-value* |
| Age (per year) | 1.01 | (0.99-1.03) | 0.30 |  |  |  |  |  |  |
| Stage | 2.73 | (2.10, 3.58) | <0.001 |  | 2.62 | (2.02, 3.44) | <0.001 |  | 0.78 |
| Grade 3 vs. 1, 2 | 0.72 | (0.35, 1.32) | 0.31 |  |  |  |  |  |  |
| IREG (+) vs. (-) | 2.09 | (1.38, 3.22) | <0.001 |  | 1.76 | (1.16, 2.72) | 0.0082 |  |  |
