# Supplementary material for: Tumor Endothelial Inflammation Predicts Clinical Outcome in Diverse Human Cancers
Source: PLoS One. 2012 Oct 4;7(10):e46104. doi: 10.1371/journal.pone.0046104 (PMC3464251; doi:10.1371/journal.pone.0046104)
Supplement: Table S8 — Cox proportional hazard analysis of overall survival for 441 lung cancer patients. The indicated model effects were used in the analysis. Age was considered a continuous variable. All other factors were considered as binary variables. Factors significant on univariate analysis were entered into multivariate and interaction (with IREG+) analyses. Hazard ratio = HR. Confidence interval = CI. Lymph node, LN. (DOC) [file pone.0046104.s014.doc]

|  |  | **Univariate** |  |  |  | **Multivariate** |  |  | **Interaction** |
| --- | --- | --- | --- | --- | --- | --- | --- | --- | --- |
| *Covariate* | *HR* | *95% CI* | *P-value* |  | *HR* | *95% CI* | *P-value* |  | *P-value* |
| Age (per year) | 1.03 | (1.01, 1.04) | <0.001 |  | 1.03 | (1.02, 1.05) | <0.001 |  | 0.38 |
| LN (+) vs. (-) | 2.75 | (2.11, 3.56) | <0.001 |  | 2.89 | (2.22, 3.75) | <0.001 |  | 0.084 |
| Size ≥T3 vs. <T3 | 2.87 | (1.96, 4.09) | <0.001 |  | 2.37 | (1.61, 3.40) | <0.001 |  | 0.11 |
| Grade 3 vs. 1, 2 | 1.17 | (0.89, 1.51) | 0.25 |  |  |  |  |  |  |
| IREG (+) vs. (-) | 1.59 | (1.23, 2.08) | <0.001 |  | 1.53 | (1.17, 2.00) | 0.0015 |  |  |
