# Supplementary material for: Tumor Endothelial Inflammation Predicts Clinical Outcome in Diverse Human Cancers
Source: PLoS One. 2012 Oct 4;7(10):e46104. doi: 10.1371/journal.pone.0046104 (PMC3464251; doi:10.1371/journal.pone.0046104)
Supplement: Table S9 — Cox proportional hazard analysis of overall survival for 77 glioma patients. The indicated model effects were used in the analysis. Age was considered a continuous variable. IREG status was considered a binary variable. Factors significant on univariate analysis were entered into multivariate and interaction (with IREG+) analyses. Hazard ratio = HR. Confidence interval = CI. (DOC) [file pone.0046104.s015.doc]

|  |  | **Univariate** |  |  |  | **Multivariate** |  |  | **Interaction** |
| --- | --- | --- | --- | --- | --- | --- | --- | --- | --- |
| *Covariate* | *HR* | *95% CI* | *P-value* |  | *HR* | *95% CI* | *P-value* |  | *P-value* |
| Age (per year) | 1.03 | (1.01, 1.05) | 0.0019 |  | 1.02 | (0.997, 1.05) | 0.083 |  | 0.020 |
| IREG (+) vs. (-) | 2.23 | (1.32, 3.83) | 0.0025 |  | 1.66 | (0.889, 3.12) | 0.11 |  |  |
