# Supplementary material for: Tumor Endothelial Inflammation Predicts Clinical Outcome in Diverse Human Cancers
Source: PLoS One. 2012 Oct 4;7(10):e46104. doi: 10.1371/journal.pone.0046104 (PMC3464251; doi:10.1371/journal.pone.0046104)
Supplement: Table S10 — Univariate Cox proportional hazards model of overall survival using the IREG gene signature in patient subgroups. Shown are the hazard ratios (HR), 95% confidence intervals (CI), and p-values. (DOC) [file pone.0046104.s016.doc]

|  |  |  | IREG+ vs. IREG- |  |
| --- | --- | --- | --- | --- |
|  |  | *HR* | *95% CI* | *P-value* |
| **Breast cancer** |  |  |  |  |
| Age (years) |  |  |  |  |
| < 40 |  | 2.45 | 1.17-5.47 | 0.017 |
| ≥ 40 |  | 2.38 | 1.33-4.45 | 0.0032 |
|  |  |  |  |  |
| Tumor size |  |  |  |  |
| ≥ T2 |  | 2.78 | 1.47-5.72 | 0.0012 |
|  |  |  |  |  |
| Lymph nodes |  |  |  |  |
| Uninvolved |  | 1.85 | 1.01-3.51 | 0.048 |
| Involved |  | 3.15 | 1.57-6.86 | 0.0011 |
|  |  |  |  |  |
| **Colon cancer** |  |  |  |  |
| Age (years) |  |  |  |  |
| < 60 |  | 2.33 | 1.12-5.07 | 0.024 |
| ≥ 60 |  | 2.00 | 1.21-3.38 | 0.0071 |
|  |  |  |  |  |
| Stage |  |  |  |  |
| I or II |  | 2.45 | 0.98-6.61 | 0.056 |
| III or IV |  | 1.90 | 1.19-3.09 | 0.0068 |
|  |  |  |  |  |
| **Glioma** |  |  |  |  |
| Age (years) |  |  |  |  |
| < 55 |  | 2.35 | 1.29-4.30 | 0.0053 |
|  |  |  |  |  |
| **Lung cancer** |  |  |  |  |
| Age (years) |  |  |  |  |
| < 65 |  | 1.89 | 1.27-2.86 | 0.0015 |
| ≥ 65 |  | 1.42 | 1.00-2.01 | 0.048 |
|  |  |  |  |  |
| Lymph nodes |  |  |  |  |
| Involved |  | 2.01 | 1.35-3.05 | <0.001 |
|  |  |  |  |  |
| Tumor size |  |  |  |  |
| ≤ T2 |  | 1.56 | 1.18-2.08 | 0.0018 |
