# Supplementary material for: Tumor Endothelial Inflammation Predicts Clinical Outcome in Diverse Human Cancers
Source: PLoS One. 2012 Oct 4;7(10):e46104. doi: 10.1371/journal.pone.0046104 (PMC3464251; doi:10.1371/journal.pone.0046104)
Supplement: Table S11 — Primers for quantitative RT-PCR analysis of human endothelial inflammatory gene expression. (DOC) [file pone.0046104.s017.doc]

| **Human Gene** | **Direction** | **Primer sequence** |
| --- | --- | --- |
| CXCL10 | Forward | TCCTGCAAGCCAATTTTGTCCACG |
| CXCL10 | Reverse | GCACTGCATCGATTTTGCTCCCC |
| GAPDH | Forward | CTCTGCTCCTCCTGTTCGAC |
| GAPDH | Reverse | GTTAAAAGCAGCCCTGGTGA |
| ICAM1 | Forward | ACGGATGCCAGCTTGGGCAC |
| ICAM1 | Reverse | GGGAGCTCCGTGAGGCCAGA |
| IFI44 | Forward | GACAGAGCAGCTACCCTCAGCT |
| IFI44 | Reverse | AGGCTAAGCCGCTTCCCTCCAA |
| IRF7 | Forward | TACCATCTACCTGGGCTTCG |
| IRF7 | Reverse | GCTCCATAAGGAAGCACTCG |
| SELE | Forward | ACTGGGCTCCAGGTGAACCCA |
| SELE | Reverse | TCACCGTGGCCACTGCAGGA |
| STAT1 | Forward | ACCGCACCTTCAGTCTTTTCC |
| STAT1 | Reverse | TGAACTGGACCCCTGTCTTCA |
| TAP1 | Forward | TTCTCAGACCTGCGCACTCCATCTC |
| TAP1 | Reverse | CTGGCCACACCAAAGCATCAGCC |
| VCAM1 | Forward | GGGGACCACATCTACGCTGACAATG |
| VCAM1 | Reverse | CAGCCTCCAGAGGGCCACTCAAA |
